# Supplementary material for: Human Pericardial Fluid Contains Exosomes Enriched with Cardiovascular-Expressed MicroRNAs and Promotes Therapeutic Angiogenesis
Source: Mol Ther. 2017 Feb 1;25(3):679–93. doi: 10.1016/j.ymthe.2016.12.022 (PMC5363195; doi:10.1016/j.ymthe.2016.12.022)

YMTHE, Volume *25*

Supplemental Information

Human Pericardial Fluid Contains Exosomes Enriched with Cardiovascular-Expressed MicroRNAs and Promotes Therapeutic Angiogenesis

Cristina Beltrami, Marie Besnier, Saran Shantikumar, Andrew I.U. Shearn, Cha Rajakaruna, Abas Laftah, Fausto Sessa, Gaia Spinetti, Enrico Petretto, Gianni D. Angelini, and Costanza Emanueli

Supplemental Figures

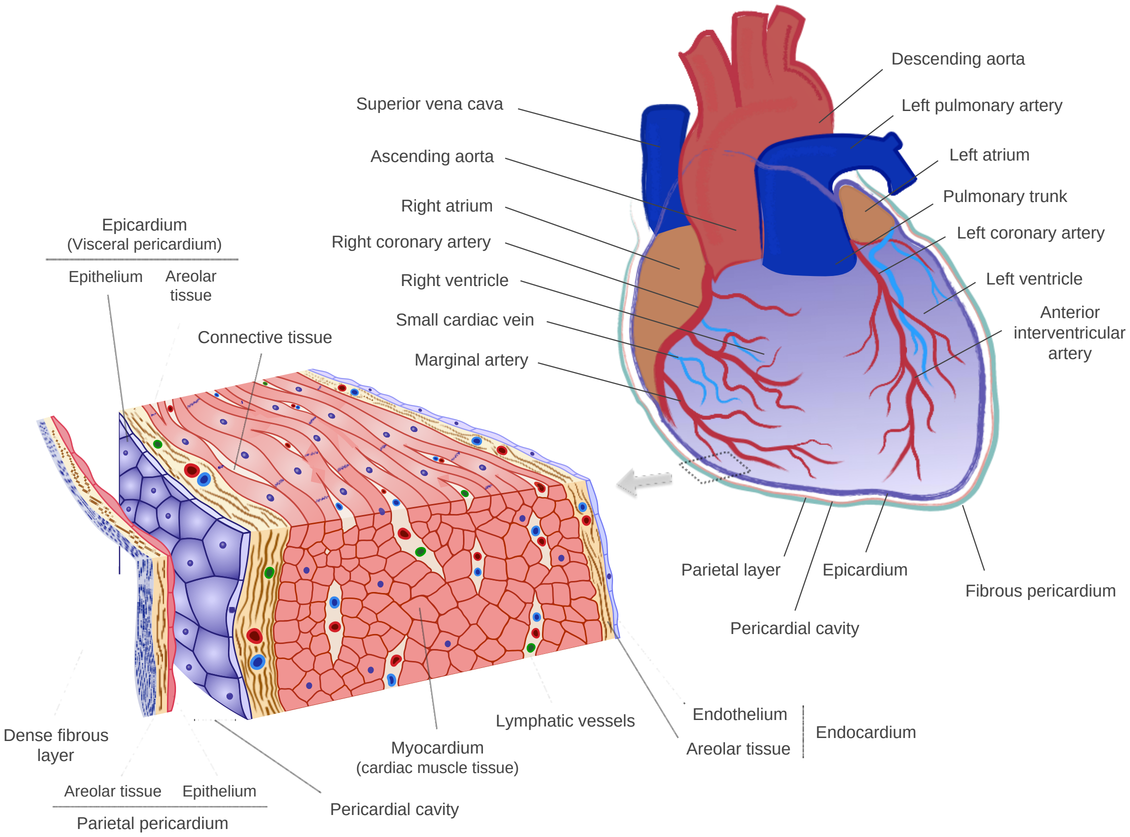

Figure S1. Internal anatomy of the heart. The heart contains three layers: the superficial epicardium; the middle myocardium; and the inner endocardium. The pericardial fluid is contained within the double-walled pericardial sac (also known as pericardium) that surrounds the heart and the roots of the great vessels bringing blood to and from the heart cells, namely the superior and inferior vena cava; pulmonary arteries and pulmonary veins. The pericardium is composed of two layers: 1) the superficial fibrous pericardium, comprised of connective tissue, which is continuous with the tunica adventitia of the great blood vessels and anchors the heart to the surrounding walls; and 2) the serous pericardium composed of mesothelial cells. The serous pericardium is in turn formed by a parietal layer that fuses with the fibrous pericardium and a visceral layer (epicardium) and the epicardium, which sits on and signals to the myocardium. The pericardial fluid (PF) is secreted by the serous membranes and obtained by both capillary permeability and hydrostatic/osmotic pressure from the epicardium and from the interstitial fluid underlying the myocardium.

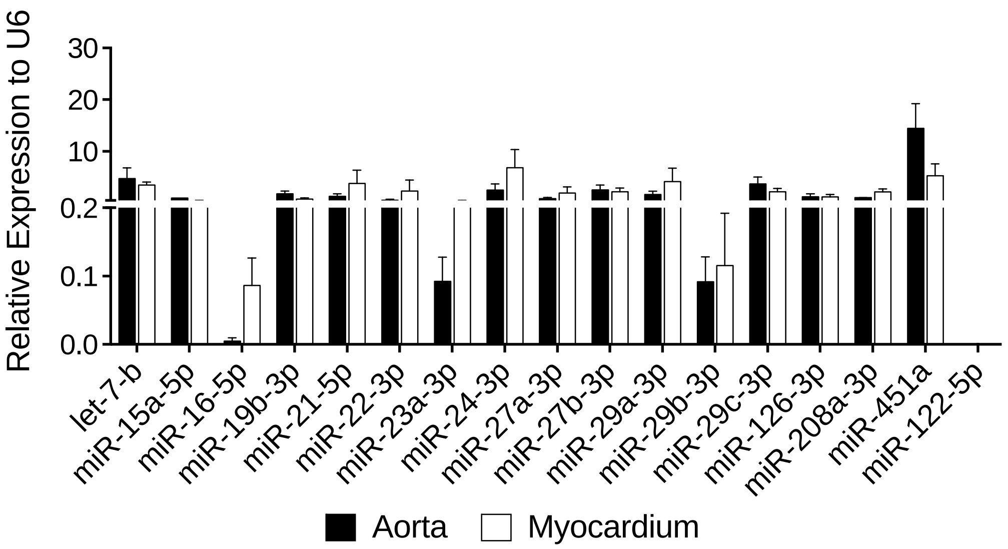

Figure S2. MicroRNA (miRNA) expression in ascending aorta (aorta) and right atrial appendage (myocardium) samples collected from the surgical patients. The cardiovascular expression of selected miRNAs was confirmed by RT-qPCR analyses in the available ascending aorta (n=5-7) and right atrium (n=3-4) clinical samples collected as leftover material from the patients undergoing aortic valve replacement (AVR). U6 was used as the endogenous control. All values are mean + s.e.m.

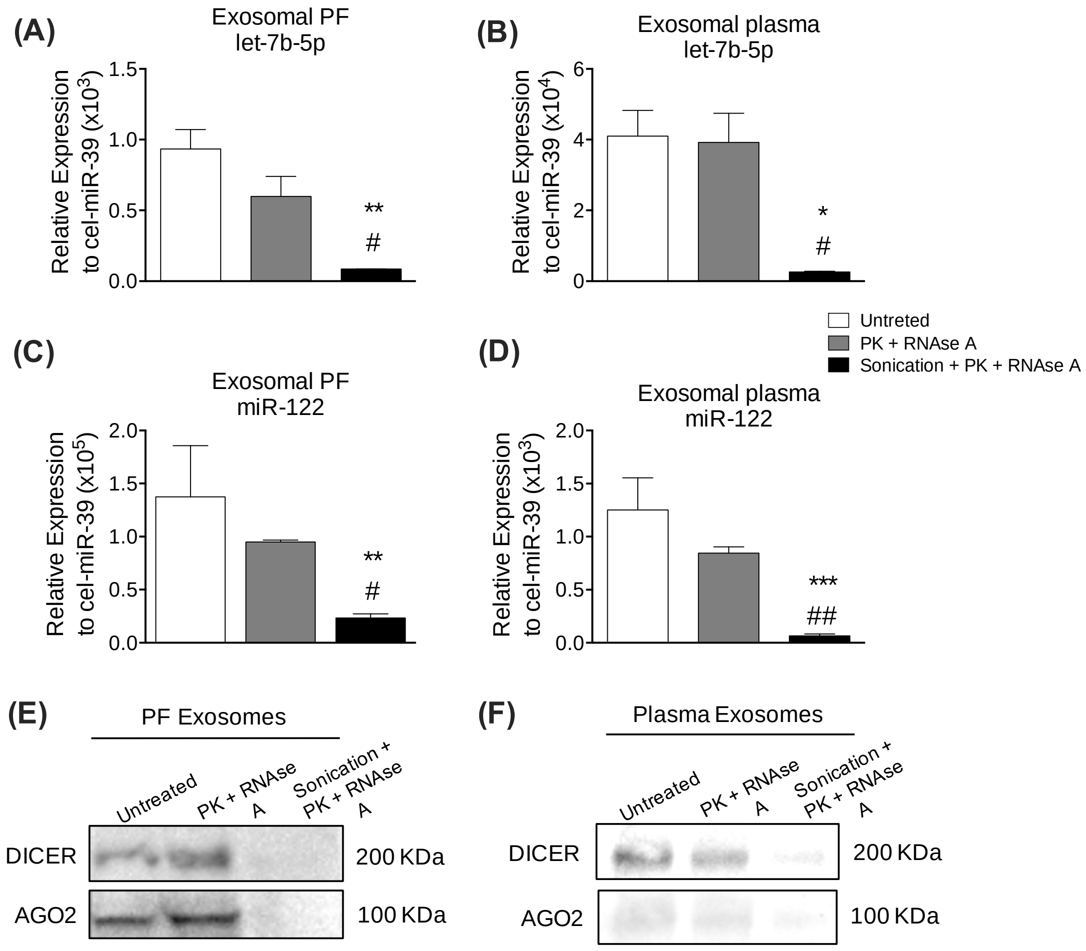

Figure S3. Effect of exosomes treatment with Proteinase K and RNAse A on DICER, AGO-2 and microRNAs. Exosomes enriched from either the pericardial fluid (PF) or plasma were submitted, or not, to sonication to break the exosome membrane. Next, sonicated or intact exosomes were incubated with proteinase K (PK; 50 µg/ml) and RNAse A (100 µg/ml). A control group (Untreated) consisted of non-sonicated exosomes not receiving PK/RNAse A. Exosomal let-7b and miR-122 expression in **(A,C)** PF or **(B,D)** plasma were measured by RT-qPCR using spike-in cel-miR-39 as a normalizer. Representative Western blot images of DICER and AGO-2 protein incorporated in the **(E)** PF and **(F)** plasma exosomes. *P≤0.05, **P<0.01 and ***P ≤ 0.001 vs. untreated, # P≤0.05 and ## P≤0.01 vs. treatment with PK and RNAse A. Statistical significance was tested using one-way ANOVA with Tukey's *post hoc* test. All values are mean + s.e.m; n=3.

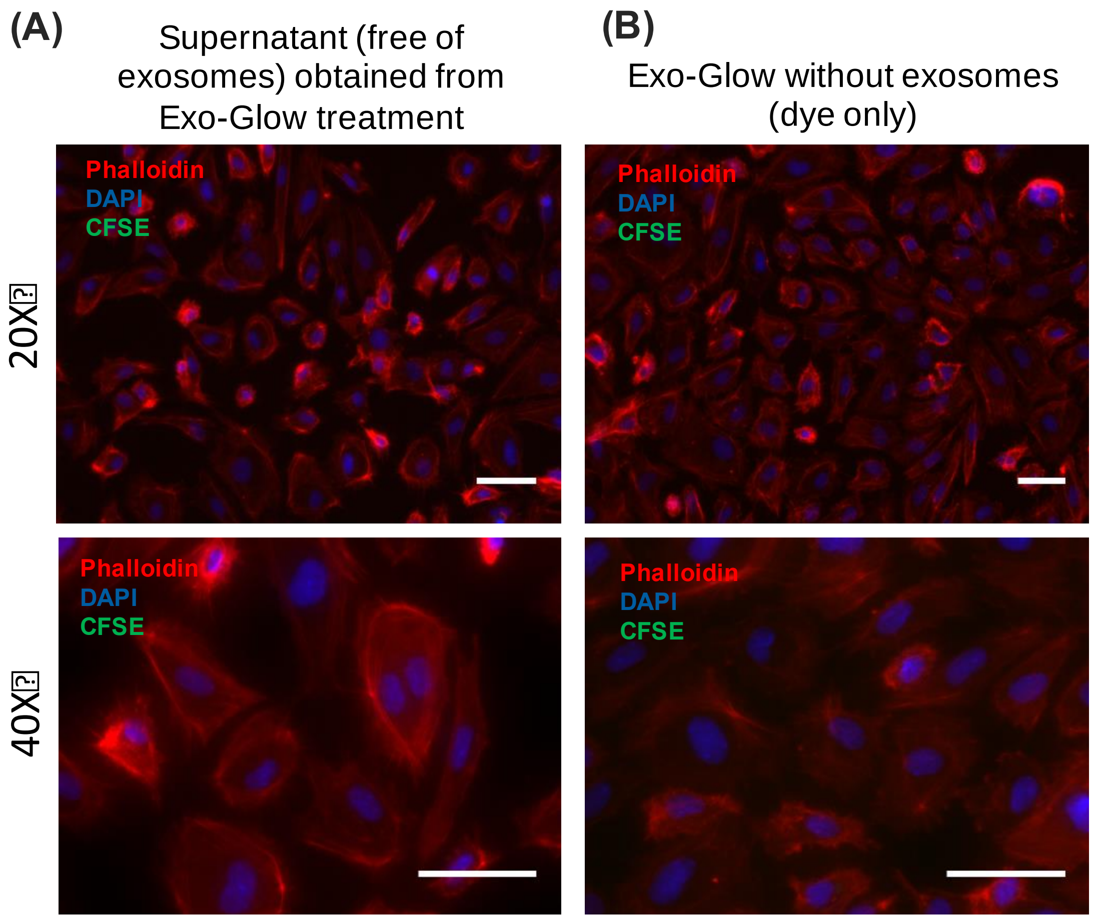

Figure S4. Control for exosome incorporation staining. Endothelial cells (ECs) were cultured for 24hrs with **(A)** supernatant obtained from PF-derived exosomes stained with CFSE (green fluorescence) or **(B)** the CGSE dye used for the exosome staining. Cells were stained with phalloidin (in red) and DAPI (blue). (Scale bar, 25 μm).

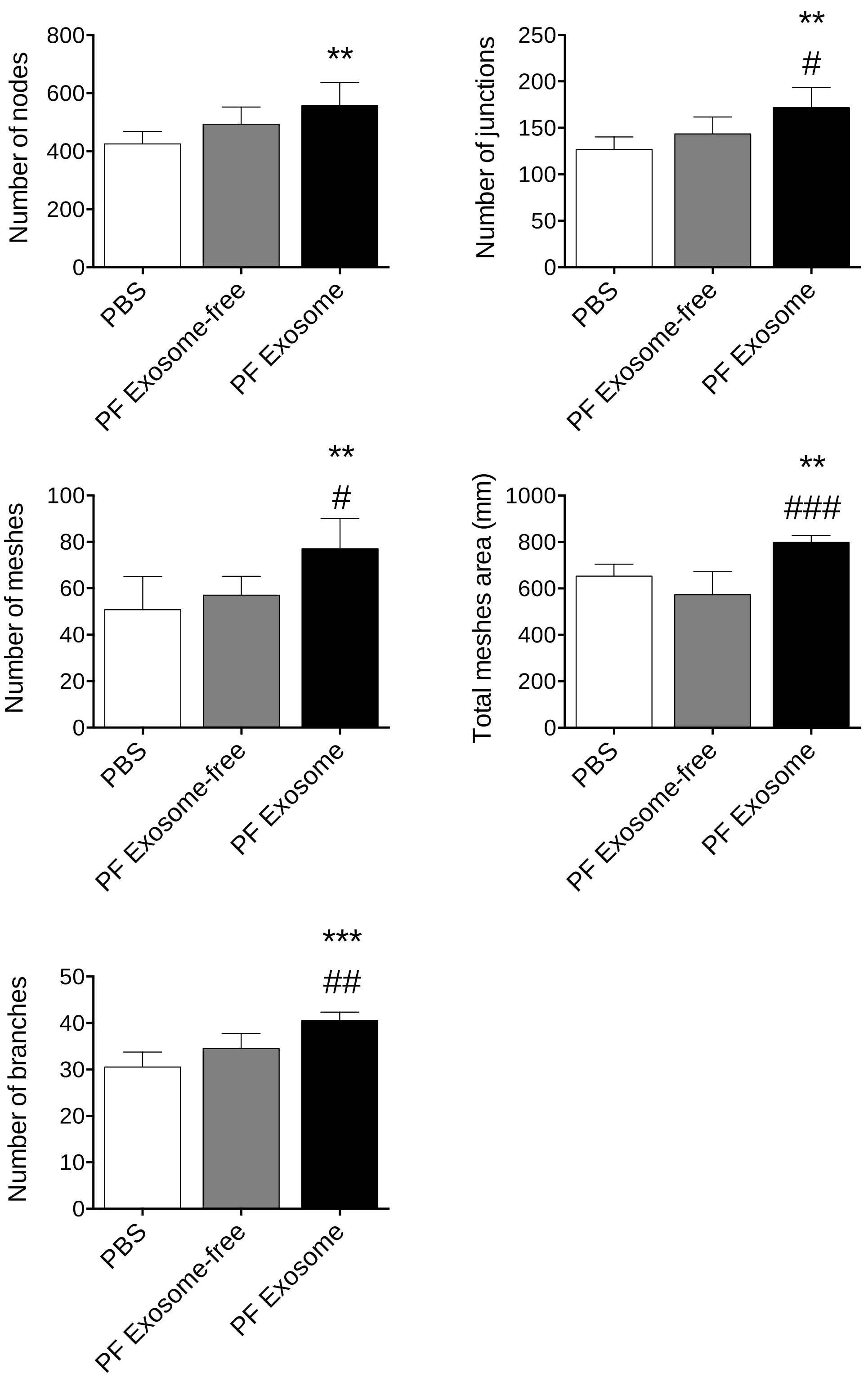

Figure S5. Matrigel assay quantification showing additional parameters to those presented in Fig.5E. In Figure 5E, we present the total length of tube-like structure of the Matrigel assays. Here, we show additional parameters derived from the quantification of the assays. All values are mean + s.e.m. n=5. ** $P \leq 0.01$ and *** $P \leq 0.001$; vs. PBS; $^{\#}P \leq 0.05$; $^{\#\#}P \leq 0.01$ and $^{\#\#\#}P \leq 0.001$; vs. Exosome-free PF.

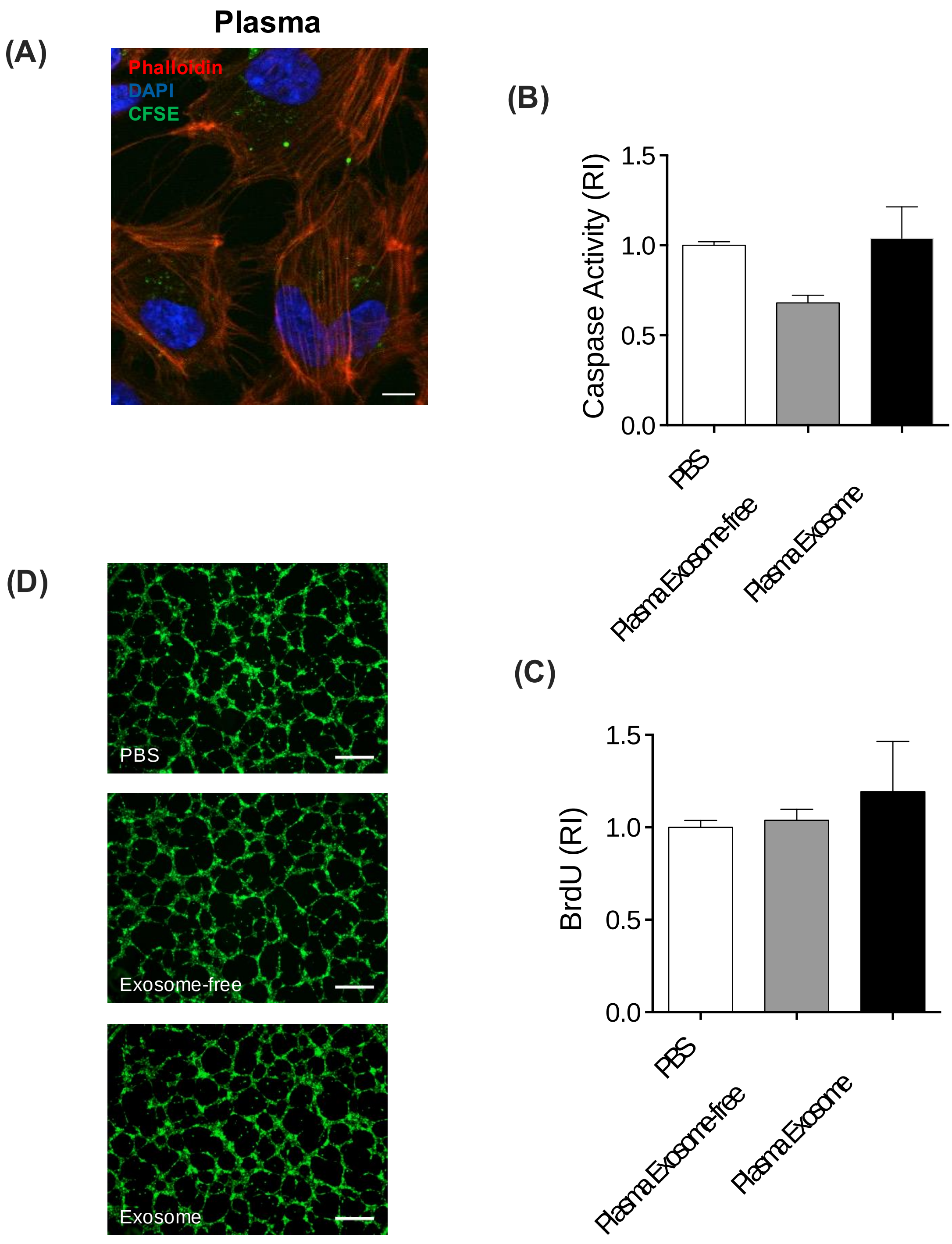
(A)
Plasma
Phalloidin
DAPI
CFSE
(B)
Caspase Activity (RI)
1.5
1.0
0.5
0.0
PBS
Plasma Exosome-free
Plasma Exosome
(C)
BrdU (RI)
1.5
1.0
0.5
0.0
PBS
Plasma Exosome-free
Plasma Exosome
(D)
PBS
Exosome-free
Exosome

(E)

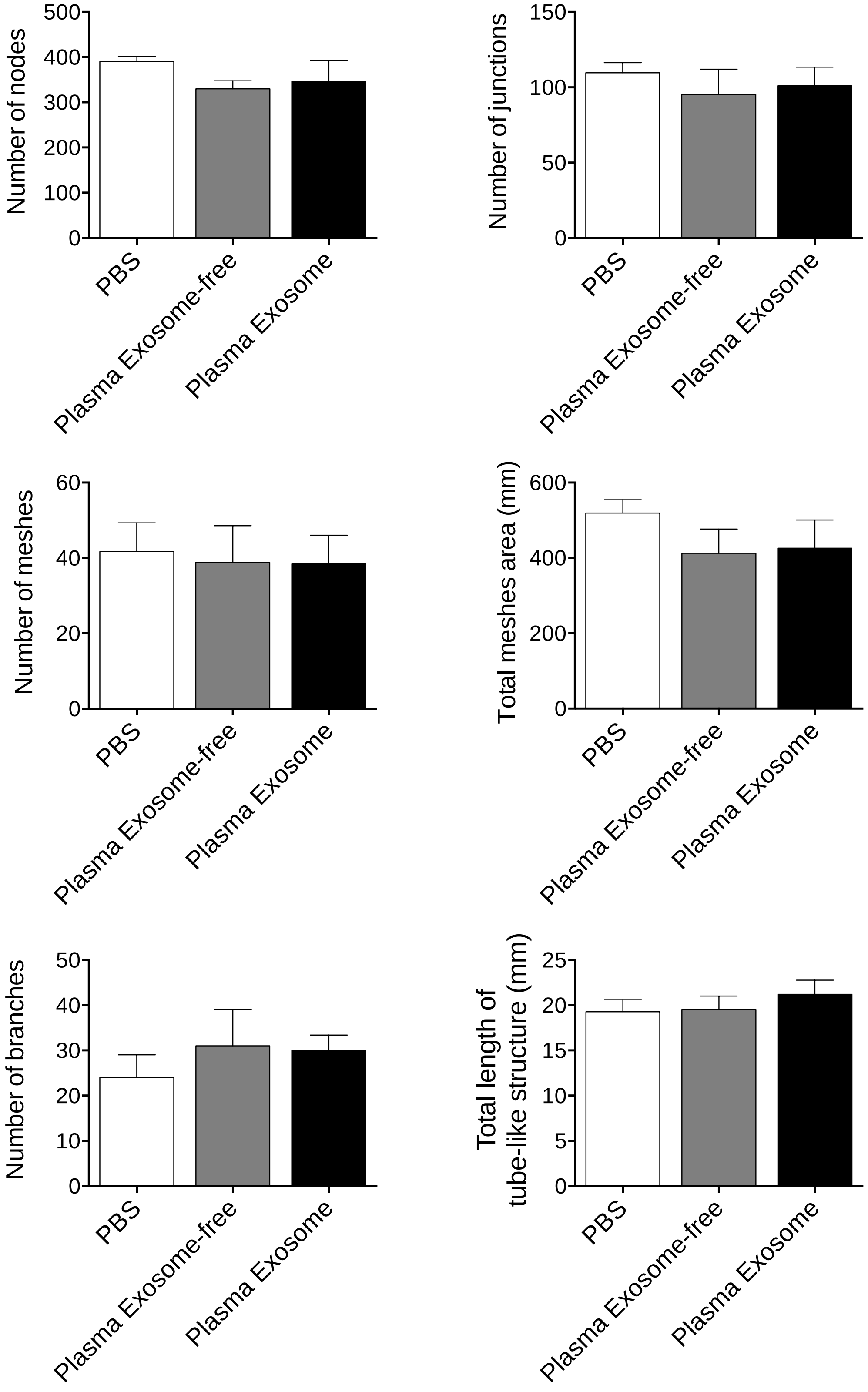
Number of nodes
500
400
300
200
100
0
Number of junctions
150
100
50
0
Number of meshes
60
40
20
0
Total meshes area (mm)
600
400
200
0
Number of branches
50
40
30
20
10
0
Total length of
tube-like structure (mm)
25
20
15
10
5
0
PBS
Plasma Exosome-free
Plasma Exosome

(F)

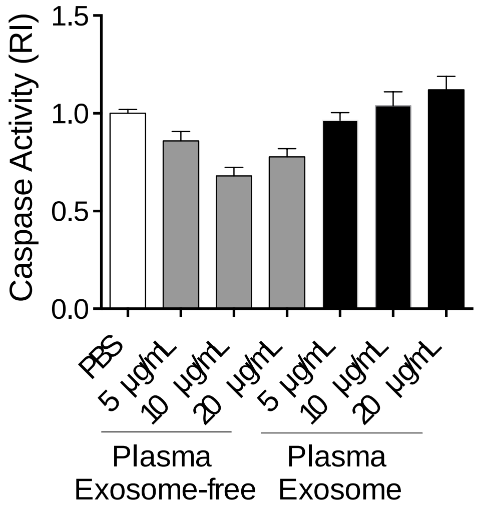

(G)

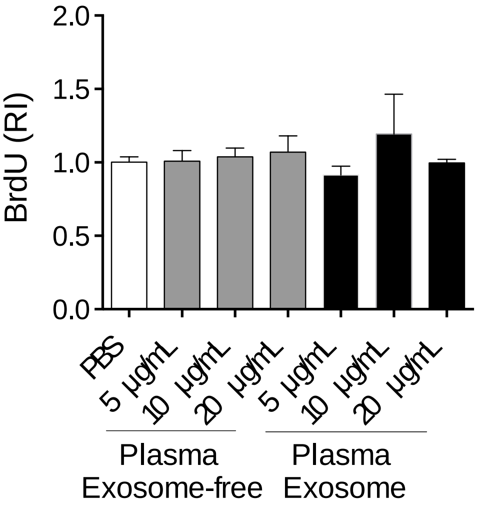

(H)

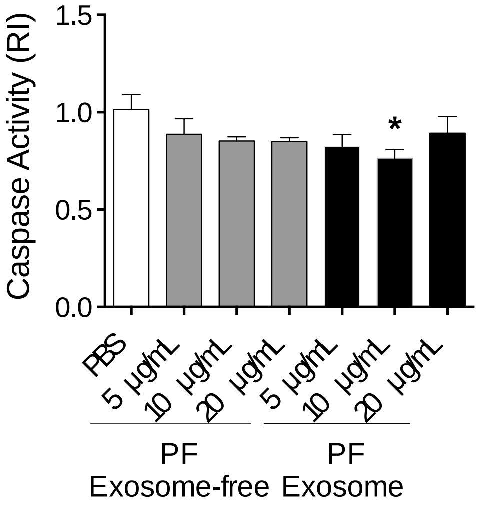

(I)

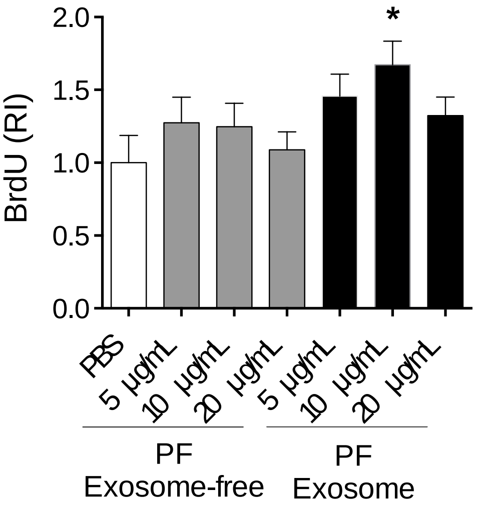

Figure S6. Plasma exosomes are incorporated by cultured ECs, but they do not enhance their angiogenic capacity. (A) Plasma-derived exosomes were stained using CFSE (in green) and incubated (10 µg/ml) with cultured ECs for 24 hrs. Cells were stained with phalloidin (in red) and DAPI (blue) (scale bar, 25 µm). Column graphs show **(B,F)** caspase activity and **(C,G)** BrdU incorporation in ECs treated (24h) with 5, 10 and 20 µg/ml of either plasma-derived exosomes (black columns) or the same concentrations of exosome-depleted plasma. **(D)** Photomicrograph shows the EC network formation on Matrigel (scale bar, 200 µm) while in **(E)** the bar graphs show total length of tube-like structures of ECs treated as previously described; n=5. 2.5X Magnification. In **(H)** and **(I)**, we show the results of apoptosis and proliferation (concentrations-responses) assays in ECs stimulated with PF exosomes. Statistical significance was tested using one-way ANOVA with Dunnett's *post hoc* test. All values are mean+ s.e.m; n=7. *P ≤ 0.05 vs. PBS.

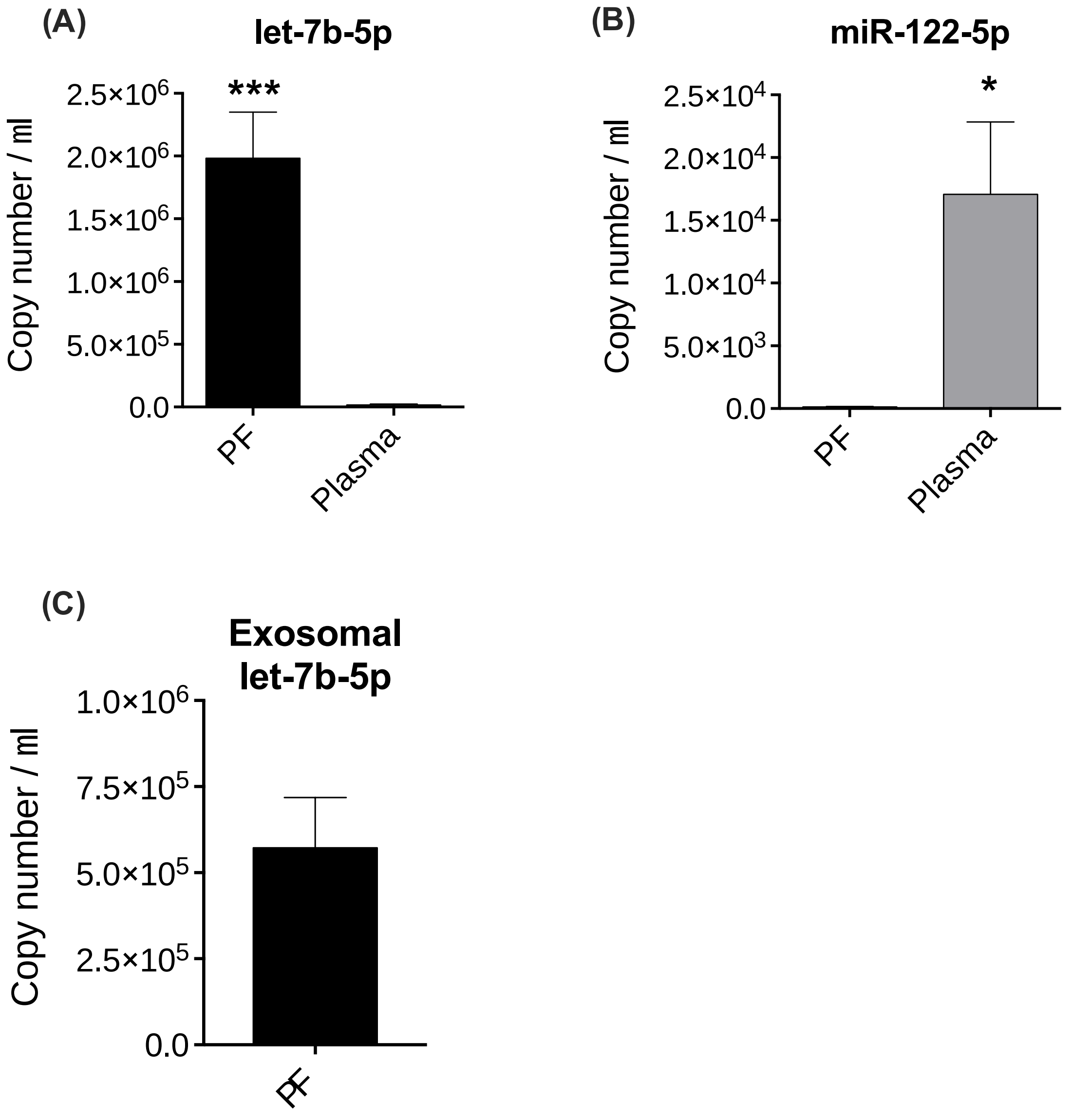

Figure S7. Let-7b-5p expression in total and exosomal PF and plasma samples. Absolute expression of **(A)** let-7b-5p (***P ≤ 0.001 vs plasma) and **(B)** miR-122-5p (*P ≤ 0.05 vs PF) expressed as copy number/µl of fluid was detected in total PF and plasma samples; n=5. **(C)** Copy number/µl of let-7b-5p in the PF exosomes. Unpaired two-tailed Student's *t-test* was applied in (A) and (B). All values are mean + s.e.m.

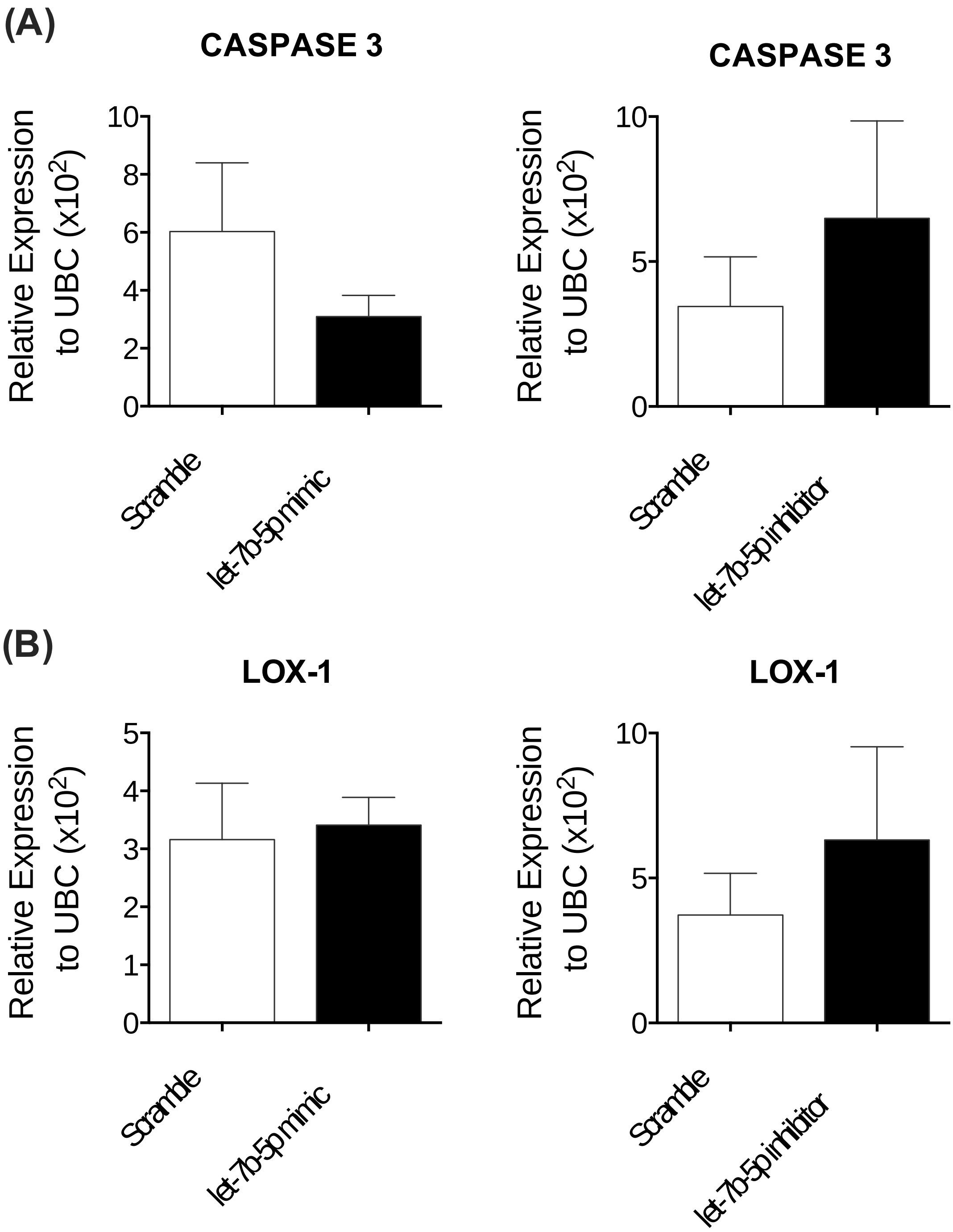

Figure S8. Expression of the previously validated direct target genes of let-7b-5p, *CASPASE-3* and *LOX1*, in ECs after let-7b-5b changes. ECs were transfected with either a mimic of let-7b-5p (right panel) or a let-7b-5p inhibitor (left panel) or the respective scramble sequences. The relative expression of the qPCR is shown using *UBC* as the normalizer. All values are mean + s.e.m; n=4.

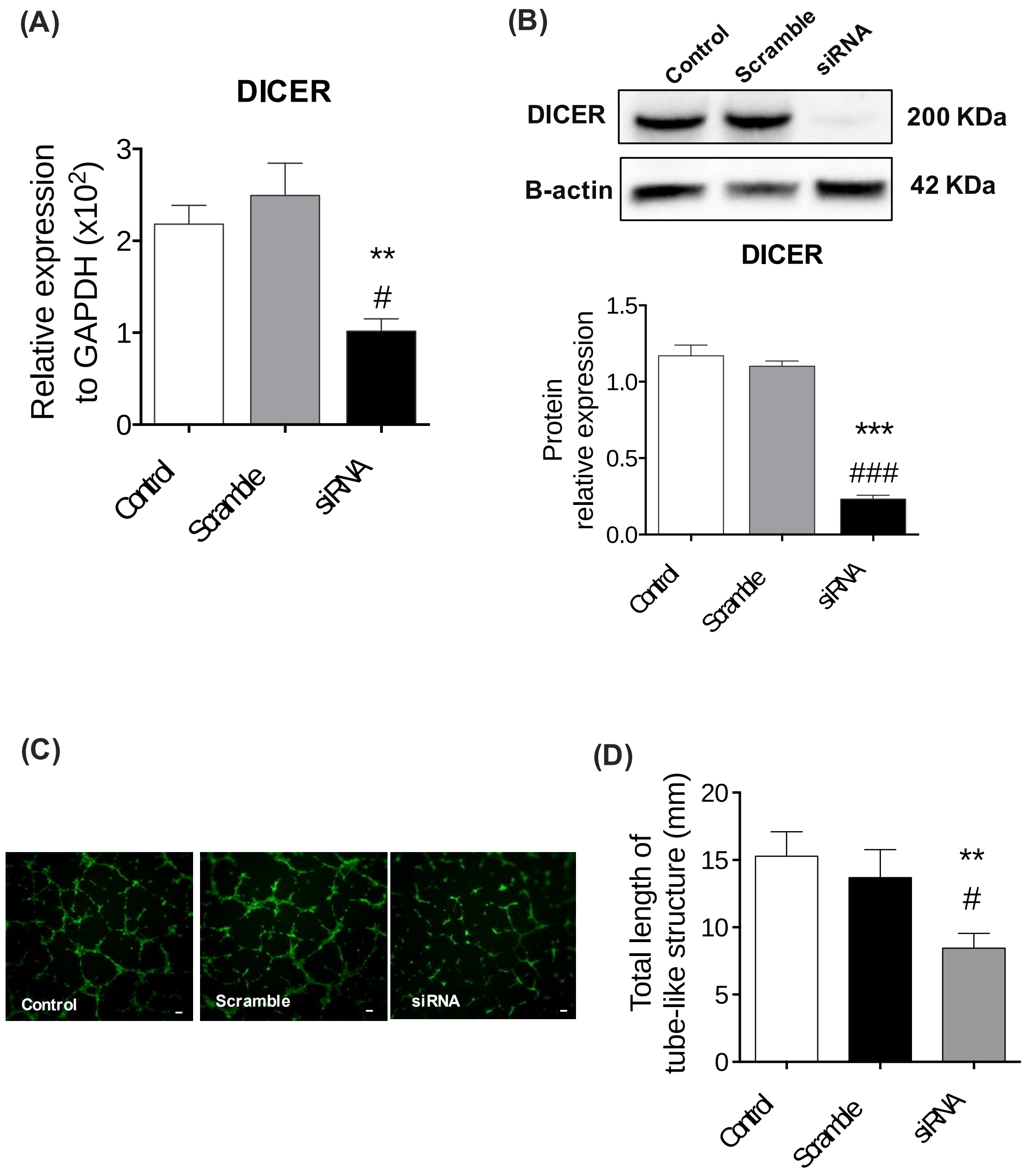

Figure S9. Consequences of Dicer RNA silencing in ECs. ECs were transfected with either Dicer siRNA or a scramble sequence for 24 hrs. Control cells were left untouched. Transfection efficiency was assessed by measuring Dicer mRNA and protein expression by using **(A)** RT–qPCR and **(B)** Western blotting (WB), respectively. An antibody against actin was used as the loading control in the WB. **(C)** Photomicrographs show the endothelial network formation on Matrigel (scale bar 100 µm) and **(D)** bar graphs show the total length of tube-like structures of ECs transfected as previously indicated. Data are presented as mean + s.e.m. from 4 independent experiments. Statistical significance was tested using one-way ANOVA with Dunnett's *post hoc* test. $^{\#}P \leq 0.05$ and $^{\#\#\#}P \leq 0.001$ vs non-transfected control; $^{**}P \leq 0.01$ and $^{***}P \leq 0.001$ vs. scrambles;

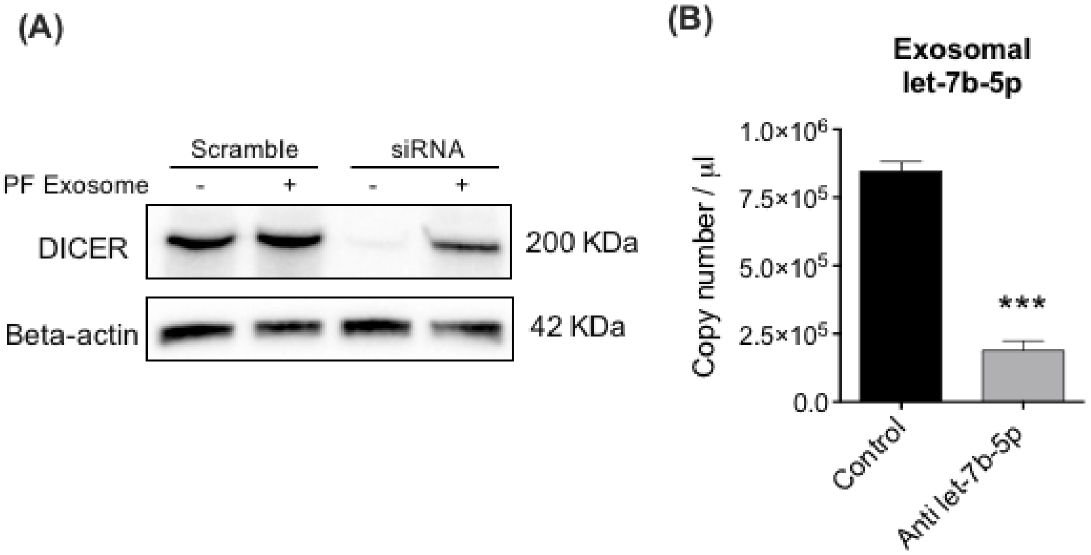

Figure S10. PF exosomes restore Dicer protein levels in ECs after DICER KD, and exosomal let-7b-5p can be inhibited by transfection with anti-let-7b-5p. (A) Western blotting showing the restoration of DICER protein after treatment with PF-derived exosomes (10 µg/ml) of ECs that were transfected with *DICER* siRNA (siRNA) or scramble siRNA (scramble). Antibody against actin was used as loading control; n=3. **(B)** Efficiency of let-7b-5p inhibitor transfection in PF exosomes; n=6. Unpaired two-tailed Student's *t-test* was applied. All values are mean + s.e.m. *** $P \leq 0.001$ vs. control.

Tables

Table S1: Characteristics of the aortic valve replacement (AVR) patients used in the study

(NYHA = New York Heart Association; LVEF = left ventricular ejection fraction)

Characteristic	Total n=16	
Age (Years; mean, SD)		71.7 (7.6)
Sex (males;n,%)		11/16 (68.7%)
Total volume of PF collected (ml; mean, SD)		6.4 (3.8)
Diabetes (n,%)		2/14 (14.2%)
Hypertension (n,%)		14/14 (100%)
Valve stenosis (n,%)		16/16 (100%)
NYHA Class (n,%)	Class 2	14/15 (93.3%)
	Class 3	1/15 (6.7%)
LVEF	Good	14/15 (93.3%)
	Moderate	1/15 (6.6%)

Table S2. List of the microRNAs (miRNAs) expressed in the pericardial fluid (PF) samples.

A PCR-based miRNA array (ID: 203616,Exiqon) was developed using non-pooled pericardial fluid (PF) samples, each taken from a different patient undergoing aortic valve replacement (AVR) surgery. Of the 752 miRNAs present in the array, 359 miRNAs (shown below with their PCR Ct) were expressed by each tested sample. Of these 358 miRNAs, the ones that were already known to be expressed in cardiovascular tissues and cells are in red and additionally underlined when selected for further validation within this study. The particulars of the 16 putative cardiovascular miRNAs selected for further investigations are shown in the Table S3.

PF- sample 1		PF-sample 2		PF-sample 3	
miRNAs	Ct	miRNAs	Ct	miRNAs	Ct
hsa-miR-16-5p	23.12	hsa-miR-451a	16.49	hsa-miR-451a	16.606
hsa-miR-21-5p	23.19	hsa-miR-16-5p	17	hsa-miR-21-5p	20.256
hsa-miR-451a	23.4	hsa-miR-21-5p	17.08	hsa-miR-125b-5p	20.316
hsa-miR-125b-5p	23.58	hsa-miR-125b-5p	17.93	hsa-miR-24-3p	20.386
hsa-miR-23a-3p	24.82	hsa-miR-24-3p	19.12	hsa-miR-19b-3p	20.466
hsa-miR-24-3p	25.08	hsa-miR-29a-3p	19.28	hsa-miR-29a-3p	20.656
hsa-miR-27b-3p	25.41	hsa-miR-29c-3p	19.52	hsa-miR-223-3p	20.846
hsa-let-7b-5p	25.54	hsa-miR-27b-3p	19.55	hsa-miR-22-3p	20.926
hsa-miR-29a-3p	25.86	hsa-miR-22-3p	19.62	hsa-miR-15a-5p	21.036
hsa-miR-19b-3p	26.04	hsa-miR-23a-3p	19.66	hsa-miR-23a-3p	21.176
hsa-miR-29c-3p	26.06	hsa-miR-19b-3p	20.04	hsa-miR-29c-3p	21.186
hsa-miR-23b-3p	26.08	hsa-let-7b-5p	20.06	hsa-miR-99a-5p	21.296
hsa-miR-100-5p	26.28	hsa-miR-15a-5p	20.31	hsa-miR-23b-3p	21.336
hsa-miR-320a	26.3	hsa-miR-23b-3p	20.58	hsa-miR-320b	21.386
hsa-miR-22-3p	26.39	hsa-miR-148a-3p	20.58	hsa-let-7b-5p	21.476
hsa-miR-15a-5p	26.44	hsa-miR-29b-3p	20.72	hsa-miR-27b-3p	21.606
hsa-let-7a-5p	26.49	hsa-miR-34a-5p	20.84	hsa-miR-365a-3p	21.806
hsa-miR-148a-3p	26.56	hsa-miR-99a-5p	20.87	hsa-miR-92a-3p	22.036
hsa-miR-99a-5p	26.78	hsa-miR-320a	21.15	hsa-miR-29b-3p	22.286
hsa-miR-221-3p	26.84	hsa-let-7a-5p	21.22	hsa-miR-140-3p	22.286
hsa-miR-34a-5p	27.11	hsa-miR-221-3p	21.44	hsa-miR-27a-3p	22.396
hsa-miR-92a-3p	27.15	hsa-miR-20a-5p	21.46	hsa-miR-106a-5p	22.396
hsa-miR-378a-3p	27.24	hsa-miR-378a-3p	21.47	hsa-miR-20a-5p	22.526
hsa-miR-150-5p	27.31	hsa-miR-152	21.51	hsa-let-7g-5p	22.556
hsa-miR-125a-5p	27.32	hsa-miR-101-3p	21.6	hsa-miR-130a-3p	22.826
hsa-miR-365a-3p	27.39	hsa-miR-30e-5p	21.66	hsa-miR-151a-5p	22.926
hsa-miR-20a-5p	27.44	hsa-miR-100-5p	21.74	hsa-miR-320a	22.936
hsa-miR-30e-5p	27.59	hsa-miR-27a-3p	21.79	hsa-miR-93-5p	23.276
hsa-miR-423-5p	27.6	hsa-miR-140-3p	21.79	hsa-miR-125a-5p	23.416
hsa-miR-361-5p	27.6	hsa-let-7c	21.88	hsa-miR-342-3p	23.436
hsa-let-7d-3p	27.8	hsa-let-7i-5p	21.89	hsa-miR-103a-3p	23.476
hsa-miR-152	27.81	hsa-miR-93-5p	21.97	hsa-miR-574-3p	23.786
hsa-miR-204-5p	27.84	hsa-miR-92a-3p	21.99	hsa-miR-181a-5p	23.846
hsa-miR-423-3p	27.84	hsa-miR-186-5p	22.01	hsa-miR-423-5p	23.876
hsa-let-7g-5p	27.85	hsa-miR-365a-3p	22.06	hsa-miR-423-3p	23.926
hsa-miR-26a-5p	27.9	hsa-miR-199a-3p	22.13	hsa-miR-532-3p	23.946
hsa-miR-101-3p	27.92	hsa-miR-130a-3p	22.23	hsa-miR-25-3p	24.146
hsa-miR-186-5p	27.95	hsa-miR-223-3p	22.27	hsa-let-7c	24.176
hsa-let-7i-5p	28.01	hsa-miR-106a-5p	22.47	hsa-miR-28-3p	24.336
hsa-miR-27a-3p	28.03	hsa-miR-423-5p	22.48	hsa-miR-99b-5p	24.346
hsa-miR-130a-3p	28.04	hsa-miR-195-5p	22.48	hsa-miR-424-5p	24.406

hsa-miR-99b-5p	28.06	hsa-miR-99b-5p	22.49	hsa-miR-378a-3p	24.426
hsa-let-7c	28.07	hsa-miR-148b-3p	22.49	hsa-miR-191-5p	24.526
hsa-miR-93-5p	28.12	hsa-miR-25-3p	22.58	hsa-miR-26a-5p	24.546
hsa-miR-223-3p	28.16	hsa-let-7g-5p	22.68	hsa-miR-324-3p	24.556
hsa-miR-140-3p	28.18	hsa-miR-125a-5p	22.72	hsa-miR-532-5p	24.606
hsa-miR-30b-5p	28.24	hsa-miR-361-5p	22.76	hsa-miR-15b-5p	24.636
hsa-miR-199a-3p	28.24	hsa-miR-497-5p	22.8	hsa-let-7d-3p	24.796
hsa-miR-195-5p	28.35	hsa-miR-95	22.8	hsa-miR-34a-5p	24.856
hsa-miR-25-3p	28.41	hsa-miR-151a-5p	22.81	hsa-miR-199a-3p	24.876
hsa-miR-29b-3p	28.45	hsa-miR-660-5p	22.83	hsa-miR-30b-5p	24.886
hsa-miR-106a-5p	28.5	hsa-miR-26a-5p	22.93	hsa-miR-101-3p	24.906
hsa-miR-148b-3p	28.53	hsa-miR-335-5p	22.96	hsa-miR-140-5p	25.026
hsa-miR-497-5p	28.6	hsa-miR-335-3p	22.97	hsa-miR-551a	25.076
hsa-miR-342-3p	28.67	hsa-miR-15b-5p	23.06	hsa-miR-151a-3p	25.096
hsa-miR-151a-5p	28.67	hsa-miR-320b	23.06	hsa-miR-497-5p	25.286
hsa-miR-146a-5p	28.74	hsa-miR-140-5p	23.08	hsa-let-7d-5p	25.286
hsa-miR-320b	28.76	hsa-miR-218-5p	23.12	hsa-miR-126-5p	25.376
hsa-miR-126-3p	28.83	hsa-miR-22-5p	23.15	hsa-miR-126-3p	25.416
hsa-miR-30c-5p	28.89	hsa-miR-30a-5p	23.19	hsa-miR-30a-3p	25.426
hsa-miR-103a-3p	28.9	hsa-miR-181a-5p	23.27	hsa-miR-193a-5p	25.466
hsa-miR-30a-5p	29.02	hsa-miR-103a-3p	23.34	hsa-miR-150-5p	25.516
hsa-miR-22-5p	29.06	hsa-miR-30b-5p	23.45	hsa-miR-148b-3p	25.556
hsa-miR-140-5p	29.1	hsa-miR-486-5p	23.49	hsa-miR-221-3p	25.636
hsa-miR-95	29.13	hsa-miR-143-3p	23.6	hsa-miR-22-5p	25.636
hsa-miR-486-5p	29.23	hsa-miR-185-5p	23.67	hsa-miR-146a-5p	25.636
hsa-miR-335-5p	29.25	hsa-miR-106b-5p	23.71	hsa-miR-193b-3p	25.646
hsa-miR-660-5p	29.33	hsa-miR-423-3p	23.75	hsa-miR-30c-5p	25.646
hsa-miR-193a-5p	29.41	hsa-miR-342-3p	23.77	hsa-miR-502-3p	25.706
hsa-miR-574-3p	29.46	hsa-miR-199a-5p	23.82	hsa-let-7i-5p	25.726
hsa-let-7f-5p	29.67	hsa-miR-126-3p	23.86	hsa-miR-152	25.746
hsa-miR-126-5p	29.77	hsa-let-7d-3p	23.86	hsa-miR-200c-3p	25.786
hsa-let-7d-5p	29.8	hsa-miR-146a-5p	23.87	hsa-miR-31-5p	25.806
hsa-miR-532-3p	29.83	hsa-miR-150-5p	23.94	hsa-miR-486-5p	25.826
hsa-miR-200b-3p	29.88	hsa-miR-532-3p	23.94	hsa-miR-95	25.876
hsa-miR-218-5p	30.06	hsa-miR-193a-5p	23.98	hsa-miR-210	25.906
hsa-miR-28-3p	30.06	hsa-miR-574-3p	23.99	hsa-miR-224-3p	25.906
hsa-miR-31-5p	30.11	hsa-miR-324-3p	24.05	hsa-miR-200b-3p	25.926
hsa-miR-181a-5p	30.13	hsa-miR-210	24.18	hsa-miR-374a-5p	25.986
hsa-miR-324-3p	30.14	hsa-miR-424-5p	24.21	hsa-miR-145-5p	26.036
hsa-miR-106b-5p	30.18	hsa-miR-362-3p	24.21	hsa-miR-484	26.066
hsa-miR-185-5p	30.22	hsa-miR-30c-5p	24.22	hsa-miR-31-3p	26.146
hsa-miR-107	30.26	hsa-miR-200b-3p	24.24	hsa-miR-335-5p	26.196
hsa-miR-132-3p	30.31	hsa-miR-199b-5p	24.26	hsa-miR-218-5p	26.206
hsa-miR-200a-3p	30.32	hsa-miR-28-3p	24.28	hsa-miR-132-3p	26.226
hsa-miR-210	30.33	hsa-miR-532-5p	24.44	hsa-miR-16-2-3p	26.246
hsa-miR-151a-3p	30.34	hsa-miR-200a-3p	24.5	hsa-miR-378a-5p	26.256
hsa-miR-10b-5p	30.36	hsa-miR-193b-3p	24.52	hsa-miR-455-3p	26.366
hsa-miR-425-5p	30.39	hsa-miR-324-5p	24.56	hsa-miR-181c-5p	26.446
hsa-miR-424-5p	30.4	hsa-miR-652-3p	24.57	hsa-miR-29c-5p	26.536
hsa-miR-335-3p	30.45	hsa-miR-151a-3p	24.65	hsa-miR-29a-5p	26.536
hsa-miR-532-5p	30.46	hsa-miR-144-3p	24.76	hsa-miR-10a-5p	26.546
hsa-miR-652-3p	30.46	hsa-let-7d-5p	24.76	hsa-miR-186-5p	26.636

hsa-miR-143-3p	30.47	hsa-miR-107	24.76	hsa-miR-34a-3p	26.666
hsa-miR-15b-5p	30.54	hsa-let-7f-5p	24.77	hsa-miR-374b-5p	26.676
hsa-miR-142-3p	30.55	hsa-miR-31-3p	24.8	hsa-miR-100-5p	26.686
hsa-miR-10a-5p	30.55	hsa-miR-191-5p	24.91	hsa-miR-93-3p	26.716
hsa-miR-484	30.66	hsa-miR-10a-5p	24.92	hsa-let-7b-3p	26.716
hsa-miR-146b-5p	30.73	hsa-miR-425-5p	24.93	hsa-miR-652-3p	26.736
hsa-miR-128	30.9	hsa-miR-551a	24.99	hsa-miR-328	26.766
hsa-miR-30d-5p	30.91	hsa-miR-31-5p	25.07	hsa-miR-660-5p	26.816
hsa-miR-139-5p	31.01	hsa-miR-452-5p	25.13	hsa-miR-107	26.816
hsa-miR-191-5p	31.1	hsa-miR-126-5p	25.17	hsa-miR-197-3p	26.876
hsa-miR-31-3p	31.15	hsa-miR-502-3p	25.19	hsa-miR-204-5p	26.886
hsa-miR-199a-5p	31.17	hsa-miR-135b-5p	25.23	hsa-miR-192-5p	26.936
hsa-miR-502-3p	31.17	hsa-miR-181b-5p	25.34	hsa-miR-505-3p	26.946
hsa-miR-145-5p	31.21	hsa-miR-132-3p	25.44	hsa-miR-143-3p	26.956
hsa-let-7b-3p	31.21	hsa-miR-30e-3p	25.49	hsa-miR-214-3p	27.016
hsa-miR-26b-5p	31.22	hsa-miR-30a-3p	25.62	hsa-miR-185-5p	27.036
hsa-miR-328	31.24	hsa-miR-146b-5p	25.64	hsa-miR-199b-5p	27.056
hsa-miR-632	31.3	hsa-miR-26b-5p	25.65	hsa-miR-200a-3p	27.156
hsa-miR-32-5p	31.38	hsa-miR-204-5p	25.66	hsa-miR-324-5p	27.186
hsa-miR-362-3p	31.38	hsa-miR-378a-5p	25.68	hsa-miR-125b-2-3p	27.256
hsa-miR-199b-5p	31.55	hsa-miR-708-5p	25.8	hsa-miR-99b-3p	27.286
hsa-miR-197-3p	31.55	hsa-miR-128	25.81	hsa-miR-421	27.306
hsa-miR-30a-3p	31.68	hsa-miR-200c-3p	25.81	hsa-miR-199a-5p	27.336
hsa-miR-452-5p	31.72	hsa-miR-214-3p	25.88	hsa-miR-214-5p	27.336
hsa-miR-144-3p	31.79	hsa-miR-10b-5p	25.89	hsa-miR-296-5p	27.446
hsa-miR-361-3p	31.81	hsa-miR-374b-5p	25.93	hsa-miR-425-5p	27.496
hsa-miR-551a	31.85	hsa-miR-876-5p	25.98	hsa-let-7f-1-3p	27.516
hsa-miR-142-5p	31.87	hsa-miR-16-2-3p	26.02	hsa-miR-106b-3p	27.596
hsa-miR-193b-3p	31.88	hsa-miR-192-5p	26.04	hsa-miR-2110	27.686
hsa-miR-93-3p	31.98	hsa-miR-224-3p	26.13	hsa-miR-501-3p	27.696
hsa-miR-455-3p	32.02	hsa-miR-15b-3p	26.14	hsa-miR-194-5p	27.716
hsa-miR-214-3p	32.03	hsa-miR-328	26.15	hsa-miR-598	27.776
hsa-miR-324-5p	32.04	hsa-miR-30d-5p	26.17	hsa-miR-769-5p	27.796
hsa-miR-505-3p	32.1	hsa-miR-142-3p	26.17	hsa-miR-18a-5p	27.836
hsa-miR-181b-5p	32.16	hsa-miR-34a-3p	26.19	hsa-miR-339-3p	27.856
hsa-miR-378a-5p	32.18	hsa-miR-598	26.2	hsa-miR-181c-3p	27.966
hsa-miR-30e-3p	32.19	hsa-miR-214-5p	26.23	hsa-miR-18b-5p	27.976
hsa-miR-590-5p	32.25	hsa-miR-181c-5p	26.24	hsa-miR-509-3p	27.976
hsa-miR-135b-5p	32.25	hsa-miR-145-5p	26.27	hsa-miR-215	28.026
hsa-miR-200c-3p	32.3	hsa-miR-514a-3p	26.28	hsa-miR-26b-5p	28.066
hsa-miR-876-5p	32.32	hsa-miR-193a-3p	26.28	hsa-let-7a-3p	28.176
hsa-miR-296-5p	32.43	hsa-let-7b-3p	26.28	hsa-miR-941	28.186
hsa-miR-224-3p	32.44	hsa-miR-363-3p	26.31	hsa-miR-15b-3p	28.196
hsa-miR-29c-5p	32.45	hsa-miR-141-3p	26.33	hsa-miR-181b-5p	28.216
hsa-miR-421	32.5	hsa-miR-32-5p	26.33	hsa-miR-338-3p	28.256
hsa-miR-363-3p	32.5	hsa-miR-455-3p	26.35	hsa-miR-28-5p	28.276
hsa-miR-598	32.52	hsa-miR-484	26.46	hsa-miR-16-5p	28.286
hsa-miR-15b-3p	32.54	hsa-miR-590-5p	26.52	hsa-miR-675-3p	28.286
hsa-miR-374b-5p	32.59	hsa-miR-29c-5p	26.52	hsa-miR-1271-5p	28.286
hsa-miR-214-5p	32.64	hsa-miR-203a	26.56	hsa-miR-148a-3p	28.296
hsa-miR-29a-5p	32.68	hsa-miR-376c-3p	26.61	hsa-miR-326	28.316
hsa-miR-485-3p	32.8	hsa-miR-29a-5p	26.64	hsa-miR-500a-5p	28.336

hsa-miR-16-2-3p	32.81	hsa-miR-505-3p	26.66	hsa-miR-363-3p	28.356
hsa-miR-181c-5p	32.86	hsa-miR-99a-3p	26.67	hsa-miR-139-5p	28.356
hsa-miR-501-3p	32.86	hsa-miR-127-3p	26.71	hsa-miR-141-3p	28.376
hsa-miR-2110	32.87	hsa-miR-509-3p	26.71	hsa-miR-30d-3p	28.426
hsa-miR-141-3p	32.99	hsa-miR-500a-5p	26.81	hsa-miR-221-5p	28.436
hsa-miR-708-5p	33	hsa-miR-513c-5p	26.82	hsa-miR-361-3p	28.446
hsa-miR-34a-3p	33.01	hsa-miR-142-5p	26.84	hsa-miR-376c-3p	28.546
hsa-miR-205-5p	33.04	hsa-miR-194-5p	26.9	hsa-miR-582-5p	28.546
hsa-miR-425-3p	33.04	hsa-miR-29b-2-5p	26.9	hsa-miR-142-5p	28.556
hsa-miR-582-5p	33.05	hsa-miR-28-5p	26.91	hsa-miR-146b-3p	28.556
hsa-miR-374a-5p	33.05	hsa-miR-205-5p	26.94	hsa-miR-26b-3p	28.586
hsa-miR-194-5p	33.06	hsa-miR-98-5p	26.96	hsa-miR-193a-3p	28.596
hsa-miR-509-3p	33.09	hsa-miR-224-5p	26.96	hsa-miR-205-5p	28.626
hsa-miR-192-5p	33.14	hsa-miR-421	26.98	hsa-miR-99a-3p	28.636
hsa-miR-33a-5p	33.21	hsa-miR-361-3p	27.02	hsa-miR-632	28.706
hsa-let-7f-1-3p	33.22	hsa-miR-96-5p	27.03	hsa-miR-30a-5p	28.736
hsa-miR-338-3p	33.23	hsa-miR-125b-2-3p	27.04	hsa-miR-485-3p	28.746
hsa-miR-376c-3p	33.26	hsa-miR-139-5p	27.24	hsa-miR-30e-3p	28.756
hsa-let-7e-3p	33.27	hsa-miR-215	27.26	hsa-miR-125a-3p	28.846
hsa-miR-28-5p	33.29	hsa-miR-18b-5p	27.27	hsa-miR-101-5p	28.846
hsa-miR-513c-5p	33.33	hsa-miR-296-5p	27.29	hsa-miR-342-5p	28.856
hsa-miR-92b-3p	33.41	hsa-miR-374a-5p	27.29	hsa-miR-224-5p	28.866
hsa-miR-224-5p	33.44	hsa-miR-339-5p	27.37	hsa-miR-887	28.876
hsa-miR-424-3p	33.44	hsa-miR-93-3p	27.37	hsa-miR-29b-2-5p	28.916
hsa-miR-98-5p	33.45	hsa-miR-2110	27.5	hsa-let-7f-2-3p	28.916
hsa-miR-106b-3p	33.47	hsa-miR-18a-5p	27.51	hsa-miR-590-5p	29.026
hsa-miR-29b-2-5p	33.48	hsa-miR-425-3p	27.54	hsa-miR-200b-5p	29.026
hsa-miR-203a	33.49	hsa-miR-326	27.57	hsa-miR-664a-3p	29.066
hsa-miR-744-5p	33.52	hsa-miR-106b-3p	27.57	hsa-miR-195-5p	29.106
hsa-miR-99a-3p	33.62	hsa-miR-154-5p	27.61	hsa-miR-133a	29.176
hsa-miR-339-5p	33.73	hsa-miR-501-3p	27.64	hsa-miR-452-5p	29.186
hsa-miR-339-3p	33.82	hsa-let-7f-1-3p	27.65	hsa-miR-144-3p	29.186
hsa-miR-215	33.83	hsa-miR-99b-3p	27.67	hsa-miR-744-5p	29.216
hsa-miR-769-5p	33.87	hsa-miR-339-3p	27.69	hsa-let-7i-3p	29.346
hsa-miR-664a-3p	33.88	hsa-miR-663a	27.9	hsa-miR-505-5p	29.356
hsa-miR-99b-3p	34.03	hsa-miR-376a-3p	27.93	hsa-miR-629-5p	29.406
hsa-miR-887	34.07	hsa-miR-744-5p	27.94	hsa-miR-345-5p	29.426
hsa-miR-454-3p	34.08	hsa-miR-582-5p	27.94	hsa-miR-501-5p	29.456
hsa-miR-500a-5p	34.12	hsa-miR-769-5p	27.96	hsa-miR-361-5p	29.486
hsa-miR-675-3p	34.12	hsa-miR-509-3-5p	28	hsa-miR-146b-5p	29.496
hsa-miR-34c-5p	34.16	hsa-miR-338-3p	28.02	hsa-miR-98-5p	29.536
hsa-miR-642a-5p	34.16	hsa-miR-101-5p	28.06	hsa-miR-425-3p	29.606
hsa-miR-125b-2-3p	34.2	hsa-miR-488-3p	28.18	hsa-miR-409-3p	29.696
hsa-miR-342-5p	34.24	hsa-miR-181c-3p	28.2	hsa-miR-181a-2-3p	29.756
hsa-let-7g-3p	34.25	hsa-let-7i-3p	28.2	hsa-miR-223-5p	29.766
hsa-miR-127-3p	34.31	hsa-miR-887	28.21	hsa-miR-24-1-5p	29.856
hsa-miR-181c-3p	34.34	hsa-let-7g-3p	28.22	hsa-miR-96-5p	29.866
hsa-miR-96-5p	34.36	hsa-miR-345-5p	28.25	hsa-miR-27b-5p	29.876
hsa-miR-34b-5p	34.36	hsa-miR-508-3p	28.28	hsa-miR-128	29.926
hsa-miR-488-3p	34.37	hsa-miR-21-3p	28.33	hsa-miR-605	29.966
hsa-miR-130b-3p	34.39	hsa-miR-382-5p	28.34	hsa-miR-144-5p	30.046

hsa-miR-18a-5p	34.4	hsa-miR-34c-5p	28.36	hsa-miR-663a	30.056
hsa-miR-149-5p	34.41	hsa-let-7a-3p	28.45	hsa-miR-489	30.076
hsa-miR-1271-5p	34.45	hsa-miR-429	28.51	hsa-miR-106b-5p	30.096
hsa-let-7a-3p	34.46	hsa-miR-197-3p	28.53	hsa-miR-125b-1-3p	30.156
hsa-miR-331-3p	34.49	hsa-miR-30d-3p	28.55	hsa-miR-188-3p	30.156
hsa-miR-181d	34.49	hsa-miR-17-5p	28.56	hsa-miR-32-5p	30.186
hsa-miR-17-5p	34.49	hsa-miR-495-3p	28.58	hsa-miR-17-5p	30.186
hsa-miR-125a-3p	34.53	hsa-miR-455-5p	28.59	hsa-miR-502-5p	30.196
hsa-miR-27b-5p	34.6	hsa-miR-144-5p	28.63	hsa-miR-145-3p	30.236
hsa-miR-7-1-3p	34.63	hsa-miR-135a-5p	28.64	hsa-miR-382-5p	30.256
hsa-miR-605	34.69	hsa-miR-629-5p	28.68	hsa-miR-130b-3p	30.316
hsa-miR-221-5p	34.69	hsa-miR-409-3p	28.72	hsa-miR-491-5p	30.326
hsa-miR-629-5p	34.76	hsa-miR-18a-3p	28.73	hsa-miR-135b-5p	30.376
hsa-miR-654-5p	34.78	hsa-miR-671-5p	28.77	hsa-miR-708-5p	30.376
hsa-miR-495-3p	34.8	hsa-miR-181d	28.79	hsa-miR-142-3p	30.396
hsa-miR-346	34.82	hsa-miR-130b-3p	28.83	hsa-miR-20a-3p	30.406
hsa-miR-514a-3p	34.83	hsa-miR-502-5p	28.83	hsa-miR-339-5p	30.526
hsa-miR-101-5p	34.83	hsa-miR-33a-5p	28.84	hsa-miR-127-3p	30.596
hsa-miR-200b-5p	34.85	hsa-miR-513a-3p	28.84	hsa-miR-10b-5p	30.606
hsa-miR-20a-3p	34.87	hsa-miR-377-3p	28.85	hsa-miR-511	30.616
hsa-miR-345-5p	34.89	hsa-miR-1271-5p	28.9	hsa-miR-642a-5p	30.636
hsa-miR-663a	34.9	hsa-miR-454-3p	28.93	hsa-miR-181d	30.656
hsa-miR-30d-3p	34.97	hsa-miR-146b-3p	28.94	hsa-miR-181a-3p	30.686
hsa-miR-651	34.98	hsa-miR-342-5p	28.95	hsa-miR-30d-5p	30.716
hsa-miR-409-3p	34.98	hsa-let-7f-2-3p	28.95	hsa-miR-203a	30.736
hsa-miR-154-5p	35.03	hsa-miR-92b-3p	28.96	hsa-miR-154-5p	30.736
hsa-miR-508-3p	35.04	hsa-miR-26b-3p	28.98	hsa-miR-17-3p	30.736
hsa-miR-429	35.07	hsa-miR-221-5p	29	hsa-miR-132-5p	30.746
hsa-miR-26b-3p	35.07	hsa-miR-642a-5p	29.01	hsa-miR-562	30.776
hsa-miR-24-1-5p	35.07	hsa-miR-424-3p	29.04	hsa-miR-149-5p	30.816
hsa-miR-628-3p	35.08	hsa-miR-34b-5p	29.05	hsa-miR-624-5p	30.816
hsa-miR-144-5p	35.09	hsa-miR-181a-2-3p	29.12	hsa-miR-193b-5p	30.846
hsa-miR-223-5p	35.14	hsa-miR-125a-3p	29.14	hsa-miR-296-3p	30.846
hsa-miR-491-5p	35.18	hsa-miR-7-1-3p	29.18	hsa-miR-24-2-5p	30.916
hsa-miR-18b-5p	35.21	hsa-miR-941	29.19	hsa-miR-23b-5p	30.956
hsa-miR-326	35.27	hsa-miR-200b-5p	29.22	hsa-miR-486-3p	30.966
hsa-miR-21-3p	35.3	hsa-miR-651	29.24	hsa-miR-30e-5p	30.986
hsa-miR-382-5p	35.31	hsa-miR-23b-5p	29.24	hsa-miR-643	31.036
hsa-miR-329	35.32	hsa-miR-188-3p	29.25	hsa-miR-133b	31.066
hsa-miR-501-5p	35.32	hsa-miR-331-3p	29.31	hsa-miR-584-5p	31.086
hsa-miR-511	35.37	hsa-miR-183-5p	29.38	hsa-miR-1908	31.096
hsa-miR-377-3p	35.39	hsa-miR-145-3p	29.5	hsa-miR-130b-5p	31.156
hsa-miR-376a-3p	35.4	hsa-miR-505-5p	29.51	hsa-miR-34b-5p	31.156
hsa-miR-133b	35.42	hsa-miR-511	29.54	hsa-miR-134	31.166
hsa-miR-510	35.47	hsa-miR-503-5p	29.55	hsa-miR-219-5p	31.236
hsa-miR-146b-3p	35.49	hsa-miR-491-5p	29.55	hsa-miR-638	31.256
hsa-miR-18a-3p	35.52	hsa-miR-411-5p	29.66	hsa-miR-504	31.266
hsa-miR-502-5p	35.54	hsa-miR-133a	29.74	hsa-miR-346	31.296
hsa-miR-671-5p	35.6	hsa-miR-133b	29.76	hsa-miR-629-3p	31.386
hsa-miR-582-3p	35.61	hsa-miR-501-5p	29.76	hsa-miR-654-3p	31.396
hsa-miR-206	35.62	hsa-miR-1	29.76	hsa-miR-1247-5p	31.426
hsa-miR-362-5p	35.68	hsa-miR-181a-3p	29.83	hsa-miR-190a	31.486

hsa-miR-190b	35.69	hsa-miR-24-1-5p	29.84	hsa-miR-509-3-5p	31.506
hsa-miR-489	35.73	hsa-miR-584-5p	29.85	hsa-miR-1537	31.516
hsa-let-7a-2-3p	35.73	hsa-miR-506-3p	29.86	hsa-miR-942	31.526
hsa-miR-196b-5p	35.74	hsa-miR-149-5p	29.91	hsa-miR-135b-3p	31.526
hsa-miR-584-5p	35.75	hsa-miR-34c-3p	29.95	hsa-miR-183-5p	31.556
hsa-miR-125b-1-3p	35.75	hsa-miR-138-5p	29.96	hsa-miR-1468	31.576
hsa-miR-217	35.85	hsa-miR-664a-3p	29.98	hsa-miR-429	31.626
hsa-miR-570-3p	35.88	hsa-miR-27b-5p	30.01	hsa-miR-196b-5p	31.636
hsa-miR-17-3p	35.88	hsa-let-7e-3p	30.08	hsa-miR-7-1-3p	31.666
hsa-miR-212-3p	35.91	hsa-miR-628-3p	30.11	hsa-miR-92b-5p	31.676
hsa-let-7f-2-3p	36.05	hsa-miR-155-5p	30.14	hsa-miR-1270	31.736
hsa-miR-135a-5p	36.06	hsa-miR-125b-1-3p	30.14	hsa-miR-582-3p	31.786
hsa-miR-942	36.08	hsa-miR-193b-5p	30.14	hsa-miR-940	31.806
hsa-let-7i-3p	36.08	hsa-miR-675-3p	30.15	hsa-miR-513c-5p	31.816
hsa-miR-155-5p	36.11	hsa-miR-632	30.21	hsa-miR-155-5p	31.826
hsa-miR-23b-5p	36.17	hsa-miR-187-3p	30.22	hsa-miR-513a-3p	31.836
hsa-miR-145-3p	36.17	hsa-miR-188-5p	30.28	hsa-miR-365b-5p	31.846
hsa-miR-454-5p	36.23	hsa-miR-337-5p	30.29	hsa-miR-330-3p	31.956
hsa-miR-503-5p	36.25	hsa-miR-624-5p	30.33	hsa-miR-671-3p	31.956
hsa-miR-187-3p	36.25	hsa-miR-219-5p	30.44	hsa-miR-548c-5p	32.056
hsa-miR-188-5p	36.26	hsa-miR-24-2-5p	30.45	hsa-miR-514a-3p	32.216
hsa-miR-130b-5p	36.26	hsa-miR-346	30.51	hsa-miR-212-3p	32.226
hsa-miR-1468	36.27	hsa-miR-510	30.54	hsa-let-7a-2-3p	32.276
hsa-miR-520h	36.3	hsa-miR-376b-3p	30.55	hsa-miR-10a-3p	32.306
hsa-miR-548c-5p	36.34	hsa-miR-20a-3p	30.56	hsa-miR-503-5p	32.396
hsa-miR-934	36.39	hsa-miR-410	30.63	hsa-miR-33a-5p	32.456
hsa-miR-638	36.41	hsa-miR-362-5p	30.64	hsa-miR-331-5p	32.466
hsa-miR-183-5p	36.44	hsa-miR-330-3p	30.68	hsa-let-7e-3p	32.496
hsa-miR-193a-3p	36.44	hsa-miR-489	30.68	hsa-miR-579	32.506
hsa-miR-188-3p	36.44	hsa-miR-10a-3p	30.68	hsa-miR-25-5p	32.526
hsa-miR-34c-3p	36.49	hsa-miR-940	30.71	hsa-miR-21-3p	32.536
hsa-miR-629-3p	36.49	hsa-miR-485-3p	30.71	hsa-miR-340-5p	32.536
hsa-miR-323a-3p	36.51	hsa-miR-17-3p	30.75	hsa-let-7f-5p	32.556
hsa-miR-493-3p	36.53	hsa-miR-132-5p	30.83	hsa-miR-92b-3p	32.586
hsa-miR-941	36.53	hsa-miR-654-3p	30.99	hsa-miR-628-3p	32.616
hsa-miR-493-3p	36.53	hsa-miR-329	31.03	hsa-miR-337-5p	32.646
hsa-miR-365b-5p	36.54	hsa-miR-134	31.03	hsa-miR-410	32.816
hsa-miR-625-3p	36.56	hsa-miR-323a-3p	31.19	hsa-miR-510	32.816
hsa-miR-671-3p	36.59	hsa-miR-223-5p	31.21	hsa-miR-495-3p	32.826
hsa-miR-548b-3p	36.59	hsa-miR-487b	31.22	hsa-miR-362-5p	32.856
hsa-miR-486-3p	36.61	hsa-miR-942	31.26	hsa-miR-33b-5p	32.856
hsa-miR-643	36.63	hsa-miR-1537	31.27	hsa-miR-34c-5p	32.866
hsa-miR-455-5p	36.65	hsa-miR-212-3p	31.28	hsa-miR-187-3p	32.886
hsa-miR-509-3-5p	36.66	hsa-miR-296-3p	31.33	hsa-let-7g-3p	32.886
hsa-miR-296-3p	36.74	hsa-miR-30c-2-3p	31.36	hsa-miR-190b	32.916
hsa-miR-576-5p	36.76	hsa-miR-627	31.37	hsa-miR-376a-3p	32.976
hsa-miR-615-3p	36.79	hsa-miR-629-3p	31.38	hsa-miR-454-5p	32.996
hsa-miR-331-5p	36.94	hsa-miR-340-5p	31.41	hsa-miR-455-5p	33.016
hsa-miR-627	36.95	hsa-miR-1468	31.7	hsa-miR-708-3p	33.076
hsa-miR-506-3p	36.97	hsa-miR-1247-5p	31.73	hsa-miR-377-3p	33.116
hsa-miR-1247-5p	37.03	hsa-miR-190a	31.77	hsa-miR-34c-3p	33.176

hsa-miR-940	37.05	hsa-miR-20b-5p	31.81	hsa-miR-616-5p	33.176
hsa-miR-20b-5p	37.06	hsa-miR-33b-5p	31.82	hsa-miR-411-5p	33.216
hsa-miR-1537	37.06	hsa-miR-548b-3p	31.88	hsa-miR-508-5p	33.326
hsa-miR-1908	37.08	hsa-miR-365b-5p	31.89	hsa-miR-20b-5p	33.336
hsa-miR-134	37.11	hsa-miR-26a-2-3p	31.9	hsa-miR-589-3p	33.496
hsa-miR-190a	37.15	hsa-miR-643	31.9	hsa-miR-493-3p	33.556
hsa-miR-616-5p	37.15	hsa-miR-1908	31.9	hsa-miR-493-3p	33.556
hsa-miR-579	37.15	hsa-miR-25-5p	31.93	hsa-miR-1207-5p	33.566
hsa-miR-330-3p	37.3	hsa-miR-671-3p	31.94	hsa-miR-323a-3p	33.666
hsa-miR-133a	37.3	hsa-miR-135b-3p	31.98	hsa-miR-625-3p	33.746
hsa-miR-1914-5p	37.35	hsa-miR-579	32.02	hsa-miR-135a-5p	33.746
hsa-miR-182-3p	37.39	hsa-miR-570-3p	32.07	hsa-miR-627	33.786
hsa-miR-92b-5p	37.39	hsa-miR-454-5p	32.08	hsa-miR-18a-3p	33.786
hsa-miR-337-5p	37.42	hsa-miR-1270	32.08	hsa-miR-338-5p	33.806
hsa-miR-411-5p	37.48	hsa-miR-582-3p	32.1	hsa-miR-188-5p	33.986
hsa-miR-450a-5p	37.51	hsa-miR-130b-5p	32.14	hsa-miR-576-5p	33.986
hsa-miR-545-3p	37.58	hsa-miR-655	32.15	hsa-miR-1538	34.026
hsa-miR-550a-5p	37.69	hsa-miR-616-5p	32.15	hsa-miR-628-5p	34.076
hsa-miR-181a-3p	37.7	hsa-miR-548c-5p	32.16	hsa-miR-506-3p	34.216
hsa-miR-340-5p	37.74	hsa-miR-23a-5p	32.2	hsa-miR-124-3p	34.236
hsa-miR-25-5p	37.75	hsa-miR-708-3p	32.22	hsa-miR-138-5p	34.256
hsa-miR-10a-3p	37.76	hsa-miR-216a-5p	32.28	hsa-miR-487b	34.276
hsa-miR-1255b-5p	37.86	hsa-miR-576-5p	32.3	hsa-miR-654-5p	34.396
hsa-miR-193b-5p	37.86	hsa-miR-562	32.31	hsa-miR-548b-3p	34.456
hsa-miR-1207-5p	37.92	hsa-miR-504	32.32	hsa-miR-508-3p	34.466
hsa-miR-135b-3p	37.93	hsa-let-7a-2-3p	32.54	hsa-miR-23a-5p	34.476
hsa-miR-562	37.95	hsa-miR-628-5p	32.59	hsa-miR-1	34.556
hsa-miR-138-5p	37.98	hsa-miR-545-3p	32.62	hsa-miR-216a-5p	34.566
hsa-miR-219-5p	37.99	hsa-miR-486-3p	32.65	hsa-miR-1914-5p	34.626
hsa-miR-26a-2-3p	37.99	hsa-miR-654-5p	32.68	hsa-miR-550a-5p	34.636
hsa-miR-181a-2-3p	38.15	hsa-miR-217	32.73	hsa-miR-217	34.676
hsa-miR-504	38.18	hsa-miR-182-3p	32.8	hsa-miR-182-3p	34.696
hsa-miR-1538	38.18	hsa-miR-331-5p	32.82	hsa-miR-362-3p	34.796
hsa-miR-665	38.22	hsa-miR-493-3p	32.83	hsa-miR-615-3p	34.916
hsa-miR-624-5p	38.23	hsa-miR-493-3p	32.83	hsa-miR-376b-3p	34.936
hsa-miR-487b	38.25	hsa-miR-625-3p	32.87	hsa-let-7a-5p	34.946
hsa-miR-628-5p	38.25	hsa-miR-92b-5p	32.94	hsa-miR-876-5p	35.016
hsa-miR-654-3p	38.26	hsa-miR-638	33.01	hsa-miR-520h	35.156
hsa-miR-708-3p	38.28	hsa-miR-508-5p	33.05	hsa-miR-26a-2-3p	35.386
hsa-miR-376b-3p	38.31	hsa-miR-206	33.3	hsa-miR-545-3p	35.416
hsa-miR-23a-5p	38.42	hsa-miR-605	33.32	hsa-miR-330-5p	35.456
hsa-miR-24-2-5p	38.44	hsa-miR-1207-5p	33.34	hsa-miR-155-3p	35.486
hsa-miR-1249	38.51	hsa-miR-190b	33.71	hsa-miR-655	35.556
hsa-miR-30c-2-3p	38.58	hsa-miR-589-3p	33.89	hsa-miR-934	35.596
hsa-miR-1	38.6	hsa-miR-550a-5p	33.95	hsa-miR-331-3p	35.686
hsa-miR-513a-3p	38.64	hsa-miR-124-3p	34.05	hsa-miR-1249	35.896
hsa-miR-589-3p	38.65	hsa-miR-330-5p	34.05	hsa-miR-424-3p	35.966
hsa-miR-410	38.75	hsa-miR-615-3p	34.17	hsa-miR-206	36.026
hsa-miR-508-5p	38.77	hsa-miR-196b-5p	34.2	hsa-miR-651	36.126
hsa-miR-330-5p	38.84	hsa-miR-450a-5p	34.59	hsa-miR-1471	36.186

hsa-miR-655	38.94	hsa-miR-1255b-5p	34.64	hsa-miR-1255b-5p	36.426
hsa-miR-1471	39.03	hsa-miR-665	34.67	hsa-miR-570-3p	36.446
hsa-miR-216a-5p	39.33	hsa-miR-1538	35.74	hsa-miR-671-5p	36.586
hsa-miR-124-3p	39.47	hsa-miR-338-5p	35.97	hsa-miR-335-3p	37.236
hsa-miR-155-3p	39.54	hsa-miR-520h	36.52	hsa-miR-665	37.416
hsa-miR-338-5p	39.57	hsa-miR-1914-5p	36.67	hsa-miR-329	37.686
hsa-miR-33b-5p	39.59	hsa-miR-1249	36.92	hsa-miR-30c-2-3p	37.696
hsa-miR-1270	39.85	hsa-miR-934	36.98	hsa-miR-450a-5p	37.806
hsa-miR-505-5p	39.89	hsa-miR-155-3p	37.13	hsa-miR-488-3p	37.866
hsa-miR-132-5p	39.97	hsa-miR-1471	39.24	hsa-miR-454-3p	37.956

Table S3. Particulars of the 16 cardiovascular microRNAs that were selected for further investigation

miRNAs	Previous IDs	Assay ID code	Position in PF sample			Previously reported expressed in these cardiovascular tissues/cells or progenitor cells	Reported cardiovascular functions	Reference
			1	2	3			
let-7b-5p	let-7b	000378	8	12	15	Human mesenchymal stem cells (MSCs)	Let-7b-overexpressing MSCs promote heart regeneration in rats	1
miR-15a-5p	miR-15a	000389	16	13	9	Rat myocardium and cardiomyocytes<br>Human vascular smooth muscle, endothelial cells and bone marrow–derived proangiogenic cells	Induces cardiomyocyte apoptosis<br>Anti-angiogenic<br>Anti-proliferative for vascular cells	2-4
miR-16-5p	miR-16	000391	1	2	140	Rat myocardium and cardiomyocytes<br>Human bone marrow–derived proangiogenic cells	Reduces cardiomyocyte hypertrophy<br>Anti-angiogenic	3,5
miR-19b-3p	miR-19b	000396	10	11	5	Rat cardiomyocytes	Induces cardiomyocyte hypertrophy<br>Anti-apoptotic<br>Anti-angiogenic	6
miR-21-5p	miR-21	000397	2	3	2	Rodent cardiomyocytes and cardiac fibroblast,<br>Human Endothelial cells	Anti-apoptotic<br>Anti-angiogenic<br>Proangiogenic in the chicken chorioallantoic membrane assay and in cancer	7-10
miR-22-3p	miR-22	000398	15	9	8	Rat myocardium and cardiomyocytes	Induces cardiomyocyte hypertrophy	11
miR-23a-3p	miR-23a	000399	5	10	10	Rodent heart and cardiomyocytes	Induces cardiomyocyte hypertrophy	12,13

miR-24-3p	miR-24	000402	6	5	4	Mouse heart, cardiomyocytes, cardiac fibroblasts and myocardial endothelial cells<br>Human endothelial cells<br>Mouse and human aorta	Inhibits cardiomyocytes apoptosis<br>Anti-fibrotic<br>Anti-angiogenic<br>Limits aneurysm evolution	12,14-17
miR-27a-3p	miR-27a	000408	40	28	21	Rat cardiomyocytes Human endothelial cell	Reduces cardiomyocyte hypertrophy<br>Pro-angiogenic	6,11,18
miR-27b-3p	miR-27b	000409	7	8	16	Rodent heart and cardiomyocytes<br>Human endothelial cells	Induces cardiomyocyte hypertrophy<br>Pro-angiogenic	19
miR-29a-3p	miR-29a	002112	9	6	6	Rat cardiac myocytes (immortalized H9c2 cell line)<br>Human endothelial cells<br>Murine endothelial cells	Inhibits cardiomyocyte proliferation<br>Pro-angiogenic	20,21
miR-29b-3p	miR-29b	000413	51	16	19	Rodent heart and cardiac fibroblasts	Antifibrotic	22,23
miR-29c-3p	miR-29c	000587	11	7	11	Human endothelial cells	Anti-angiogenic	24
miR-126-3p	miR-126	002228	59	71	59	Mouse and human endothelial cells	Pro-angiogenic	25,26
miR-208a	miR-208	000511	N.D	N.D.	N.D.	Rat, murine and human myocardium	Induces cardiomyocyte hypertrophy<br>Pro-fibrotic	27-29
miR-451a	miR-451	001141	3	1	1	Rat cardiac myocytes (immortalized H9c2 cell line)<br>Mouse heart	Anti-apoptotic	30,31

N.D.: Not detected

Additional References for Table S3

1. *Ham, O, Lee, S-Y, Lee, CY, Park, J-H, Lee, J, Seo, H-H, et al.* (2015). let-7b suppresses apoptosis and autophagy of human mesenchymal stem cells transplanted into ischemia/reperfusion injured heart 7by targeting caspase-3. *Stem Cell Res Ther* 6: 147.
2. Brittan, M, Hunter, A, Boulberdaa, M, Fujisawa, T, Skinner, EM, Shah, AS, *et al.* (2015). Impaired vascular function and repair in patients with premature coronary artery disease. *Eur J Prev Cardiol* 22: 1557–1566.
3. Liu, L, Zhang, G, Liang, Z, Liu, X, Li, T, Fan, J, *et al.* (2014). MicroRNA-15b enhances hypoxia/reoxygenation-induced apoptosis of cardiomyocytes via a mitochondrial apoptotic pathway. *Apoptosis* 19: 19–29.
4. Spinetti, G, Fortunato, O, Caporali, A, Shantikumar, S, Marchetti, M, Meloni, M, *et al.* (2013). MicroRNA-15a and microRNA-16 impair human circulating proangiogenic cell functions and are increased in the proangiogenic cells and serum of patients with critical limb ischemia. *Circ. Res.* 112: 335–346.
5. Zheng, X, Li, A, Zhao, L, Zhou, T, Shen, Q, Cui, Q, *et al.* (2013). Key role of microRNA-15a in the KLF4 suppressions of proliferation and angiogenesis in endothelial and vascular smooth muscle cells. *Biochem. Biophys. Res. Commun.* 437: 625–631.
6. Huang, S, Zou, X, Zhu, J-N, Fu, Y-H, Lin, Q-X, Liang, Y-Y, *et al.* (2015). Attenuation of microRNA-16 derepresses the cyclins D1, D2 and E1 to provoke cardiomyocyte hypertrophy. *J. Cell. Mol. Med.* 19: 608–619.
7. Song, DW, Ryu, JY, Kim, JO, Kwon, EJ and Kim, DH (2014). The miR-19a/b family positively regulates cardiomyocyte hypertrophy by targeting atrogin-1 and MuRF-1. *Biochem. J.* 457: 151–162.
8. Thum, T, Gross, C, Fiedler, J, Fischer, T, Kissler, S, Bussen, M, *et al.* (2008). MicroRNA-21 contributes to myocardial disease by stimulating MAP kinase signalling in fibroblasts. *Nature* 456: 980–984.
9. Cheng, Y, Zhu, P, Yang, J, Liu, X, Dong, S, Wang, X, *et al.* (2010). Ischaemic preconditioning-regulated miR-21 protects heart against ischaemia/reperfusion injury via anti-apoptosis through its target PDCD4. *Cardiovasc. Res.* 87: 431–439.
10. Liu, L-Z, Li, C, Chen, Q, Jing, Y, Carpenter, R, Jiang, Y, *et al.* (2011). MiR-21 induced angiogenesis through AKT and ERK activation and HIF-1α expression. *PLoS ONE* 6: e19139.
11. Sabatel, C, Malvaux, L, Bovy, N, Deroanne, C, Lambert, V, Gonzalez, M-LA, *et al.* (2011). MicroRNA-21 exhibits antiangiogenic function by targeting RhoB expression in endothelial cells. *PLoS ONE* 6: e16979.
12. Jentzsch, C, Leierseder, S, Loyer, X, Flohrschütz, I, Sassi, Y, Hartmann, D, *et al.* (2012). A phenotypic screen to identify hypertrophy-modulating microRNAs in primary cardiomyocytes. *J. Mol. Cell. Cardiol.* 52: 13–20.
13. Xu, X-D, Song, X-W, Li, Q, Wang, G-K, Jing, Q and Qin, Y-W (2012). Attenuation of microRNA-22 derepressed PTEN to effectively protect rat cardiomyocytes from hypertrophy. *J. Cell. Physiol.* 227: 1391–1398.
14. Qian, L, Van Laake, LW, Huang, Y, Liu, S, Wendland, MF and Srivastava, D (2011). miR-24 inhibits apoptosis and represses Bim in mouse cardiomyocytes. *J. Exp. Med.* 208: 549–560.
15. Lin, Z, Murtaza, I, Wang, K, Jiao, J, Gao, J and Li, P-F (2009). miR-23a functions downstream of NFATc3 to regulate cardiac hypertrophy. *Proc. Natl. Acad. Sci. U.S.A.* 106: 12103–12108.
16. Wang, K, Lin, Z-Q, Long, B, Li, J-H, Zhou, J and Li, P-F (2012). Cardiac hypertrophy is positively regulated by MicroRNA miR-23a. *J. Biol. Chem.* 287: 589–599.
17. Wang, J, Huang, W, Xu, R, Nie, Y, Cao, X, Meng, J, *et al.* (2012). MicroRNA-24 regulates cardiac fibrosis after myocardial infarction. *J. Cell. Mol. Med.* 16: 2150–2160.
18. Fiedler, J, Jazbutyte, V, Kirchmaier, BC, Gupta, SK, Lorenzen, J, Hartmann, D, *et al.* (2011). MicroRNA-24 regulates vascularity after myocardial infarction. *Circulation* 124: 720–730.
19. Meloni, M, Marchetti, M, Garner, K, Littlejohns, B, Sala-Newby, G, Xenophontos, N, *et al.* (2013). Local inhibition of microRNA-24 improves reparative angiogenesis and left ventricle remodeling and function in mice with myocardial infarction. *Mol. Ther.* 21: 1390–1402.
20. Maegdefessel, L, Spin, JM, Raaz, U, Eken, SM, Toh, R, Azuma, J, *et al.* (2014). miR-24 limits aortic vascular inflammation and murine abdominal aneurysm development. *Nat Commun* 5: 5214.
21. Urbich, C, Kaluza, D, Frömel, T, Knau, A, Bennewitz, K, Boon, RA, *et al.* (2012). MicroRNA-27a/b controls endothelial cell repulsion and angiogenesis by targeting semaphorin 6A. *Blood* 119: 1607–1616.
22. Cao, X, Wang, J, Wang, Z, Du, J, Yuan, X, Huang, W, *et al.* (2013). MicroRNA profiling

during rat ventricular maturation: A role for miR-29a in regulating cardiomyocyte cell cycle re-entry. *FEBS Lett.* 587: 1548–1555.
23. Yang, Z, Wu, L, Zhu, X, Xu, J, Jin, R, Li, G, *et al.* (2013). MiR-29a modulates the angiogenic properties of human endothelial cells. *Biochem. Biophys. Res. Commun.* 434: 143–149.
24. Abonnenc, M, Nabeebaccus, AA, Mayr, U, Barallobre-Barreiro, J, Dong, X, Cuello, F, *et al.* (2013). Extracellular matrix secretion by cardiac fibroblasts: role of microRNA-29b and microRNA-30c. *Circ. Res.* 113: 1138–1147.
25. Zhu, J-N, Chen, R, Fu, Y-H, Lin, Q-X, Huang, S, Guo, L-L, *et al.* (2013). Smad3 inactivation and MiR-29b upregulation mediate the effect of carvedilol on attenuating the acute myocardium infarction-induced myocardial fibrosis in rat. *PLoS ONE* 8: e75557.
26. Hu, Y, Deng, F, Song, J, Lin, J, Li, X, Tang, Y, *et al.* (2015). Evaluation of miR-29c inhibits endotheliocyte migration and angiogenesis of human endothelial cells by suppressing the insulin like growth factor 1. *Am J Transl Res* 7: 489–501.
27. Wang, S, Aurora, AB, Johnson, BA, Qi, X, McAnally, J, Hill, JA, *et al.* (2008). The endothelial-specific microRNA miR-126 governs vascular integrity and angiogenesis. *Dev. Cell* 15: 261–271.
28. Fish, JE, Santoro, MM, Morton, SU, Yu, S, Yeh, R-F, Wythe, JD, *et al.* (2008). miR-126 regulates angiogenic signaling and vascular integrity. *Dev. Cell* 15: 272–284.
29. van Solingen, C, Seghers, L, Bijkerk, R, Duijs, JMGJ, Roeten, MK, van Oeveren-Rietdijk, AM, *et al.* (2009). Antagomir-mediated silencing of endothelial cell specific microRNA-126 impairs ischemia-induced angiogenesis. *J. Cell. Mol. Med.* 13: 1577–1585.
30. Montgomery, RL, Hullinger, TG, Semus, HM, Dickinson, BA, Seto, AG, Lynch, JM, *et al.* (2011). Therapeutic inhibition of miR-208a improves cardiac function and survival during heart failure. *Circulation* 124: 1537–1547.
31. van Rooij, E, Sutherland, LB, Qi, X, Richardson, JA, Hill, J and Olson, EN (2007). Control of stress-dependent cardiac growth and gene expression by a microRNA. *Science* 316: 575–579.
32. Callis, TE, Pandya, K, Seok, HY, Tang, R-H, Tatsuguchi, M, Huang, Z-P, *et al.* (2009). MicroRNA-208a is a regulator of cardiac hypertrophy and conduction in mice. *J. Clin. Invest.* 119: 2772–2786.
33. Zhang, Y, Wang, Y, Wang, XK and Zeng, C (2011). Insulin promotes vascular smooth muscle cell proliferation via microrna-208 mediated down-regulation of p21. *Journal of Hypertension* 29: e427.
34. Zhang, X, Wang, X, Zhu, H, Zhu, C, Wang, Y, Pu, WT, *et al.* (2010). Synergistic effects of the GATA-4-mediated miR-144/451 cluster in protection against simulated ischemia/reperfusion-induced cardiomyocyte death. *J. Mol. Cell. Cardiol.* 49: 841–850.
35. Wang, X, Zhu, H, Zhang, X, Liu, Y, Chen, J, Medvedovic, M, *et al.* (2012). Loss of the miR-144/451 cluster impairs ischaemic preconditioning-mediated cardioprotection by targeting Rac-1. *Cardiovasc. Res.* 94: 379–390.

Supplemental raw data

CT from the whole PF qPCR analyses on the selective 16 putative cardiovascular miRNAs used to validate the Exiqon array
(The CT value in the Exiqon array are shown for comparison)

	miR-19b	
	qPCR CT	Array Ct
	28.825	26.04
	25.675	20.04
	26.46	20.466
	27.25	/
	31.255	/
**Average**	27.893	22.182
**St Dev**	2.211228731	3.347908601
**CV**	0.0792754	0.150929069

	miR-27b	
	qPCR CT	Array Ct
	28.885	25.41
	29.41	19.55
	30.19	21.606
	35	/
	31.505	/
**Average**	30.998	22.18866667
**St Dev**	2.445016871	2.973133925
**CV**	0.078876601	0.133993357

	miR-29c	
	qPCR CT	Array Ct
	27.405	26.06
	25.46	19.52
	27.015	21.186
	25.525	/
	30.75	/
**Average**	27.231	22.25533333
**St Dev**	2.150998024	3.398603439
**CV**	0.078990783	0.152709617

	Let-7b	
	qPCR CT	Array Ct
	25.635	25.54
	23.255	20.06
	23.1	21.476
	25.2	/
	25.01	/
**Average**	24.44	22.35866667
**St Dev**	1.17583481	2.844630966
**CV**	0.048111081	0.127227218

	miR-22	
	qPCR CT	Array Ct
	31.42	26.39
	26.18	19.62
	27.24	20.926
	30.135	/
	31.99	/
**Average**	29.393	22.312
**St Dev**	2.567220287	3.591513887
**CV**	0.087341213	0.160967815

	miR-15a	
	qPCR CT	Array Ct
	35	26.44
	34.275	20.31
	34.345	21.036
	35	/
	35	/
**Average**	34.724	22.59533333
**St Dev**	0.378738036	3.349308187
**CV**	0.010907097	0.148230085

	miR-208	
	qPCR CT	Array Ct
	n.d.	*n.d.*
	22.49	*n.d.*
	23.395	*n.d.*
	23.415	/
	27.35	/
**Average**	24.1625	/
**St Dev**	2.168349987	/
**CV**	0.0897403	/

	miR-16	
	qPCR CT	Array Ct
	26.73	23.12
	22.49	17
	23.395	28.286
	23.415	/
	27.35	/
**Average**	24.676	22.802
**St Dev**	2.201071898	5.649716099
**CV**	0.089198894	0.247772831

	miR-21-5p	
	qPCR CT	Array Ct
	23.835	23.19
	21.823	17.08
	29.715	20.256
	27.288	
	28.245	
	/	
	19.545	
	21.168	
	23.416	
**Average**	24.379	20.17533333
**St Dev**	3.651116522	3.055798641
**CV**	0.149762928	0.151462114

	miR-23a-3p	
	qPCR CT	Array Ct
	28.186	24.82
	28.309	19.66
	30.873	21.176
	30.729	
	30.845	
	26.733	
	27.178	
	28.403	
	26.803	
**Average**	28.673	21.88533333
**St Dev**	1.722113082	2.652124683
**CV**	0.060059838	0.121182741

	miR-24-3p	
	qPCR CT	Array Ct
	24.431	25.08
	22.045	19.12
	27.951	20.386
	27.256	
	27.984	
	21.858	
	21.899	
	19.854	
	22.560	
**Average**	23.982	21.52866667
**St Dev**	3.048448074	3.140010403
**CV**	0.12711311	0.145852525

	miR-27a-3p	
	qPCR CT	Array Ct
	28.632	28.03
	27.173	21.79
		22.396
	32.802	
	30.25076365	
	26.28535959	
	33.70680682	
	22.030	
	24.566	
**Average**	28.181	24.072
**St Dev**	3.99786915	3.441094593
**CV**	0.141865501	0.142950091

	miR-29a-3p	
	qPCR CT	Array Ct
	22.74752856	25.86
	18.75318504	19.28
	18.64544536	20.656
	18.44289977	
	24.56168632	
	25.1331412	
	20.28724242	
	18.634	
	21.643	
**Average**	20.983	21.932
**St Dev**	2.656806664	3.470624151
**CV**	0.126616253	0.158244763

	miR-29b-3p	
	qPCR CT	Array Ct
	32.32929513	28.45
	30.68970959	20.72
	33.63064721	22.286
	35.4524306	
	34.59613255	
	30.55840402	
	36.1804272	
	27.59420613	
	29.40250852	
**Average**	32.270	23.81866667
**St Dev**	2.91970691	4.086566448
**CV**	0.090476266	0.171569908

	miR-126-3p	
	qPCR CT	Array Ct
	28.41164267	28.83
	26.28378965	23.86
	33.209562	25.416
	36.01387719	
	27.83337422	
	32.64411207	
	29.27828862	
	33.40568267	
	/	
**Average**	30.885	26.03533333
**St Dev**	3.387790275	2.542224485
**CV**	0.109690327	0.097645168

	miR-451	
	qPCR CT	Array Ct
	23.105	23.4
	21.33	16.49
	21.795	16.606
	21.8	/
	25.595	/
**Average**	22.725	18.832
**St Dev**	1.735500648	3.956429198
**CV**	0.076369665	0.21009076

Raw data of the paragraph: *Human PF is enriched with miRNAs of potential cardiovascular origin.*

Raw data are provided for the 3 miRs that are not shown in Figure 1.

	miR-19b-3p	
**Samples**	**Relative Expression PF**	**Relative Expression PLASMA**
1	1.97E-03	2.60E-02
2	5.08E-03	1.55E-02
3	8.64E-03	6.62E-03
4	1.81E-03	1.05E-02
5	6.47E-04	9.38E-05

	miR-15a-3p	
**Samples**	**Relative Expression PF**	**Relative Expression PLASMA**
1	7.540E-05	4.7139E-01
2	6.915E-05	6.1854E-02
3	1.844E-04	4.2484E-01
4	7.309E-05	2.2144E-01
5	2.709E-04	8.8388E-02

	miR-126-3p	
**Samples**	**Relative Expression PF**	**Relative Expression PLASMA**
1	5.0000E-01	7.0711E-01
2	2.5000E-01	8.4090E-01
3	1.2500E-01	9.1700E-01
4	6.2500E-02	9.5760E-01
5	3.1250E-02	9.7857E-01
6	1.5625E-02	9.8923E-01
7	7.8125E-03	9.9460E-01
8	3.9063E-03	9.9730E-01

Raw data of the paragraph: Human PF contains exosomes carrying cardiovascular miRNAs

Raw data are provided for the 3 miRs that are not shown in Figure 3.

	miR-27a-3p	
**Samples**	**Relative Expression PF**	**Relative Expression PLASMA**
1	2.07E-05	6.01E-05
2	9.65E-06	3.15E-05
3	4.54E-05	2.25E-05
4	3.70E-05	6.43E-06
5	1.35E-05	4.66E-05

	miR-15a-5p	
**Samples**	**Relative Expression PF**	**Relative Expression PLASMA**
1	1.08271E-05	0.025207555
2	6.96267E-05	4.30666E-06
3	5.59695E-05	0.036397925
4	1.22658E-05	0.009888723
5	4.18889E-06	0.002733566

	miR-126-3p	
**Samples**	**Relative Expression PF**	**Relative Expression PLASMA**
1	2.3651E-05	1.9199E-04
2	3.0819E-06	9.1028E-05
3	5.2289E-06	3.9714E-05
4	3.5000E-06	3.6629E-05
5	1.5089E-06	2.6752E-05

Relative abundance of the 16 cardiovascular miRNAs and of miR-122 in the aorta and myocardium tissues in and in PF exosomes

Let-7b		
Relative Expression (versus U6) Aorta	Relative Expression (versus U6) Myocardium	Relative Expression (versus cel-miR-39) PF exosome
2.558	1.729	1.17x10^{-3}
2.742	3.797	5.64 x10^{-3}
12.906	4.272	3.0 x10^{-3}
2.092	4.141	1.54 x10^{-3}
3.352		6.69 x10^{-4}

miR-15a-5p		
Relative Expression Aorta	Relative Expression Myocardium	Relative Expression PF exosome
1.000	0.293	1.08 x10^{-5}
0.981	0.574	6.96 x10^{-5}
0.946	0.642	5.60 x10^{-5}
0.985	0.020	1.23 x10^{-5}
0.961		4.19 x10^{-6}

miR-16-5p		
Relative Expression Aorta	Relative Expression Myocardium	Relative Expression PF exosome
0.000	0.171	1.47 x10^{-2}
0.023	0.015	7.97 x10^{-2}
0.001	0.139	4.70 x10^{-2}
0.000	0.020	1.59 x10^{-2}
0.001	0.171	4.63 x10^{-3}

miR-19b-3p		
Relative Expression (versus U6) Aorta	Relative Expression (versus U6) Myocardium	Relative Expression (versus cel-miR-39) PF exosome
0.956	1.102	4.18 x10^{-4}
1.145	1.177	2.77 x10^{-4}
3.694	0.272	9.14 x10^{-4}
0.914	0.540	4.30 x10^{-4}
2.354		1.52 x10^{-4}

miR-21-5p		
**Relative Expression Aorta**	**Relative Expression Myocardium**	**Relative Expression PF exosome**
0.381	1.438	7.619×10^{-4}
0.431	1.025	5.265×10^{-4}
0.360	8.889	4.583×10^{-4}
0.952		4.970×10^{-4}
3.967		9.665×10^{-4}
1.030		
1.954		

miR-22-3p		
**Relative Expression Aorta**	**Relative Expression Myocardium**	**Relative Expression PF exosome**
0.480	0.369	2.75×10^{-4}
0.278	0.118	2.33×10^{-3}
0.865	8.664	1.85×10^{-4}
0.176	0.139	1.50×10^{-4}
1.113		2.64×10^{-4}

miR-23a-3p		
**Relative Expression (versus U6) Aorta**	**Relative Expression (versus U6) Myocardium**	**Relative Expression (versus cel-miR-39) PF exosome**
0.010	0.241	5.118×10^{-5}
0.031	0.147	1.960×10^{-5}
0.031	0.665	1.359×10^{-5}
0.018		9.630×10^{-6}
0.249		4.455×10^{-5}
0.125		
0.181		

miR-24-3p		
**Relative Expression (versus U6) Aorta**	**Relative Expression (versus U6) Myocardium**	**Relative Expression (versus cel-miR-39) PF exosome**
0.563	3.242	4.952×10^{-4}
1.056	3.358	1.085×10^{-3}
0.664	13.878	1.367×10^{-4}
0.066		7.375×10^{-4}
3.698		6.241×10^{-4}
2.438		
9.161		

miR-27a-3p		
**Relative Expression (versus U6) Aorta**	**Relative Expression (versus U6) Myocardium**	**Relative Expression (versus cel-miR-39) PF exosome**
0.314	0.738	2.07 $x10^{-5}$
0.747	0.775	9.65 $x10^{-6}$
0.445	4.286	4.54 $x10^{-5}$
0.414		3.70 $x10^{-5}$
1.580		1.35 $x10^{-5}$
0.913		
1.676		

miR-27b-3p		
**Relative Expression (versus U6) Aorta**	**Relative Expression (versus U6) Myocardium**	**Relative Expression (versus cel-miR-39) PF exosome**
0.318	2.274	4.83 $x10^{-4}$
1.173	2.346	1.55 $x10^{-4}$
4.908	3.811	1.03 $x10^{-5}$
1.699	0.425	2.94 $x10^{-5}$
4.579		1.20 $x10^{-5}$
0.318		
1.173		

miR-29a-3p		
**Relative Expression (versus U6) Aorta**	**Relative Expression (versus U6) Myocardium**	**Relative Expression (versus cel-miR-39) PF exosome**
0.281	1.152	2.24 $x10^{-4}$
0.484	1.919	9.40 $x10^{-4}$
0.375	9.338	3.32 $x10^{-4}$
0.221		1.05 $x10^{-4}$
3.208		9.39 $x10^{-4}$
3.531		
3.557		

miR-29b-3p		
**Relative Expression (versus U6) Aorta**	**Relative Expression (versus U6) Myocardium**	**Relative Expression (versus cel-miR-39) PF exosome**
0.018	0.033	1.34 x10^{-6}
0.018	0.044	6.92 x10^{-6}
0.025	0.269	1.41 x10^{-6}
0.019		2.09 x10^{-6}
0.139		2.45 x10^{-6}
0.163		
0.259		

miR-29c-3p		
**Relative Expression (versus U6) Aorta**	**Relative Expression (versus U6) Myocardium**	**Relative Expression (versus cel-miR-39) PF exosome**
1.693	3.837	7.53 x10^{-4}
2.990	2.540	1.05 x10^{-3}
8.545	1.193	6.69 x10^{-4}
1.053	1.244	7.96 x10^{-4}
4.243		5.11 x10^{-4}

miR-126-3p		
**Relative Expression (versus U6) Aorta**	**Relative Expression (versus U6) Myocardium**	**Relative Expression (versus cel-miR-39) PF exosome**
0.061	1.770	2.37 x10^{-5}
0.059	0.243	3.08 x10^{-6}
0.036	1.586	5.23 x10^{-6}
0.104		3.50 x10^{-6}
2.911		1.51 x10^{-6}
2.136		
3.383		

miR-208a-3p		
**Relative Expression (versus U6) Aorta**	**Relative Expression (versus U6) Myocardium**	**Relative Expression (versus cel-miR-39) PF exosome**
0.991	1.915	1.47 x10^{-2}
1.055	2.959	7.97 x10^{-2}
1.087	0.740	4.70 x10^{-2}
1.003	3.090	1.59 x10^{-2}
1.017		4.63 x10^{-3}

miR-451a		
**Relative Expression (versus U6) Aorta**	**Relative Expression (versus U6) Myocardium**	**Relative Expression (versus cel-miR-39) PF exosome**
0.804	0.027	2.33 x10^{-2}
6.453	3.986	2.22 x10^{-2}
20.112	10.928	2.34 x10^{-2}
17.569	6.277	1.79 x10^{-2}
27.284		5.56 x10^{-3}

miR-122-5p		
**Relative Expression (versus U6) Aorta**	**Relative Expression (versus U6) Myocardium**	**Relative Expression (versus cel-miR-39) PF exosome**
0.000	0.000	0.001
0.000	0.000	0.001
0.000	0.000	0.001
0.000	0.000	0.001
0.000		0.001
0.000		
0.000		

Raw data of the paragraph: PF exosomes contain the RISC components Dicer and Ago-2

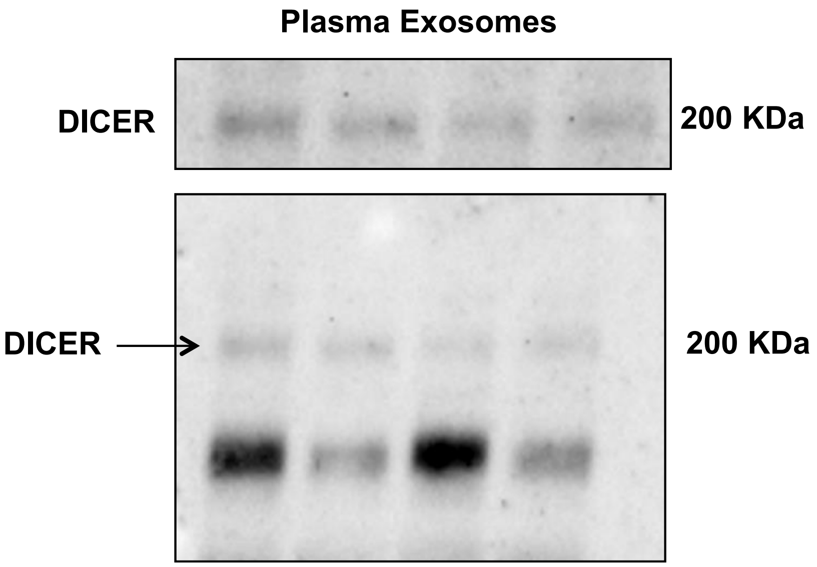

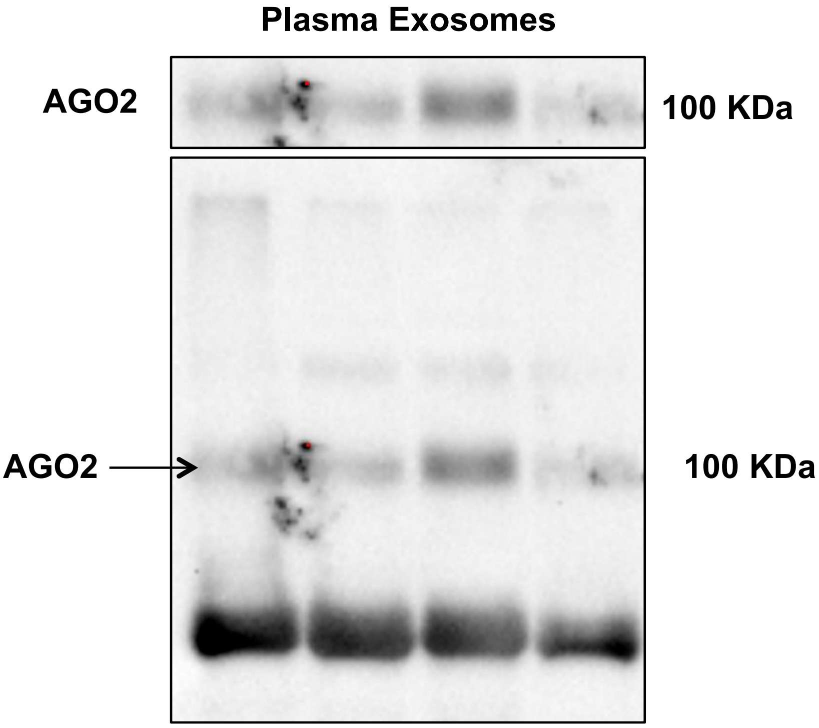

Full unedited gel for Supplementary Figure 2C
PF exosome

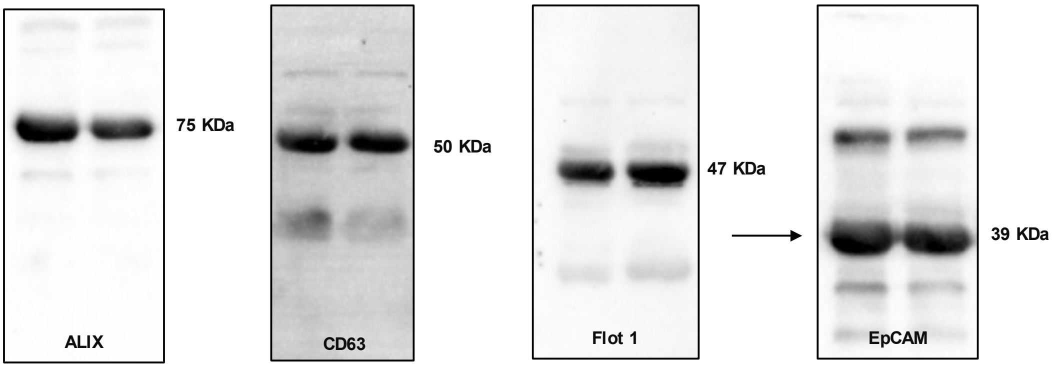

Full unedited gel for Supplementary Figure 2C
Plasma exosome

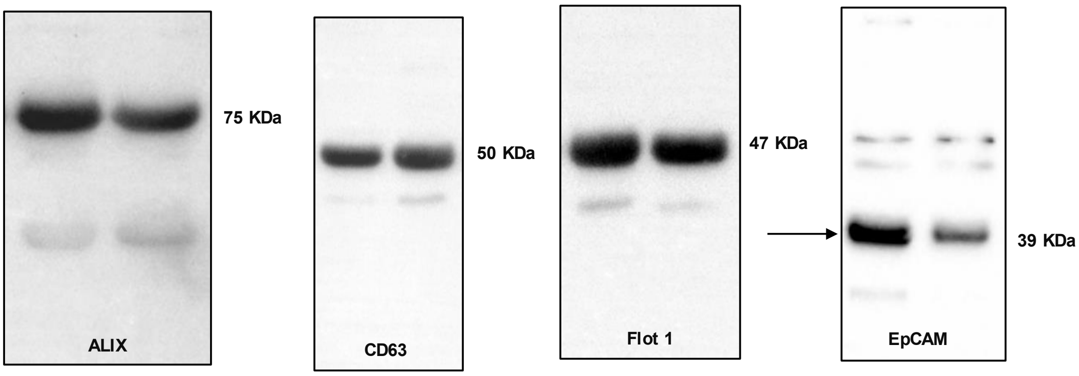

Full unedited gel for Figure 4A

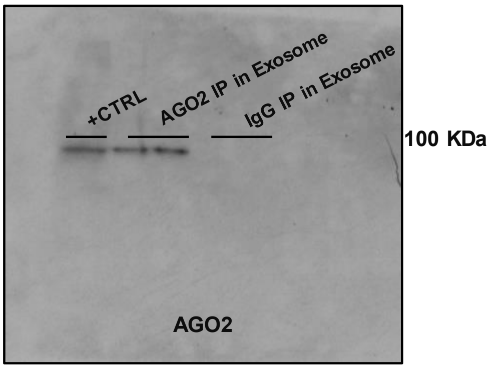

Full unedited gel for Figure 4C

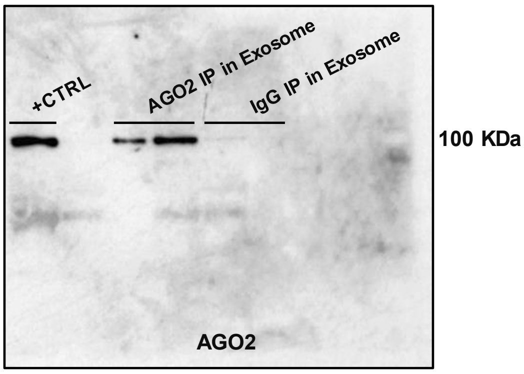

Full unedited gel for Figure 4E

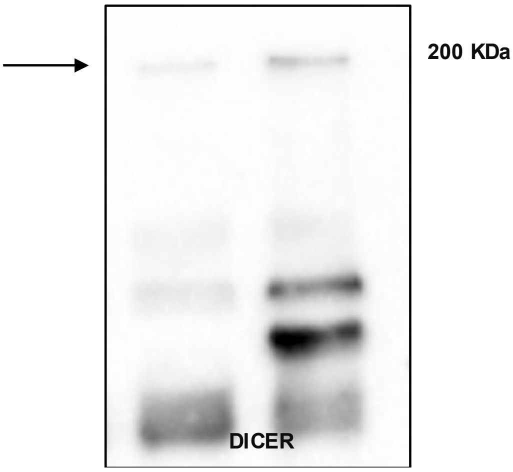

Full unedited gel for Supplementary Figure S3

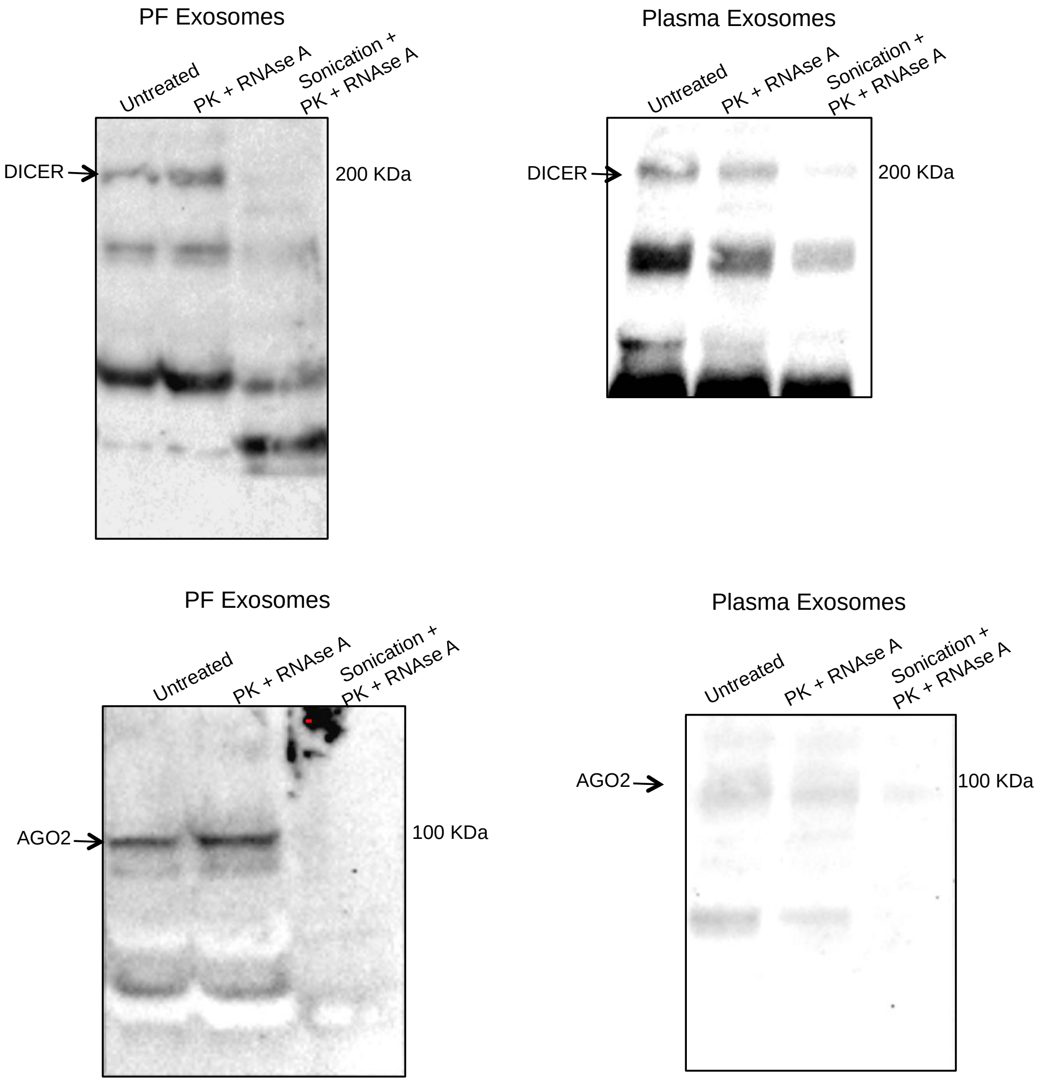

Full unedited gel for Supplementary Figure S9B

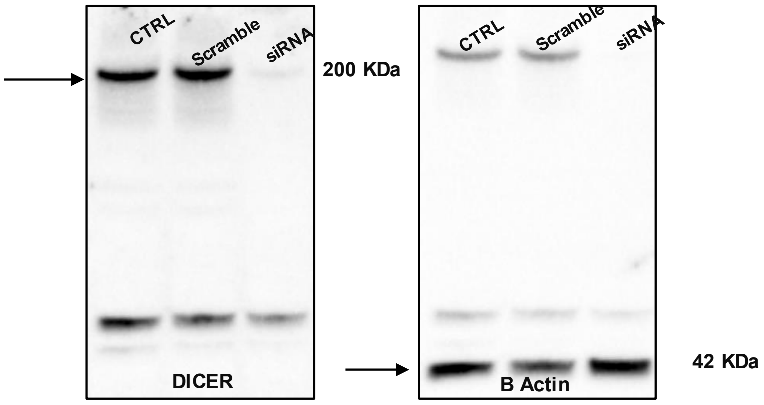

Full unedited gel for Supplementary Figure S10A

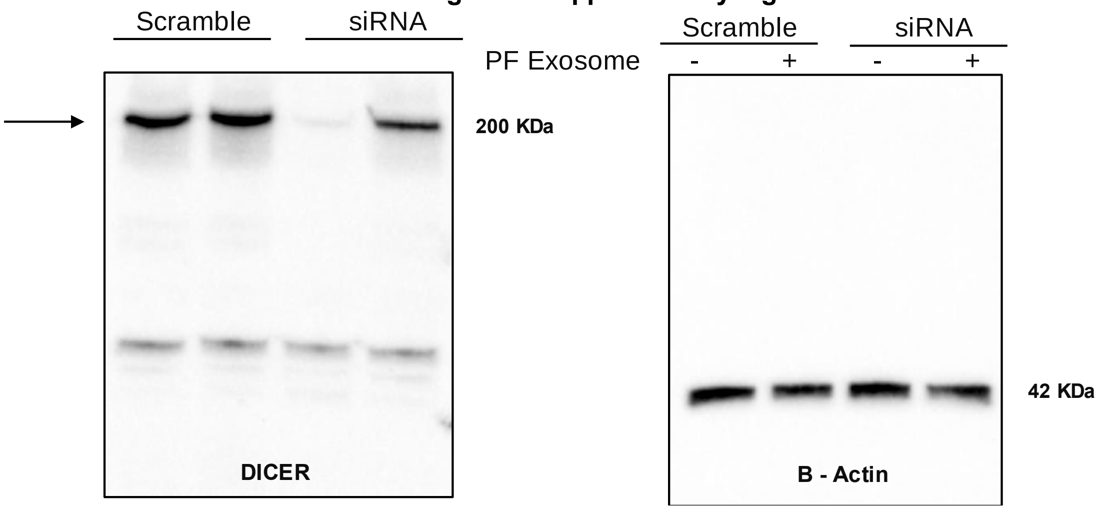

Supplement: Document S1. Figures S1–S10, Tables S1–S3, Raw Data, and Full Unedited Gel [file mmc1.pdf]
