# Supplementary material for: Human Pericardial Fluid Contains Exosomes Enriched with Cardiovascular-Expressed MicroRNAs and Promotes Therapeutic Angiogenesis
Source: Mol Ther. 2017 Feb 1;25(3):679–93. doi: 10.1016/j.ymthe.2016.12.022 (PMC5363195; doi:10.1016/j.ymthe.2016.12.022)

Molecular Therapy
Original Article

Human Pericardial Fluid Contains Exosomes Enriched with Cardiovascular-Expressed MicroRNAs and Promotes Therapeutic Angiogenesis

Cristina Beltrami,[1,2] Marie Besnier,[1] Saran Shantikumar,[1,6] Andrew I.U. Shearn,[1] Cha Rajakaruna,[1] Abas Laftah,[2] Fausto Sessa,[3] Gaia Spinetti,[4] Enrico Petretto,[2,5] Gianni D. Angelini,[1,2] and Costanza Emanueli[1,2]

[1]Bristol Heart Institute, University of Bristol, Bristol BS2 8HW, UK; [2]National Heart and Lung Institute, Imperial College London, London SW3 6NP, UK; [3]Circolo Research Hospital, 57 21100 Varese, Italy; [4]IRCCS MultiMedica, 20099 Milan, Italy; [5]Duke-NUS Medical School, Singapore 169857, Singapore

The pericardial fluid (PF) is contained in the pericardial sac surrounding the heart. MicroRNA (miRNA) exchange via exosomes (endogenous nanoparticles) contributes to cell-to-cell communication. We investigated the hypotheses that the PF is enriched with miRNAs secreted by the heart and that it mediates vascular responses through exosome exchange of miRNAs. The study was developed using leftover material from aortic valve surgery. We found that in comparison with peripheral plasma, the PF contains exosomes enriched with miRNAs co-expressed in patients' myocardium and vasculature. At a functional level, PF exosomes improved survival, proliferation, and networking of cultured endothelial cells (ECs) and restored the angiogenic capacity of ECs depleted (via Dicer silencing) of their endogenous miRNA content. Moreover, PF exosomes improved post-ischemic blood flow recovery and angiogenesis in mice. Mechanistically, (1) let-7b-5p is proangiogenic and inhibits its target gene, *TGFBR1*, in ECs; (2) PF exosomes transfer a functional let-7b-5p to ECs, thus reducing their *TGFBR1* expression; and (3) let-7b-5p depletion in PF exosomes impairs the angiogenic response to these nanoparticles. Collectively, our data support the concept that PF exosomes orchestrate vascular repair via miRNA transfer.

INTRODUCTION

The pericardial fluid (PF) is an ultrafiltrate of plasma contained within the double-walled pericardial sac (also known as pericardium) that surrounds the heart and the roots of the great vessels (ascending aorta, superior and inferior vena cavae, pulmonary arteries and pulmonary veins) bringing blood to and from the heart cells (see Figure S1).[1] The pericardium is composed of two layers: (1) the superficial fibrous pericardium, composed of connective tissue, is continuous with the tunica adventitia of the great blood vessels and anchors the heart to the surrounding walls, and (2) the serous pericardium, composed of mesothelial cells. The serous pericardium is in turn formed by a parietal layer that fuses with the fibrous pericardium at the great vessels roots and the epicardium, which sits on and signals to the myocardium.[2] Proteins introduced at the cavity surface of the endocardium can cross this layer, move between the myocardial cells, and accumulate under the epicardial mesothelium.[3] Moreover, myocardial interstitial fluid can be drained from the subendocardium to the subepicardium via a network of intramyocardial lymphatic capillaries.[4] Subsequently, molecules can be transported from the pericardial cavity to the peripheral circulation by the thoracic duct via the parietal pericardium, by the right lymphatic duct via the right pleural cavity,[5] or through the capillary network and the venous system.[6,7] Cardiac enzymes and troponins are present at higher levels in the PF than the peripheral blood, and increased levels of these molecules are used to aid the diagnosis of fatal myocardial infarct post-mortem.[8,9] Additionally, the PF contains biologically active factors and components, of possible myocardial origin, including atrial and brain natriuretic peptides and endothelin-1.[10] From the above, the myocardial contribution to PF content is apparent, and we reasoned that the PF composition might reflect, at least in part, the myocardium expression profile in health and disease.

MicroRNAs (miRNAs) are post-transcriptional inhibitors of gene expression, which act by provoking either the degradation or functional inhibition of their mRNA targets.[11] The interaction between a miRNA and its targeted mRNAs happens within the RNA-induced silencing complex (RISC), which includes the Argonaute-2 (AGO-2) protein and the enzyme Dicer. Dicer also executes the final maturation step in miRNA biogenesis.[11] miRNAs contribute to cardiovascular development, homeostasis, and disease.[11] miRNAs can be released in biologically active forms by the parental cells, and they are stable in biological fluids, thus representing novel biomarker candidates.[12] In fact, resilience of extracellular miRNAs is granted through different partnerships, such as with extracellular vesicles (EVs), lipoprotein complexes, and RNA-binding proteins or protein complexes, including AGO-2.[11] Exosomes are the smallest (~30–120 nm in size) of the known endogenous EVs. Exosomes have an endosomal

Received 22 September 2016; accepted 25 December 2016;
http://dx.doi.org/10.1016/j.ymthe.2016.12.022.

[6]Present address: Department of Health Sciences, University of Leicester, Leicester LE1 7RH, UK

Correspondence: Costanza Emanueli, Bristol Heart Institute, University of Bristol, Bristol Royal Infirmary, Level 7, Upper Maudlin Street, Bristol BS2 8HW, UK.
E-mail: costanza.emanueli@bristol.ac.uk

This is an open access article under the CC BY license (http://creativecommons.org/licenses/by/4.0/).

origin and are retained within the multivesicular bodies (MVBs) as a result of endosome compartmentalization, being released when the latter fuse with the cell membrane.[13] Exosomal miRNAs can be taken-up by non-parental cells, thus influencing their gene expression and ultimately working as cell-to-cell messengers in local and distant micro-communication mechanisms.[11] The presence of miRNAs in the PF has been recently described.[10] However, whether the PF miRNAs are of cardiac origin, if they are released via EVs, and their functional relevance have remained unaddressed.

Exosomes' molecular cargo is variable and dependent on the parental cell type and the environment. It is therefore intuitive that the actions of exosomes are disparate. Within the cardiovascular area, exosomes were reported to exert direct actions on cultured cardiac myocytes, including promoting hypertrophy[14] and survival.[15] Some pioneering human studies on circulating exosomes have focused at finding new clinical biomarkers, particularly in the oncology area, in which the concept of exosomes as "liquid biopsies" enriched with cancer cell-derived factors is receiving attention.[16] However, the characterization of the exosomes present in human biological fluids and their possible role as cell-to-cell communicators of cardiovascular relevance is still undeveloped. We have recently provided preliminary evidence that exosomes containing high levels of cardiovascular miRNAs are trafficked out of the heart to reach the peripheral blood in patients undergoing cardiac surgery using cardiopulmonary bypass.[17] This is an acute scenario that is associated with ischemia/reperfusion stress to the myocardium.[17] Indeed, we observed that the abundance of plasma circulating exosomes and their content of cardiovascular miRNAs were highly correlated with high sensitive cardiac troponin T, the gold-standard biomarker of myocardial injury.[17] This suggests that the release of exosomes from the heart is regulated in vivo. We reasoned that exosomes released by the heart and large vessels could accumulate in the pericardial space, which could gain new interest as a niche for cardiac biomarker discovery. Moreover, PF exosomes could be involved in yet unreported EV-based crosstalk between cells exposed directly or indirectly to the PF.

RESULTS

Human PF Is Enriched with miRNAs of Potential Cardiovascular Origin

In search for evidence in support of our hypothesis that the PF is enriched with miRNAs released from the heart and thoracic vasculature, under ethical approval, we obtained the PF, peripheral blood-derived plasma, and leftover tissue samples of myocardium (right atrial appendage) and vasculature (ascending thoracic aorta) from surgical patients undergoing aortic valve replacement (AVR) (for study population, see Table S1). An exploratory PCR-based miRNA microarray (Exiqon) on non-pooled, randomly selected samples of whole PF ($n = 3$) revealed the presence of several miRNAs in the PF of the patients (see Table S2). Interestingly, several miRNAs of putative cardiovascular origin appear to be relatively highly expressed (see red text in Table S2). Fifteen of these miRNAs were rationally selected for further analyses, together with miR-208 (supposedly enriched in cardiomyocytes) and the liver-enriched miR-122 (used as a "non-cardiovascular" control) (see Table S3 for the 16 cardiovascular miRNAs that were selected for further investigation and reporting the miRNA particulars). Working on surgical leftover samples, we validated that the 16 bona fide cardiovascular miRNAs were indeed well expressed in the myocardium and/or vasculature of the patients. By contrast and as expected, miR-122 was poorly expressed in the tissue samples (Figure S2). Next, by performing PCR for individual miRNAs on matched PF and plasma samples, we obtained evidence supporting the concept that the PF is enriched with miRNAs released from cardiovascular tissues (Figure 1; Supporting Materials and Methods). These data align with our hypothesis that the PF represents a liquid compartment in which expressional information and executive command from the heart and thoracic vessels are released in the form of miRNAs.

Human PF Contains Exosomes Carrying Cardiovascular miRNAs

We reasoned that the cardiovascular miRNAs present in the PF could, at least in part, be included in exosomal cargos. Nanosight LM10 nanoparticle tracking analyses (NTA) revealed the presence of exosome-sized (30–120 nm) particles in both the PF and plasma, which we analyzed as a reference (Figures 2A and 2B; the rectangles surround exosome-sized particles). Next, exosomes were enriched from the PF and plasma. The quality of the exosome preparations was validated by western blotting for a panel of exosomes antigens (ALIX, CD63, FLOT1, and EPCAM) (Figure 2C) and by transmission electron microscopy (TEM) using gold particles conjugated with an anti-CD63 antibody (Figure 2D) to confirm the exosome identity. The miRNAs already detected in the total PF where also measurable in the PF exosomes. Moreover, for the majority of the individual miRNAs, the expressional differences appreciated comparing whole PF versus whole plasma were confirmed when looking at the exosomal components of the two fluids (Figure 3A). This set of results suggests that miRNAs produced by cells of the heart and heart vessels are, at least in part, transported to the PF via exosomes. Next, to investigate if the miRNAs are differently distributed between the exosomal and non-exosomal fractions of the PF, we calculated the PF exosome/whole PF concentration ratios of the individual miRNAs. The PF exosome/total PF ratios were highly variable between miRNAs (Figure 3B), thus suggesting (1) a non-random incorporation of the miRNAs in the PF exosomes and/or (2) that miRNAs are released by cells via different mechanisms.

PF Exosomes Contain the RISC Components AGO-2 and DICER

To establish if AGO-2-miRNA complexes were present in the PF samples, AGO-2 immunoprecipitation (IP) was performed (Figure 4A) and followed by miRNA RT-qPCR analyses. The majority of our miRNAs were found conjugated to AGO-2 (Figure 4B). The relative expression of the individual miRNAs conjugated to AGO-2 was variable (Figure 4B), and it did not follow the same trends of exosomal miRNA expression (Figure 3A). Interestingly, the majority of studied miRNAs were co-expressed in exosomes and AGO-2 complexes, suggesting the possibility that PF exosomes contain

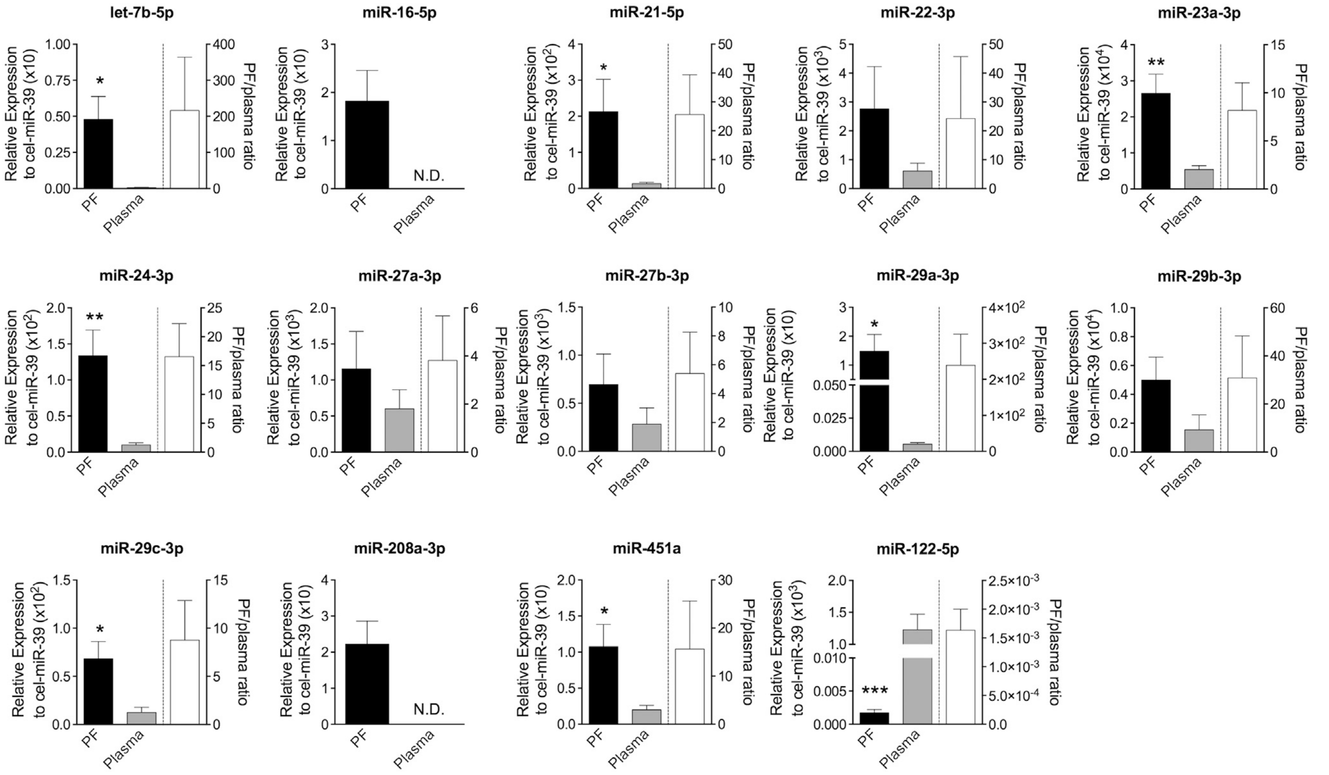

Figure 1. Human PF Is Enriched with MicroRNAs of Potential Cardiovascular Origin

The expression of selected microRNAs (miRNAs) was measured (RT-qPCR) in paired PF (black column) and peripheral plasma (gray column) samples. The PF/plasma concentration ratios of individual miRNAs are plotted on the right side of the graphs. Exogenous spike-in cel-miR-39 was the normalizer. MiR-122-5p was used as a non-cardiovascular control. N.D., not detectable. All values are mean + SEM. *p ≤ 0.05, **p ≤ 0.01, and ***p ≤ 0.001 versus plasma (unpaired Student's t test); n = 5–9.

AGO-miRNA complexes. In line with this, AGO-2 was detected (by western blot analysis) in the PF exosome preparations (Figure 4C). Moreover, AGO-2-IP of PF exosomes, followed by PCR for a randomly selected subset of the miRNAs expressed in the AGO2 complexes, confirmed the presence of AGO-2-miRNA complexes in the exosomal compartment of human PF (Figure 4D). Also in this case, the relative expression of the individual exosomal miRNAs conjugated to AGO-2 was variable, with let-7b-5b appearing particularly enriched in the AGO-2 complex (Figure 4D). Dicer was also present within the PF exosomes (Figure 4E). Similarly to PF, plasma exosomes also contained both Dicer and AGO-2 (Supporting Materials and Methods). When the exosome membrane was not previously destroyed by sonication, the exosomal miRNAs (let-7b-5p and miR-122) and proteins (DICER and AGO2) were resistant to proteinase K (PK) and RNase A digestion (Figure S3). This favors the hypothesis that the miRNAs, DICER and AGO-2 were indeed encapsulated inside the exosomes, rather than being merely co-precipitated with them. Taken together, the above data provide evidence that exosomes contain miRNAs that are co-expressed and possibly physically associated with the RISC. It is therefore possible that exosomes deliver miRNAs together with a RISC machinery ready to act in recipient cells, thus immediately eliciting expressional changes commanding functional responses.

PF Exosomes Are Incorporated by Cultured ECs and Enhance Their Angiogenic Capacity

To investigate whether the PF exosomes are functionally active, we tested them on cultured ECs focusing on responses that are conducive to angiogenesis. Patient-derived plasma exosomes were used for comparison. PF or plasma exosomes clusters, labeled with a green fluorescent marker, were added to cultured ECs, and their internalization was confirmed by confocal microscopy 3D and reconstruction of the confocal image z stacks (Figures 5A and S6A). Two negative controls were included to confirm the specificity of the exosome uptake derived from the PF (Figure S4). Next, exosomes were studied in cell biology assays developed on cells kept under hypoxia. In comparison to the PBS control, PF-derived exosomes inhibited EC apoptosis (Figure 5B) and increased EC proliferation (Figure 5C). Moreover, the PF exosomes, in comparison to PBS control and PF-derived exosomes, were also able to promote the formation of capillary-like cellular networks on Matrigel (Figures 5D, 5E, and S5), while the exosome-depleted PF fraction had no effect. Plasma exosomes did not affect any of the tested EC functions (Figures S6B–S6E). Exosome concentration-response curves confirmed the inactivity of plasma exosomes on ECs (Figures S6F and S6G) in the concentration range whereas PF exosomes increased EC survival and proliferation (Figures S6H and S6I). We speculate that the differential behavior of

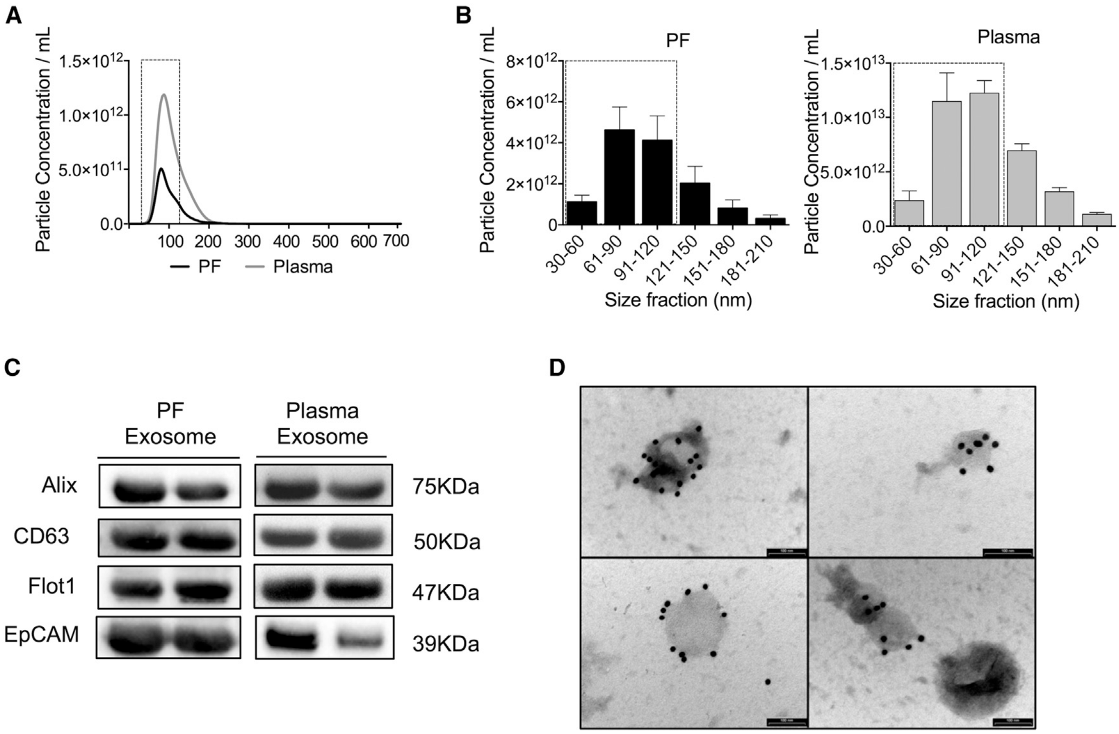

Figure 2. PF Contains Nanoparticles of the Size and Antigenic Profile of Exosomes

(A and B) Nanoparticle tracking analysis (NTA) was used to quantify the concentration (A) and size distribution (B) of particles in the total PF and plasma. The dotted rectangles evidence exosome-sized vesicles (30–120 nm). (C and D) PF and plasma exosome preparations were positive for the exosomal markers ALIX, FLOT1, EpCAM and CD63. Transmission electron microscopy (TEM) using a gold particle-conjugated anti-CD63 antibody further confirmed exosome identity. In the bottom right corner of (D), next to CD63-immunoreactive exosomes, is shown a larger unreactive cytoplasmic vesicle as negative control (the scale bar represents 100 nm). Unpaired two-tailed Student's t test was applied. All values are mean + SEM; n = 5.

exosomes from PF and plasma might be in part related to differences in their miRNA cargos.

The PF-Enriched let-7b-5p Is a Proangiogenic miRNA

Next, we questioned whether exosomal miRNAs contributed to the angiogenic responses elicited by the PF exosomes. To answer this, we focused on let-7b-5p, which was chosen because it was highly expressed in the PF exosomes (Figure 3A), including as conjugated with AGO-2 (Figure 4D), and because other members of the Let-7 family were known to stimulate angiogenesis.[18,19] Expressional data were validated using standard curves. In fact, we found that in comparison with plasma, the whole PF contained higher let-7b-5p and lower miR-122 copy numbers (Figures S7A and S7B). Moreover, we confirmed the presence of let-7b-5p in PF exosomes (Figure S7C). By transfecting ECs with a let-7b-5p mimic, a let-7b-5p inhibitor, or their respective controls (see Figure 6A for validation of the impact of transfection on let-7b-5p in ECs), we demonstrated that let-7b-5p promotes angiogenesis in vitro. In fact, increased let-7b-5p improved capillary-like tube formation on Matrigel (Figure 6B, top), while let-7b-5p inhibition produced the opposite effect (Figure 6B, bottom), and decreased EC proliferation (Figure 6D, right). By contrast, let-7b-5p did not affect the survival of cultured ECs (Figure 6C). We also determined whether the expression of three previously validated direct target genes of let-7b-5p was affected by forcing let-7b-5p expressional changes in ECs. Increased let-7b-5p reduced the mRNA expression of the antiangiogenic *TGFBR1* only[20,21] (Figure 6E), leaving unchanged the other two potential target genes, *LOX-1* and *CASPASE 3* (Figures S8A and S8B). Therefore, the latter were not taken forward for further analyses.

The Angiogenic Action of PF Exosomes Is Partially Mediated by let-7b-5p

We next interrogated the possibility that let-7b-5p could be transferred from PF exosomes to ECs and the expressional and functional impact of exosomal let-7b-5p uptake by recipient ECs. For these experiments, we adopted a model in which the endogenous expression of miRNAs is reduced. It was previously reported that miRNA biogenesis, and hence intracellular miRNA levels, are decreased after *DICER* silencing in cultured ECs. Moreover, *DICER* knockdown (KD) reportedly impairs the angiogenic capacity of cultured ECs.[22] We initially confirmed that *DICER* KD resulted in reduced DICER gene and protein expression (Figures S9A and S9B) and impaired the angiogenic potential of ECs (Figures S9C and S9D). Moreover, as expected, *DICER* KD reduced let-7b-5p expression (Figure 7A). Next, ECs with either *DICER* KD or a preserved *DICER* expression were stimulated with our PF exosomes. Treatment with PF exosomes

A

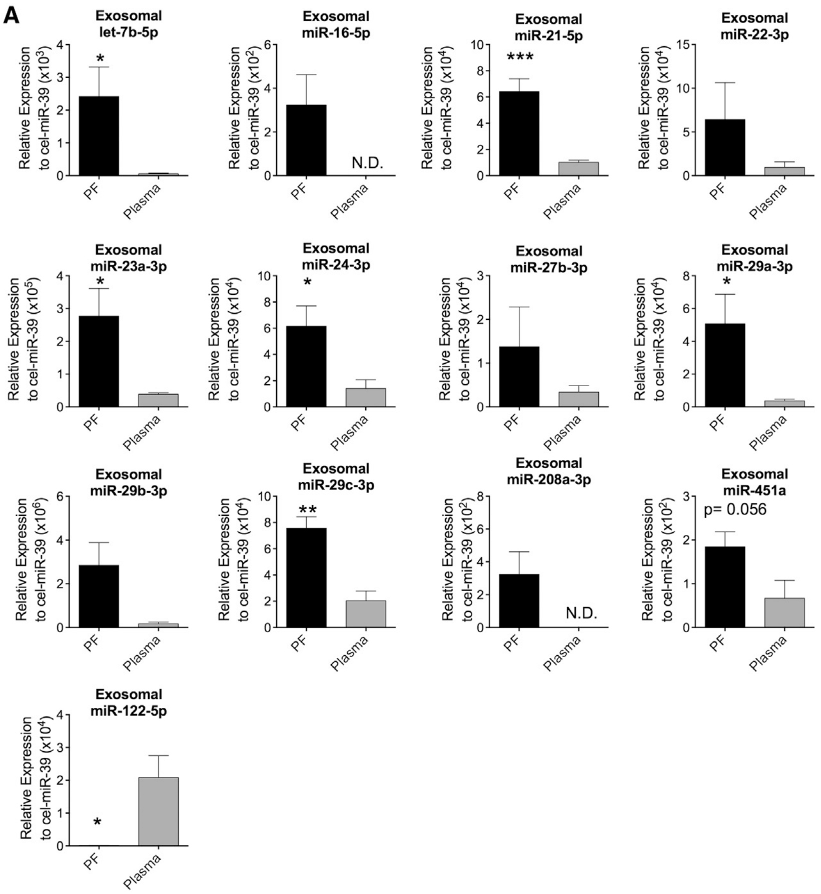

B

miRNAs	PF Exosome/Total PF ratio (Mean ± s.e.m.)
let-7b-5p	$0.10 \pm 2.6x10^{-2}$
miR-15a-5p	0.32±0.17
miR-16-5p	0.24 ± 0.10
miR-19-3p	0.26 ± 0.07
miR-21-5p	0.10 ± 0.06
miR-22-3p	0.43 ± 0.13
miR-23a-3p	$1.46x10^{-3} \pm 6.6x10^{-4}$
miR-24-3p	$7.81x10^{-2} \pm 4x10^{-2}$
miR-27a-3p	$8.4x10^{-2} \pm 5.97x10^{-2}$
miR-27b-3p	$7.37x10^{-3} \pm 1.9x10^{-3}$
miR-29a-3p	$1.2210^{-2} \pm 6.8x10^{-3}$
miR-29b-3p	$2.49x10^{-2} \pm 2.1x10^{-2}$
miR-29c-3p	$4.90x10^{-1} \pm 1.90x10^{-1}$
miR-126-3p	$7.36x10^{-3} \pm 1.9x10^{-1}$
miR-208a-3p	$1.55x10^{-1} \pm 5.88x10^{-2}$
miR-451a	$1.88x10^{-1} \pm 2.27x10^{-2}$

Figure 3. Human PF Exosomes Carry Cardiovascular miRNAs

(A) miRNA level normalized to the spike-in cel-miR-39. (B) PF exosome/total PF ratios calculated for each miRNA. All values are mean + SEM. *p ≤ 0.05, **p ≤ 0.01, and ***p ≤ 0.001 versus plasma (unpaired Student's t test); n = 5.

restored intracellular let-7b-5p expression, which had been compromised by *DICER* KD (Figure 7A), thus supporting the hypothesis that this proangiogenic miRNA is transferred from the PF exosomes to the recipient ECs. In line with the hypothesis that the let-7b-5p delivered from PF exosomes into ECs is functionally active, ECs treated with PF exosomes responded with a decreased mRNA expression of the miRNA target gene *TGFBR1* (Figure 7B). In ECs with *DICER* KD, treatment with PF exosomes was additionally able to restore DICER expression at protein (Figure S10A), but not mRNA, levels (data not shown). This, together with the aforementioned finding of DICER presence in the exosomes (Figure 4E), further suggests that PF exosomes pass on miRNAs and other components of the RISC machinery to recipient cells. Finally, and in line with their induced let-7b-5p and *TGFBR1* expressional changes, PF exosomes restored the angiogenic capacity of *DICER* KO-ECs (Figures 7C and 7D). To further investigate the transfer of PF exosomal let-7b-5p to ECs and its functional consequence, we preventively suppressed let-7b-5p inside the exosomes. The reduction of exosomal let-7b-5p in PF exosomes transfected with the miRNA inhibitor was confirmed by qPCR (Figure S10B). Moreover, the PF let-7b-5p KD-exosomes could not (1) restore let-7b-5p levels (Figure 7A), (2) decrease *TGFBR1* expression (Figure 7B), or (3) improve angiogenesis (Figures 7C and 7D) in recipient *DICER*-KD ECs. Taken together, these data support the hypothesis that PF exosomes are functionally active and stimulate angiogenesis, at least in part, via the passage of the proangiogenic let-7b-5p to ECs.

PF-Derived Exosomes Promote Post-ischemic Angiogenesis and Blood Flow Recovery

We investigated whether the angiogenic capacity shown by PF exosomes in vitro may translate into improvement of angiogenesis and blood flow recovery in vivo. The therapeutic potential of PF exosomes was studied in a mouse model of ischemia, in which local stimulation of angiogenesis represents a possibility to improve tissue perfusion. As controls, we used PBS and plasma exosomes, which we had proved unable to induce in vitro angiogenesis (Figures S6B–S6G). Mice with surgically induced unilateral limb ischemia were injected with either PBS or exosomes (100 μg/mouse) directly into their ischemic adductor muscles. Compared with PBS, at 7 days from delivery, the PF-derived exosomes improved post-ischemic blood flow recovery

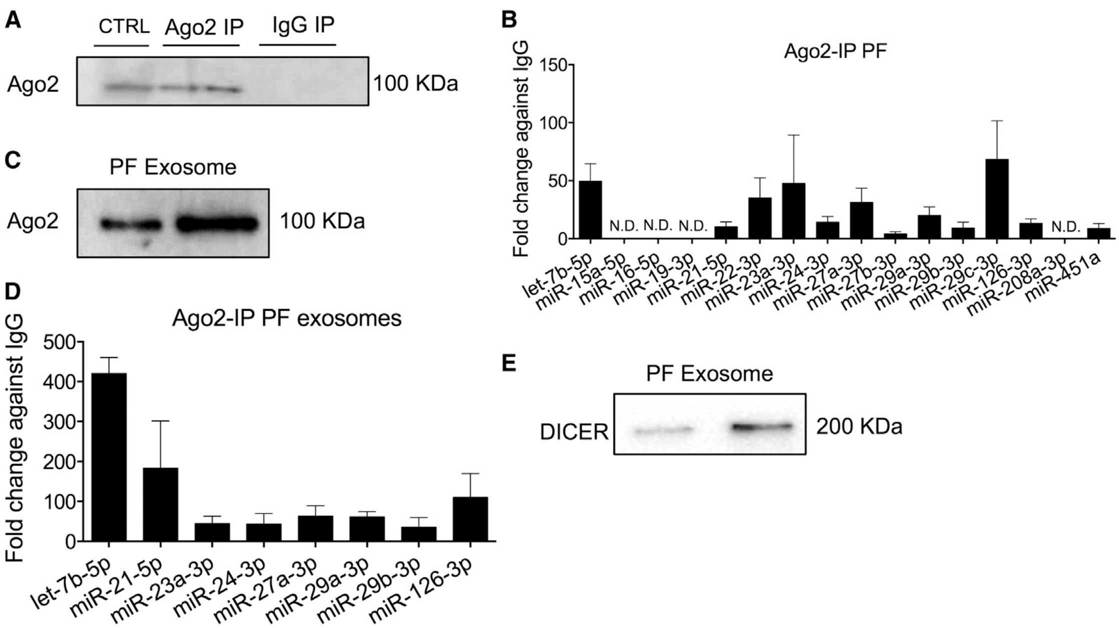

Figure 4. PF Exosomes Contain DICER and AGO-2 Protein

(A) Validation by immunoblotting of AGO-2 immunoprecipitation (IP) performed on human PF samples using AGO-2 antibody. Mouse non-specific IgG antibody was used as control for the IP. ECs were used as positive control (CTRL). (B) The miRNA expression after AGO-2 IP is presented as fold enrichment relative to IgG; mean + SEM; n = 5. (C) AGO-2 in exosomes enriched from PF samples (representative western blot images). (D) AGO-2 IP was performed on exosomes enriched from PF samples. miRNA expression is expressed as fold enrichment in the AGO-2 IP relative to IgG; mean + SEM; n = 2. (E) Representative western blot images of DICER protein incorporated in the exosomes.

(Figures 8A and 8B), reduced the incidence of ischemia-induced toe necrosis (Figure 8C), and improved capillary density in ischemic muscles (Figures 8D and 8E). The positive impact of PF exosomes on blood flow recovery was not sustained over time (data not shown). Plasma exosomes were not able to reproduce any of the above therapeutic benefits (Figures 8A–8E). Importantly, after transfer of PF exosomes into ischemic limb muscles, there was a trend toward increased let-7b-5p expression ($p = 0.18$; Figure 8F) and a significant decrease in *Tgfbr1* expression ($p < 0.05$; Figure 8G), which could be explained by an amplified functional response at the target gene level of the PF exosome-mediated delivery of active let-7b-5p.

In conclusion, the exosomes contained in human PF have shown the capacity to induce vascular protective and reparative functions in vitro and in vivo, suggesting their importance in the context of cardiovascular physiopathology.

DISCUSSION

Vascular cells, cardiomyocytes, cardiac fibroblasts, and cardiac progenitor cells reportedly secrete miRNA-containing exosomes in culture systems.[23] Functional studies focusing on exosomes in human biological fluids are still lacking. Such studies are of importance to characterize the relevance of exosome-based communication in human pathophysiology. We have reported the vascular regenerative potential of exosomes prepared from biological fluids collected from cardiovascular patients.

Exosomes produced from stem and progenitor cells, including the ones isolated from cultured adult cardiac progenitor cells (CPCs), which have been claimed to reside in the human heart and contribute to myocardial regeneration, have shown proangiogenic effects.[24,25] However, the in vivo abundance and physiological relevance of CPCs are currently intensively questioned,[26,27] thus discouraging the speculation that exosomes released in vivo by CPCs play a major role in heart homeostasis. Stem and progenitor cells still represent hope for vascular regeneration. In a seminal paper, Sahoo et al.[28] showed that bone marrow mononuclear cell (MNC)-derived CD34-positive cells promote therapeutic angiogenesis in vitro and in vivo via paracrine actions that can be recapitulated by their exosomes, but not by exosomes from MNCs. Later studies have expanded on this to recognize that proangiogenic exosomes are released in culture by the different types of progenitor cells so far trialled in patients with heart ischemia.[13] These findings open new exciting therapeutic avenues but do not address the contribution of endogenous exosomes to cardiovascular physiopathology. Additionally, not all exosomes stimulate angiogenesis. As an example, exosomes from diabetic cardiomyocytes reportedly inhibited endothelial cell (EC) survival and angiogenesis by the transfer of miRNA-320.[29]

The angiogenic response to PF exosomes described in our study appears “specific” to PF-derived exosomes because it could not be reproduced using exosomes prepared from the plasma of the same patients. Evidence from the literature suggests that peripheral plasma

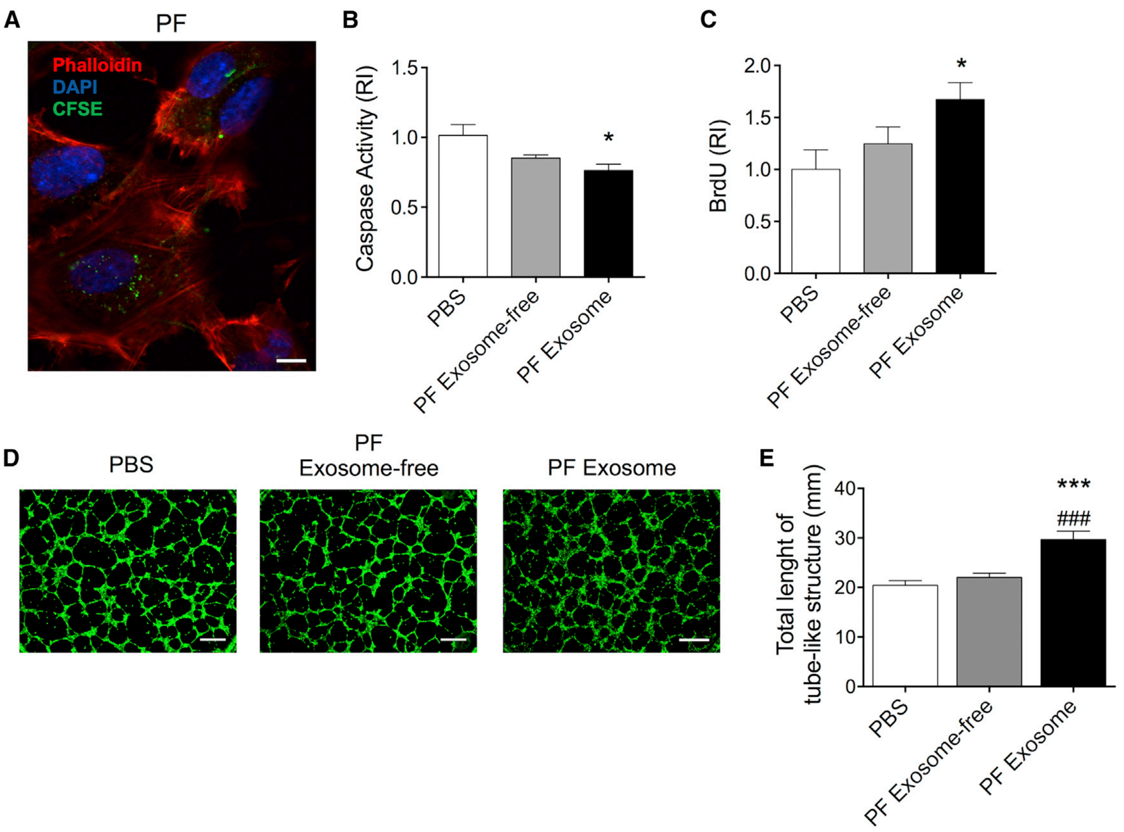

Figure 5. PF-Derived Exosomes Are Incorporated by Cultured ECs and Enhance Their Angiogenic Capacity

(A) PF-derived exosomes (10 μg/ml) stained using carboxyfluorescein succinimidyl ester (CFSE, in green) were incubated with ECs for 24 hr. Next, cells were stained with phalloidin (red) and DAPI (blue) (the scale bar represents 25 μm). (B and C) Column graphs show (B) EC apoptosis (measured by caspase-3 activity assay) and (C) EC proliferation (measured by BrdU incorporation) after treatment with 10 μg/ml of either PF- exosomes (black columns) or exosome-depleted PF (gray columns). PBS was used as an additional control (open columns); n = 7. (D) Photomicrographs show endothelial network formation on Matrigel (the scale bar represents 200 μm); 2.5× magnification. (E) Bar graphs show Matrigel assay quantification (total length of EC tube-like structures). All values are mean + SEM. *$p \leq 0.05$ and ***$p \leq 0.001$ versus PBS, $^{\#\#\#}p \leq 0.001$ versus PF exosome-free (one-way ANOVA, Dunnett's post hoc test); n = 6.

exosomes could elicit different cardioprotective functions.[15] Alternatively, the type of patients we have studied could present with alterations in their plasma exosomes. This study was conducted on patients with aortic stenosis undergoing aortic valve replacement. These patients are afflicted by left ventricular hypertrophy. Cardiac hypertrophy is associated with complex multicellular alterations, including hypertrophy, fibrosis, and inflammation.[30] These actions are brought about by cardiac and non-cardiac cells through a variety of cell-to-cell communication pathways.[30] In particular, during cardiac hypertrophy, the contractile function of the myocardium must be sustained by phenotypic changes of the capillary ECs leading to angiogenesis. Previous studies identified that proangiogenic factors released by cardiac myocytes sustain the capillary density and the oxygen supply.[31,32] Our data are in line with the new paradigm that PF exosomes and their miRNAs contribute to modulating the growth of new capillary vessels, thus protecting the heart from an accelerated failure. In support of this hypothesis, we found that the PF exosomes isolated from cardiovascular patients promote angiogenesis in vitro and in vivo. However, it is also possible that compared with healthy controls, PF exosomes from AVR patients have a reduced altered proangiogenic potential. This question could not be investigated in our human studies and will need to be deferred to work on suitable animal models.

We have mechanistically implicated a newly defined pro-angiogenic miRNA, let-7b-5p, in the proangiogenic responses to PF exosomes. However, in addition to let-7b-5p and other proangiogenic miRNAs, PF exosomes also contain several anti-angiogenic miRNAs (Table S3). The overall proangiogenic responses to the PF exosomes might be dictated by a functional prevalence of proangiogenic miRNAs that are simultaneously transferred by the same nanoparticles. However we cannot discount the possibility that anti-angiogenic miRNAs are enriched in a subset of exosomes that are taken up with reduced efficiency by the ECs. Moreover, the expression of individual miRNAs in a cell context and possibly in the exosomes does not always reflect the level of the individual miRNAs binding to AGO-2 and hence their

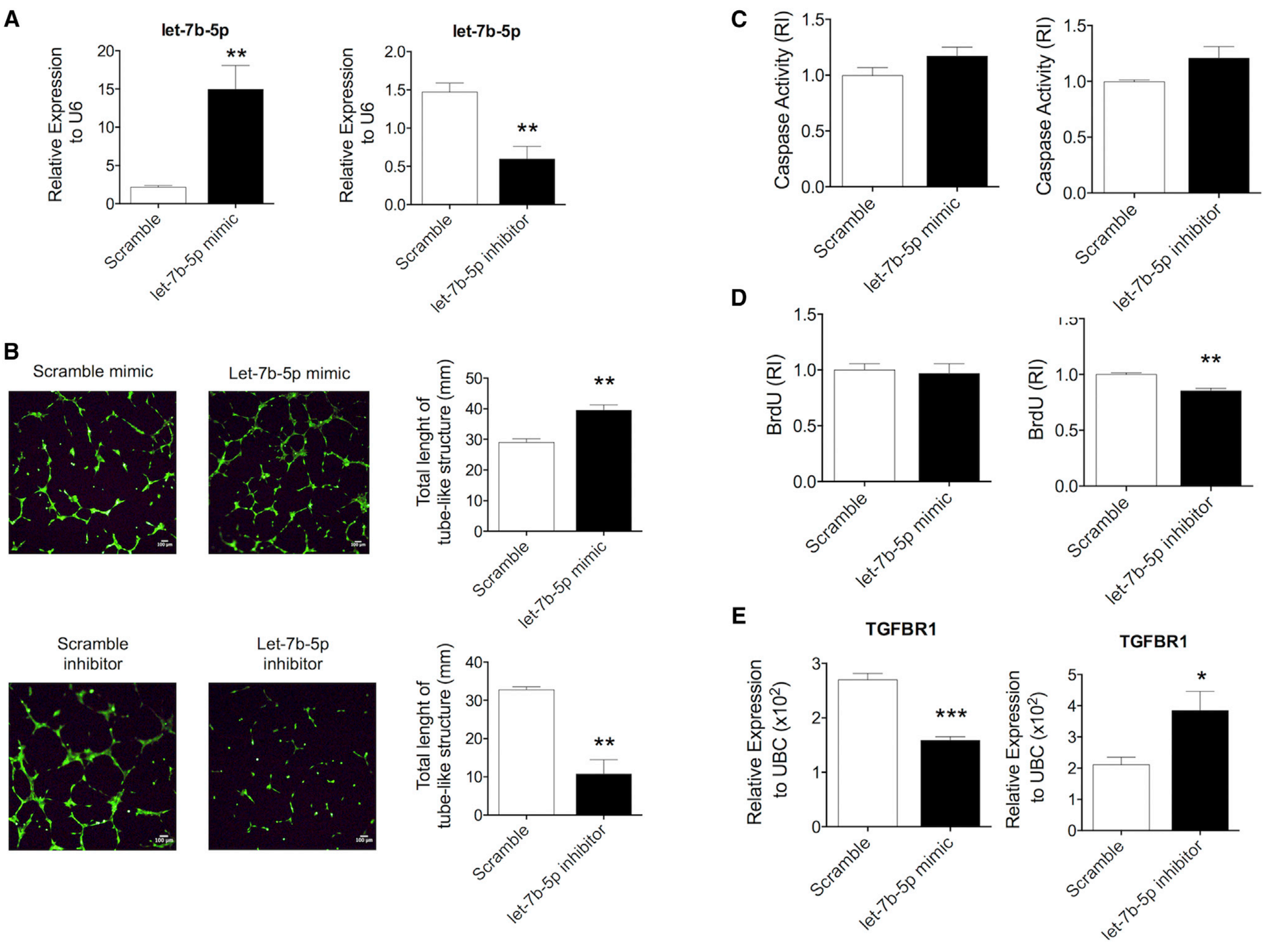

Figure 6. The PF-Enriched let-7b-5p Is a Proangiogenic MicroRNA

(A) ECs were transfected with either a let-7b-5p mimic (right) or a let-7b-5p inhibitor (left). The random sequence anti-miR miRNA inhibitor (scramble) was used as control. Efficiency of let-7b-5p transfection was assessed by qPCR using U6 as a normalizer. (B) Matrigel assay photomicrograph (the scale bar represents 100 μm) and quantification (total length of tube-like structures), 5× magnification. (C and D) Caspase activity (C) and BrdU incorporation (D) of ECs transfected as previously described. (E) Relative expression of the let-7b-5p direct target gene *TGFBR1* (measured by PCR using ubiquitin C [UBC] as normalizer). All values are mean + SEM. *$p \leq 0.05$, **$p \leq 0.01$, and ***$p \leq 0.001$ versus the respective scramble control (unpaired Student's t test); n = 4.

functionality. In fact, it is emerging that the association between miRNAs and the RISC complex is a regulated process. As an example, Krell et al.[33] showed that following DNA damage, P53 interacts with AGO-2 to induce or reduce AGO-2's association with subsets of miRNAs, including multiple let-7 family members.

Our study provides the first evidence that the pericardial space might represent a special compartment, in which functional forms of miRNAs that are released from the heart are enriched in comparison with the peripheral circulation. Because of their resilience to degradation in the extracellular space, miRNAs have been widely considered as new potential clinical biomarkers. Pioneer miRNA-oriented biomarker discovery studies have been developed by measuring miRNAs in the peripheral circulation. However, the emerging evidence for the multi-cellular and multi-tissue expression of virtually any of the known miRNAs, together with the understanding that several processes (release from different cells and organs, potential uptake from the circulation into other tissues, urinary excretion, etc.) dictate the miRNA concentration in the peripheral blood, diminish the potential value of miRNAs as peripheral blood circulating biomarkers. We propose that miRNA expression in the PF is more indicative of cardiac miRNA expression and secretion. In defense of our hypothesis, we have shown increased cardiovascular miRNA expression in the total PF and in PF-derived exosomes in comparison with the corresponding plasma counterparts. Exosomes have been already suggested as "liquid biopsies."[34] Our study stimulates the original hypothesis that obtaining such liquid biopsies out of the PF (when accessible) rather than from the peripheral blood could

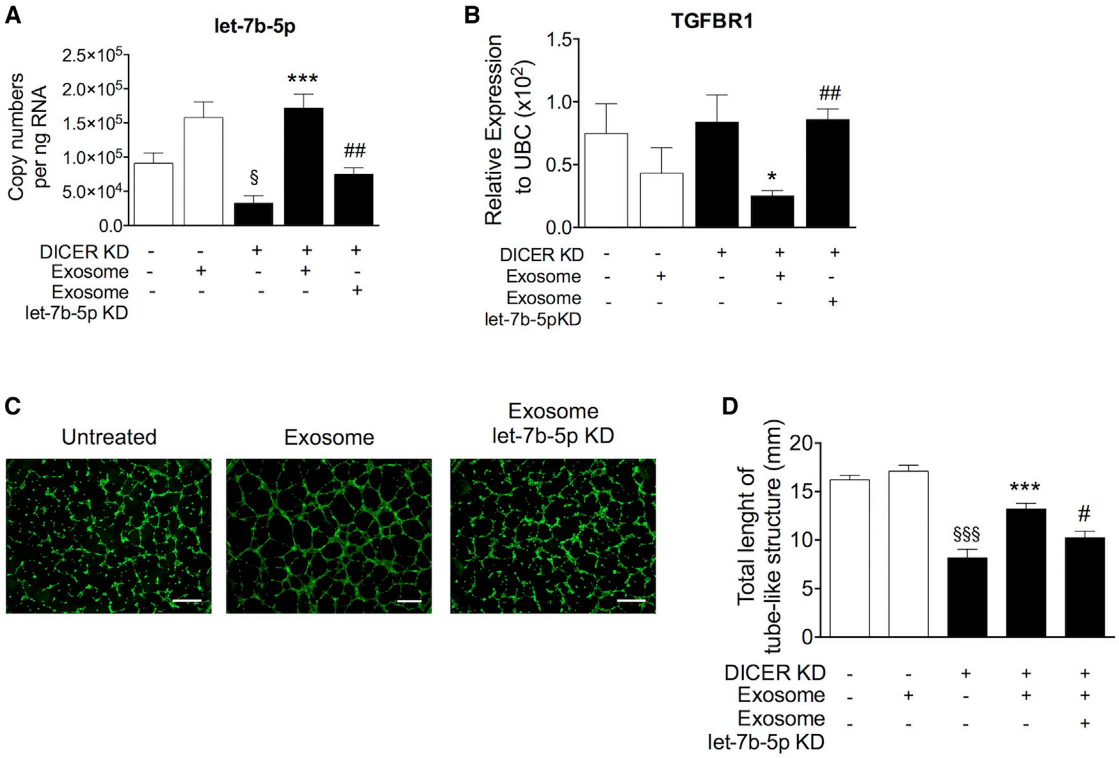

Figure 7. Let-7b-5p Underpins the Angiogenic Action of PF Exosomes

ECs were transfected with either a sequence that exhibits no homology to the human genome (scramble) or *DICER* silencing RNA (siRNA) to knock down (KD) *DICER* for 24 hr, before being treated with either naive PF exosomes or PF exosomes previously depleted of their let-7b-5p content (exosome let-7b-5p KD). (A and B) Columns graphs show the relative expression of (A) let-7b-5p and (B) *TGFBR1* in the exosome recipient ECs and untreated cell controls; n = 4. (C) Photomicrographs (the scale bar represents 100 μm) and (D) quantification of Matrigel assays performed using ECs treated as previously indicated; n = 5; 2.5× magnification. All values are mean + SEM. $^{§}p \leq 0.05$ and $^{§§§}p \leq 0.001$ versus scramble, $^{*}p \leq 0.05$ and $^{***}p \leq 0.001$ versus *DICER* KD, $^{\#}p \leq 0.05$ and $^{\#\#}p \leq 0.01$ *DICER* KD treated with naive PF exosomes (one-way ANOVA, Tukey's post hoc test).

increase the diagnostic and prognostic power of exosomal biomarkers. Specifically designed clinical studies are necessary to validate the biomarker value of PF miRNAs.

In this study, we have used human exosomes in immunocompetent mice. It was already published that exosomes derived from human progenitor cells can induce therapeutic effects in immunocompetent small[35] and large[36] animal models. Nonetheless, we cannot discount the possibility that an immune response could have reduced the therapeutic efficacy of the exosomes. In line with this possibility, the positive impact of PF exosomes on blood flow recovery was not sustained over time. On the other hand, exosomes have been suggested to be able to modulate immunity.[37] Given the increasingly recognized importance of the crosstalk between the immune and cardiovascular systems in health and disease, we would not exclude that exosomes could influence cardiovascular responses partly by modulating the immune system.

The interest in exosomes as new therapeutic tools is growing.[28,38] The production of exosomes from patients' own cells and biological fluids could represent a new avenue for autologous regenerative therapies. Additionally, careful characterization of endogenous exosomes with proven therapeutic capacity should help with the future fabrication of nature-inspired artificial vesicles carrying a defined therapeutic cargo, which could represent an option for developing exosomes into off-the-shelf therapeutic products to be used in different cardiac and non-cardiac conditions.

Study Limitations

First, we have conducted an exploratory study using anonymized surgical leftover material from patients undergoing AVR. As such, our study has been able to provide the first characterization of the PF exosomal compartment but not to investigate its biomarker value. Prospective studies collecting clinical samples and data (at baseline and follow-up) from surgical and non-surgical patients are next required. Interestingly, the PF represents a leftover material from most cardiac surgeries and is also sampled for diagnostic purposes in non-surgical patients.[39,40] However, this fluid is not always accessible, thus limiting the translational potential to particular clinical conditions and to restricted time windows. Second, we have focused on the proangiogenic actions elicited by PF exosomes. We already know that PF exosomes are taken up by additional types of heart cells

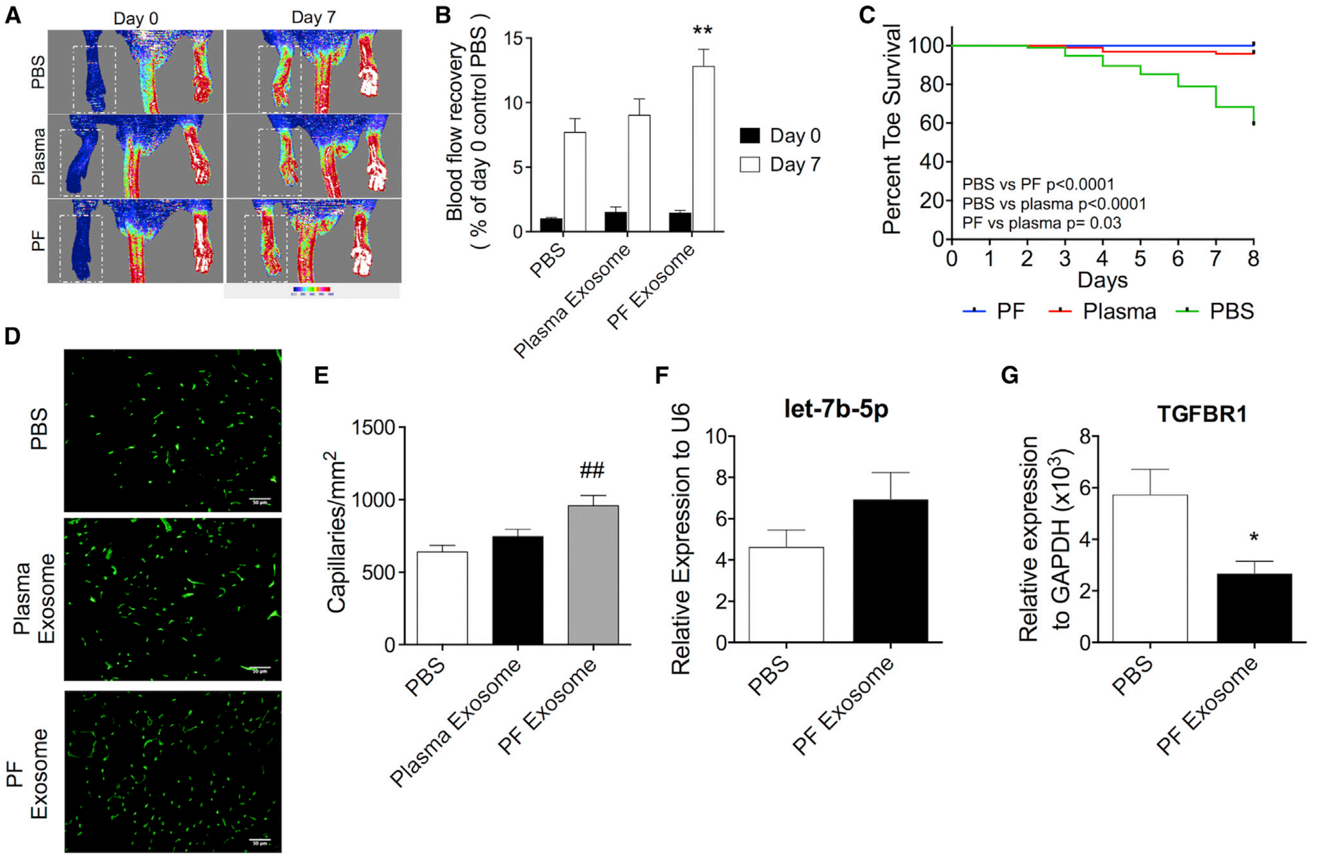

Figure 8. PF-Derived Exosomes Promote Reparative Angiogenesis and Blood Flow Recovery in a Mouse Limb Ischemia Model

Unilateral limb ischemia was surgically induced in CD1 mice. Next, mice were injected in their ischemic adductor muscle with 100 μg of exosomes from either the PF or plasma. A control group received PBS. (A) Representative color laser Doppler images of lower limb perfusion at baseline and at day 7 post-ischemia. (B) The blood flow recovery to the ischemic foot was calculated as percentage versus the contralateral foot (n = 20 mice/group). (C) Percentage of necrotic toes in animals treated with PBS, PF, or plasma-derived exosomes. $^{**}p < 0.01$ versus PBS. (D) Representative immunofluorescent images of ischemic adductor muscle sections after staining with green fluorescent isolectin-B4. (E) Capillary densities in the ischemic adductors at 7 days post-ischemia induction (n = 8 per group). Average capillary density was determined from eight randomly selected high-power fields (magnification 20×). $^{\#\#}p < 0.01$ versus PBS. (F and G) Let-7b-5p (F) and *Tgfbr1* (G) expression in ischemic mouse adductors injected with PF exosomes or PBS (control); n = 5. $^{*}p \leq 0.05$ versus PBS. All values are mean + SEM.

(C.B., unpublished data) and further studies will be necessary to disclose their full spectrum of actions. Third, we have concentrated our attention on miRNAs. However, other molecular components of the exosome cargo could contribute to the angiogenic effects of exosomes as well as representing potential biomarkers. Fourth, and conversely, extracellular miRNAs are not only contained in exosomes, and the fundamental and translational importance of miRNAs carried via different transporter systems is not to be neglected.

MATERIALS AND METHODS

Clinical Sample Collection and Processing

The collection and use of clinical samples for research complied with the ethical principles stated in the "Declaration of Helsinki" and the Human Tissue Act and were covered by ethical approvals from the UK National Research Ethic Service NRES (Research Ethic Committee –REC- references 13/OL/1687 and 10/HO107/63). We collected leftover samples from patients undergoing aortic valve replacement (AVR). The total PF volume was collected immediately after opening of the pericardium using a 20 mL syringe and transported to the laboratory in a sterile 50 mL container. Peripheral blood was collected from an arterial line in citrate-containing vacutainers (BD). Samples of right atrial appendage (RAA) were collected just before cannulation of the right atrium, and samples of ascending thoracic aorta were collected from excessive tissue on closure of the aortotomy. These were immediately placed in RNA Later stabilizing solution (Thermo Fisher Scientific) and stored at −80°C until processed. Blood and PF were processed immediately after collection, as follows. To obtain plasma, the citrate-containing vacutainers were centrifuged at 1,500 × *g*, 4°C for 15 min, and the supernatant was collected. The supernatant underwent further centrifugation at 13,500 × *g*, room temperature (RT), for 5 min to deplete the sample of miRNA-rich platelets. The platelet-free fluid was centrifuged at 13,500 × *g*, RT,

for 5 min to remove any remaining of cells. The final platelet-poor plasma, PF and tissue samples were stored at −80°C until required.

RNA Extraction and Quantitative Real-Time Analysis

Total RNA was extracted using the miRNeasy kit (Qiagen), according to the manufacturer's instructions. For RNA extraction from solid tissues (ascending thoracic aorta and right atrial appendage), about 50 mg of tissue was first homogenized in 1 mL QIAzol (Qiagen) in a gentleMACS M tube using the gentleMACS dissociator (both from Miltenyi Biotec). For RNA extraction from PF and plasma, 200 μl of sample was used with 1 mL of QIAzol. A synthetic analog of the non-human *Caenorhabditis elegans* microRNA-39 (cel-miRNA-39; Qiagen) was spiked in (10 μl of a 5 fmol/μl stock) to normalize RNA extraction efficiency. Reverse transcription of individual miRNAs was performed using the TaqMan miRNA Reverse Transcription Kit and miRNA-specific stem-loop primers (see Table S3; Thermo Fisher Scientific). qPCR was performed in triplicate using 2x Universal PCR Master Mix with No AmpErase UNG (Thermo Fisher Scientific) using the QuantStudio 6 Flex Real-Time PCR System (Thermo Fisher Scientific). miRNA expression was normalized with either cel-miRNA-39 (for biological fluids) or with the small nuclear U6 small nuclear RNA (snRNA) (ID: 001973) (for solid tissues). For mRNA analysis, cDNA obtained using the High-Capacity RNA-to-cDNA Kit (Thermo Fisher Scientific) was amplified by real-time qPCR. TaqMan Gene Expression Assays (Thermo Fisher Scientific) and 2x Universal PCR Master Mix with No AmpErase UNG (Thermo Fisher Scientific) were used to analyze the gene expression of TGFBR1 (ID: Hs00610320_m1), CASPASE3 (ID: Hs00234387_m1), LOX (ID: Hs00942480_m1), and UBC (ID: Hs00824723_m1) (all from Thermo Fisher Scientific). Real-time quantification to measure gene expression for human DICER and mouse TGFBR1 was performed using Power SYBR Green PCR Master Mix (Thermo Fisher Scientific) and normalized against human or mouse GAPDH.

Primers used were as follows:

Human DICER Fw: ATTCTAGTGCAGGTTTTTCAAGCC,

Human DICER Rv: ACCTCAGATTCCACACTTTCCTG,

Human GAPDH Fw: AGCCGCATCTTCTTTTGCGT,

Human GAPDH Rv: TGACGAACATGGGGCATCA,

Mouse TGFBR1 Fw: AGAGCTGTGAGGCCTTGAGA

Mouse TGFBR1 Rv: TTGATGCCTTCCTGTTGGCT

Mouse GAPDH Fw: TGTGAACGGATTTGGCCGTA

Mouse GAPDH Rv: ACTGTGCCGTTGAATTTGCC.

For absolute miRNA quantification, the Ct value obtained from a dilution series (ranging from 100 nM down to 10 fM) of chemically synthesized RNA oligonucleotides corresponding to the mature miRNA sequence of let-7b-5p (UGAGGUAGUAGGUUGUGUG GUU) and miRNA-122-5p (UGGAGUGUGACAAUGGUGUUUG) were used to generate standard curves (both were purchased from Sigma).

miRNA Array on Human Pericardial Fluid

Total RNA was converted to cDNA using a reverse transcription kit (Universal cDNA Synthesis Kit, Exiqon). Three (unpooled) PF samples of the AVR surgical patient group were randomly selected to be run in a PCR-based miRNA array enabling the profiling of 752 human miRNAs (miRNACURY LNA microRNA PCR human panels I and II; version 3, Exiqon). The miRNA array plates were run using a LightCycler 480 (Roche).

miRNA Array Bioinformatics Analyses

For the bioinformatics analyses, the processing settings were as follows: (1) detection scoring: miRNAs not detectable in all three samples or Ct $\geq$ 37 in at least two patients were not considered for future calculations; (2) the average of inter-plate calibrator (UniSp3 IPC) was calculated for each run (representing one sample), and the median was subtracted from each miRNA's Ct; and (3) expression of each miRNA was derived using the $2^{-\Delta\Delta CT}$ method.[41] On the basis of these criteria, array data were inspected using the NormFinder algorithm to assess the variance in expression levels.[42] The best normalizer was found to be the average of assays detected in all three AVR samples; therefore this was used to normalize the array. Data are available at Gene Expression Omnibus (GEO: GSE80577).

PF and Plasma Nanoparticle Profiling

EVs present in whole plasma or PF were characterized using Nanoparticle Tracking Analysis (NTA). One microliter of sample was diluted in sterile water to obtain a suitable concentration to be read, according to the manufacturer's guidelines. The sample was passed through the flow cell. Once the temperature of the flow cell had stabilized at 25°C, six 30 s videos of each sample were taken with a high-resolution camera, with a 1 s pause between each. The videos were then processed by Nanoparticle Tracking Analysis software (version 2.3), giving the concentration of particles per ml for each nanometer size. The final data for each sample is an average of the data from six videos.

Exosome Enrichment from the PF and Plasma

Exosomes were enriched from 250 μl of PF and plasma using the ExoQuick kit (System Biosciences). Thrombin (2.5 μl) (500 U/mL, System Biosciences) per 250 μl plasma was added to remove the fibrin proteins. The samples were incubated at RT for 15 min while mixing, then centrifuged at 10,000 × *g* for 5 min at RT. PF and fibrin-depleted plasma were then filtered through a sterile 0.22 μm filter (Merck Millipore) into a fresh tube, and 75 μl ExoQuick solution was added. The samples were incubated for 30 min at 4°C, then centrifuged for 30 min at 1,500 × *g* and 4°C. The supernatant was removed, and following an additional centrifugation of the sample at 1,500 × *g* for 5 min at 4°C, the fluid was taken off and the pellet was re-suspended in 100 μl of sterile PBS. At the end of the process, the presence of exosomes in the preparation was confirmed by NTA, electron microscopy (see below) and western blotting. Protein concentrations

were determined using Micro BCA protein assay (Thermo Fisher Scientific), and specified exosome doses used in experiments are based on these. miRNAs from exosomes were isolated using the miRNAeasy kit (Qiagen) (see above).

Treatment of Exosomes with Proteinase K and RNase A

For this assay, exosomes obtained according to our method (see above) were treated as previously described.[43] Isolated exosomes were incubated with proteinase K (50 μg/ml; Sigma) for 10 min at 37°C, before 5 mM phenulmethulsulfonyl fluoride (PMSF; Sigma) was added for 10 min at room temperature to inhibit the proteinase K (PK) activity. After the PK inactivation, the samples were incubated with 100 μg/ml RNase A (Thermo Fisher Scientific) for 20 min at 37°C to degrade unprotected RNA, followed by incubation with RiboLock RNase Inhibitor (Thermo Fisher Scientific). Finally protein or RNA was extracted as described earlier. As control samples we included (1) sonicated exosomes followed by treatment with PK and RNase A or (2) exosomes treated with PBS added instead of PK and RNase A.

Electron Microscopy

For morphological studies, each formvar-coated grid was positioned on top of a 5 μl droplet of the pellet containing exosomes, previously fixed for two hours at 4°C in Karnovsky fixative (2% formaldehyde + 2% glutaraldehyde) for 60 min. Afterward, the grid was sequentially positioned on three drops of 0.05 M, pH 7.3 cacodylate buffer for 5 min each. For ultrastructural immunocytochemical study, each formvar-coated grid was positioned on the top of a 5 μl drop of the pellet containing exosomes previously fixed for 2 hr at 4°C in modified Karnovsky fixative (2% formaldehyde + 0.5% glutaraldehyde) for 60 min. The grid was then put on three drops of 0.05 M, pH 7.3 cacodylate buffer for 5 min each, on 2% formaldehyde for 10 min, on three drops of 0.05 M, pH 7.3 cacodylate buffer for 5 min each, on the antibody anti-CD63 (ab23792; Abcam) diluted 1:20 for 60 min, on three drops of 1% BSA in 0.05 M, pH 7.4 Tris buffered saline buffer for 5 min each, on the gold-tagged anti-mouse antibody (Jackson Immunoresearch) diluted 1:20 for 60 min, and on three drops of 0.05 M, pH 7.4 Tris buffered saline buffer for 5 min each. Next, both for morphological and for immunocytochemical study, the grids were placed on a drop of 2% glutaraldehyde for 10 min, then on three drops of distilled water for 5 min each, on 5% uranyl acetate for 3 min, and finally on 0.013% methyl cellulose/0.04% uranyl acetate for 10 min on ice (Lasser). Between each transfer, the excess liquid was removed by holding an absorbing paper close to the side of the grid.

PF Exosome Incorporation by ECs

PF-derived exosomes were labeled using Exo-Glow based on carboxyfluorescein succinimidyl diacetate ester (CFSE) chemistry (System Biosciences) according to the manufacturer's recommendations. Human umbilical vein ECs (HUVECs) (Lonza) were seeded at a density of 5×10^4 cells/well on a 24-well plate coverslip, and 10 μg/ml of labeled PF-derived exosomes were added to target ECs in culture for 24 hr at 37°C. Cells were washed twice with PBS and fixed with 4% buffered PFA (Sigma) in PBS for 20 min at RT. Nuclei were stained by DAPI staining while actin filaments were labeled using Rhodamine Phalloidin (Thermo Fisher Scientific). To assess the uptake of exosomes by ECs, confocal images were acquired with a Leica SP5-AOBS confocal laser scanning microscope attached to a Leica DM I6000 inverted epifluorescence microscope. All images were collected using a 63× NA 1.4 oil immersion lens objective. The excitation signals for Exo-Glow and Rhodamine Phalloidin were 494 and 540 nm, respectively. The fluorescence emitted from the cells was recorded at 521 nm for Exo-Glow and 565 nm for Rhodamine Phalloidin. In all cases, z-stack images were obtained covering the entire cell volume. Three-dimensional reconstruction of the confocal image z stacks confirmed the cytoplasmatic localization of internalized exosomes.

Cell Culture and Cell Biology

HUVECs (Lonza) were grown in endothelial cell basal medium, EBM-2 (Lonza), with the addition of 2% FBS and SingleQuots Kit (EGM-2 medium; Lonza) at 37°C with 5% CO_2. After the first expansion, cells were then grown in EGM-2 medium using 2% exosome-depleted FBS (System Biosciences). To mimic ischemia in vitro, ECs were exposed to hypoxia (1% pO_2) for 24 hr followed by treatment with different concentrations of PF/plasma-derived exosomes or exosome-depleted PF/plasma for 24 hr. HUVECs were used between passages 2 and 6.

Evaluation of Apoptosis and BrdU Incorporation in ECs

HUVECs were seeded in 96-well plates (5×10^3/well) and treated with 5, 10, or 20 μg/ml of PF exosomes or the same concentrations of exosome-depleted PF for 24 hr in hypoxia (1% pO_2). The medium was then replaced with a complete medium with supplemental BrdU (10 μM) for 24 hr. BrdU incorporation was measured using the BrdU ELISA assay kit (Roche). Caspase-3/7 activity was measured at 24 hr using a luminescent cell death detection kit according to the manufacturer's instructions (Caspase-GLO assay; Promega).

In Vitro Angiogenesis

HUVECs were seeded in 6-well plates (2×10^5/well) and treated with 10 μg/ml of PF/plasma exosomes or exosome-depleted PF/plasma for 24 hr in hypoxia, then detached using Accutase (Innovative Cell Technologies) and plated (10^4/well) in a flat-bottom 96-well plate or μ-Slide Angiogenesis (Ibidi), coated with Growth Factor Reduced Matrigel (Corning). For *mir*Vana miRNA mimic or *mir*Vana miRNA inhibitor experiments, HUVECs were seeded at a concentration of 7×10^3/well. After 6 hr, HUVECs were stained with Calcein AM (Biotium), and network formation was quantified by calculating the length of the cellular network on images captured using an objective magnification of 5× or by Angiogenesis Analyzer for ImageJ using a fluorescence objective magnification of 2.5×.

HUVEC and Exosome Transfection

Lipofectamine RNAiMAX (Thermo Fisher Scientific) was used to transfect HUVECs with scramble siRNA (75 nM total), siRNAs against DICER (25 nM for each siRNA, 75 nM total), *mir*Vana miRNA mimic let-7b-5p (12.5 nM, MC11050), and *mir*Vana miRNA

inhibitor let-7b-5p (12.5 nM, MH11050), Pre-miR Negative Control (12.5 nM, AM17120) and Anti-miR Negative Control (12.5 nM, AM17011) (all from Thermo Fisher Scientific), according to the manufacturer's instructions. Published sequences[22,44] of siRNA against DICER and scramble were used (all purchased from Qiagen).

Exosomes were transfected with *mir*Vana miRNA inhibitor let-7b-5p (MH11050; Thermo Fisher Scientific) using Exo-Fect Exosome Transfection Kit (System Biosciences) and following the guideline's recommendations.

In Vivo Experiments

The experiments involving mice were performed in accordance with the Animal (Scientific Procedures) Act (UK) of 1986 prepared by the Institute of Laboratory Animal Resources and under the auspices of UK Home Office Project and personal licenses. Eight-week-old CD1 male mice underwent surgical induction of unilateral limb ischemia (LI) by performing occlusion of the left femoral artery, as we reported previously.[45] Immediately after LI induction, mice received 100 μg of exosomes derived from either PF or plasma into the ischemic adductor muscle, while a control group received PBS (n = 20 mice/group). The superficial blood flow to both ischemic and non-ischemic feet was measured using a high-resolution laser color Doppler imaging system (Moor LDI2, Moor Instruments) at days 0 and 7 after induction of limb ischemia. Blood flow recovery was calculated as a percentage versus day 0 of the control PBS. At day 7, mice under terminal anesthesia were perfusion-fixed successively with 6 mL of 0.05 M EDTA and 10 mL of 10% formalin solution. Limb muscles were harvested and stored in PFA 4% overnight at room temperature then washed with PBS and finally treated with 30% sucrose overnight at 4°C. The tissue samples were then embedded in optical cutting temperature (OCT) compound and stored at −80°C until histological and immunohistochemical analyses. A second set of mice (n = 12 per group) were made ischemic and immediately treated with either PF exosomes or PBS as described above. Doppler analyses were performed at baseline, 7, 14, and 21 days post-ischemia induction. For molecular biology analyses, mice were sacrificed at 3 days after surgery, and the ischemic abductor muscles were dissected and snap-frozen in liquid nitrogen. Total RNA was extracted from snap-frozen muscles as described above.

Histology

The experiments involving mice were performed in accordance with the Animal (Scientific Procedures) Act (UK) 1986 prepared by the Institute of Laboratory Animal Resources and covered under the UK Home Office Project license PPL/30/3373. The functional impact of PF- and plasma-derived exosomes on treatment of CD1-ischemic mice was assessed by measuring capillary density in the adductor muscle. Eight-micrometer-thick muscle sections were stained using biotin-conjugated Isolectin B4 (from Griffonia Simplicifolia; Thermo Fisher Scientific) and streptavidin-conjugated Alexa 488 (Thermo Fisher Scientific) antibodies to detect capillaries. Nuclei were stained with DAPI (4′,6-diamidino-2-phenylindole). The slides were mounted using Fluoromount-G (eBioscience). The relative number of positive cells was counted in eight randomly selected high-power fields (magnification 20×) using a Zeiss inverted fluorescence microscope. Analyses were performed using muscles from eight mice per group. Capillary density was expressed as number per square millimeter.

Immunoprecipitation and Western Blot Analysis

One hundred microliters of Magna Bind goat anti-mouse IgG Magnetic Beads (Thermo Fisher Scientific) were washed three times with PBS solution (200 μl) and incubated with 10 μg of mouse monoclonal anti-AGO2 (ab57113, Abcam) or mouse IgG (Santa Cruz Biotechnology) antibodies for 2 hr at 4°C. The pre-incubated beads and antibodies were then added to 200 μL of PF and incubated overnight at 4°C. Beads were washed 3 times with 1% Nonidet P-40 buffer and re-suspended in 200 μL of PBS. One half of each sample was eluted in loading buffer followed by western blot analysis. Protein extracted from HUVECs was used as a positive control. The other half was eluted in 750 μL of QIAzol and processed for RNA isolation and miRNA detection (see above). Exosomes and cells were lysed with RIPA buffer (Santa Cruz Biotechnology) with an added protease inhibitor cocktail. Samples were centrifuged at 14,000 × *g* for 15 min at 4°C, and the supernatant fractions were used for western blot. Western blot was performed as previously described.[46] The following antibodies were used: Alix (Millipore, ABC40; 1:1,000; Merk Millipore), Flotillin-1 (BD, 610820, 1:1,000), EPCAM (Cell Signaling Technology, 2626; 1:1,000), CD63 (Abcam, ab59479; 1:1,000), AGO2 (Abcam, ab57113; 1:1,000), DICER (Abcam, ab14601, 1:500), β-Actin (Sigma, A5441; 1:50,000), ECL Mouse IgG (GE Healthcare, NA931; 1:2,000), and ECL Rabbit IgG (GE Healthcare, NA934; 1:2,000).

Statistical Analysis

Comparisons between two different conditions were assessed using the two-tailed Student's t test. If the normality test failed, the Mann-Whitney test was performed. Experiments with three or more experimental groups were compared using one-way ANOVA with either a post hoc Dunnett's or Tukey's multi-comparison test, as appropriate. Toe survival was tested using log rank analysis. The data are expressed as mean ± SEM. p values less than 0.05 were considered to indicate statistical significance (*$p < 0.05$, **$p < 0.01$, ***$p \leq 0.001$). Analyses were performed using Prism software version 6 (Graph Pad).

SUPPLEMENTAL INFORMATION

Supplemental Information includes ten figures, three tables, raw data, and full unedited gel and can be found with this article online at http://dx.doi.org/10.1016/j.ymthe.2016.12.022.

AUTHOR CONTRIBUTIONS

C.B. designed and performed experiments, analyzed data, and wrote the manuscript; M.B., S.S., A.I.U.S., C.R., G.S., and F.S. performed experiments and analyzed data; E.P. analyzed data and revised the manuscript; C.R. collected clinical samples under ethical approval and revised the manuscript; G.D.A. collected clinical samples under

ethical approval, revised the manuscript, and obtained funds for the research; C.E. designed the study and the experiments, wrote the manuscript, and obtained funds for the research. All authors approved the final manuscript.

CONFLICTS OF INTEREST

C.E. and G.D.A. are inventors on a (pending) priority patent application (No. 1505747.4; Title: Exosomes) filed by the University of Bristol. The authors have no other potential competing interest to disclose.

ACKNOWLEDGMENTS

This study was supported by the British Heart Foundation (BHF) program grant "MicroRNAs from Cardiac Surgery to Basic Science—and Back?" (RG/15/5/31446) and awards from the BHF Regenerative Medicine Centers (RM/13/2/30158) and the Leducq Transatlantic Network MIRVAD (13 CVD 02) (all to C.E.). Moreover, we received support from the National Institute of Heath Research (NIHR) through the Bristol Biomedical Research Unit (BRU) in Cardiovascular Medicine (to G.D.A.). The views expressed are those of the authors and not necessarily those of the NHS, the NIHR, or the Department of Health. We are grateful to Giovanni Biglino and Paolo Madeddu (both from the University of Bristol) for statistical analyses and critical revision of the manuscript, respectively. Giovanna Finzi (Circolo Varese Hospital) provided technical support with electron microscopy images. We acknowledge the Wolfson Bioimaging Facility (Dominic Alibhai) for the support with the confocal images.

REFERENCES

1. Shabetai, R. (2012). The Pericardium (Springer Science & Business Media), 1 pp.
2. Masters, M., and Riley, P.R. (2014). The epicardium signals the way towards heart regeneration. Stem Cell Res. (Amst.) *13* (3 Pt B), 683–692.
3. Page, E., Upshaw-Earley, J., and Goings, G. (1992). Permeability of rat atrial endocardium, epicardium, and myocardium to large molecules. Stretch-dependent effects. Circ. Res. *71*, 159–173.
4. Blair, D.M. (1925). The Lymphatics of the Heart, 1 pp.
5. Mehlhorn, U., Geissler, H.J., Laine, G.A., and Allen, S.J. (2001). Myocardial fluid balance. Eur. J. Cardiothorac. Surg. *20*, 1220–1230.
6. Stewart, R.H., Rohn, D.A., Allen, S.J., and Laine, G.A. (1997). Basic determinants of epicardial transudation. Am. J. Physiol. *273*, H1408–H1414.
7. Weihrauch, D., Tessmer, J., Warltier, D.C., and Chilian, W.M. (1998). Repetitive coronary artery occlusions induce release of growth factors into the myocardial interstitium. Am. J. Physiol. *275*, H969–H976.
8. Pérez-Cárceles, M.D., Noguera, J., Jiménez, J.L., Martínez, P., Luna, A., and Osuna, E. (2004). Diagnostic efficacy of biochemical markers in diagnosis post-mortem of ischaemic heart disease. Forensic Sci. Int. *142*, 1–7.
9. Osuna, E., Pérez-Cárceles, M.D., Alvarez, M.V., Noguera, J., and Luna, A. (1998). Cardiac troponin I (cTn I) and the postmortem diagnosis of myocardial infarction. Int. J. Legal Med. *111*, 173–176.
10. Kuosmanen, S.M., Hartikainen, J., Hippeläinen, M., Kokki, H., Levonen, A.-L., and Tavi, P. (2015). MicroRNA profiling of pericardial fluid samples from patients with heart failure. PLoS ONE *10*, e0119646.
11. Condorelli, G., Latronico, M.V.G., and Cavarretta, E. (2014). microRNAs in cardiovascular diseases: current knowledge and the road ahead. J. Am. Coll. Cardiol. *63*, 2177–2187.
12. Chen, X., Ba, Y., Ma, L., Cai, X., Yin, Y., Wang, K., Guo, J., Zhang, Y., Chen, J., Guo, X., et al. (2008). Characterization of microRNAs in serum: a novel class of biomarkers for diagnosis of cancer and other diseases. Cell Res. *18*, 997–1006.
13. Emanueli, C., Shearn, A.I.U., Angelini, G.D., and Sahoo, S. (2015). Exosomes and exosomal miRNAs in cardiovascular protection and repair. Vascul. Pharmacol. *71*, 24–30.
14. Bang, C., Batkai, S., Dangwal, S., Gupta, S.K., Foinquinos, A., Holzmann, A., Just, A., Remke, J., Zimmer, K., Zeug, A., et al. (2014). Cardiac fibroblast-derived microRNA passenger strand-enriched exosomes mediate cardiomyocyte hypertrophy. J. Clin. Invest. *124*, 2136–2146.
15. Vicencio, J.M., Yellon, D.M., Sivaraman, V., Das, D., Boi-Doku, C., Arjun, S., Zheng, Y., Riquelme, J.A., Kearney, J., Sharma, V., et al. (2015). Plasma exosomes protect the myocardium from ischemia-reperfusion injury. J. Am. Coll. Cardiol. *65*, 1525–1536.
16. Chi, K.R. (2016). The tumour trail left in blood. Nature *532*, 269–271.
17. Emanueli, C., Shearn, A.I.U., Laftah, A., Fiorentino, F., Reeves, B.C., Beltrami, C., Mumford, A., Clayton, A., Gurney, M., Shantikumar, S., and Angelini, G.D. (2016). Coronary artery-bypass-graft surgery increases the plasma concentration of exosomes carrying a cargo of cardiac microRNAs: an example of exosome trafficking out of the human heart with potential for cardiac biomarker discovery. PLoS ONE *11*, e0154274.
18. Otsuka, M., Zheng, M., Hayashi, M., Lee, J.-D., Yoshino, O., Lin, S., and Han, J. (2008). Impaired microRNA processing causes corpus luteum insufficiency and infertility in mice. J. Clin. Invest. *118*, 1944–1954.
19. Kuehbacher, A., Urbich, C., Zeiher, A.M., and Dimmeler, S. (2007). Role of Dicer and Drosha for endothelial microRNA expression and angiogenesis. Circ. Res. *101*, 59–68.
20. Ota, T., Fujii, M., Sugizaki, T., Ishii, M., Miyazawa, K., Aburatani, H., and Miyazono, K. (2002). Targets of transcriptional regulation by two distinct type I receptors for transforming growth factor-beta in human umbilical vein endothelial cells. J. Cell. Physiol. *193*, 299–318.
21. Castañares, C., Redondo-Horcajo, M., Magán-Marchal, N., ten Dijke, P., Lamas, S., and Rodríguez-Pascual, F. (2007). Signaling by ALK5 mediates TGF-beta-induced ET-1 expression in endothelial cells: a role for migration and proliferation. J. Cell Sci. *120*, 1256–1266.
22. Suárez, Y., Fernández-Hernando, C., Yu, J., Gerber, S.A., Harrison, K.D., Pober, J.S., Iruela-Arispe, M.L., Merkenschlager, M., and Sessa, W.C. (2008). Dicer-dependent endothelial microRNAs are necessary for postnatal angiogenesis. Proc. Natl. Acad. Sci. U S A *105*, 14082–14087.
23. Sahoo, S., and Losordo, D.W. (2014). Exosomes and cardiac repair after myocardial infarction. Circ. Res. *114*, 333–344.
24. Barile, L., Lionetti, V., Cervio, E., Matteucci, M., Gherghiceanu, M., Popescu, L.M., Torre, T., Siclari, F., Moccetti, T., and Vassalli, G. (2014). Extracellular vesicles from human cardiac progenitor cells inhibit cardiomyocyte apoptosis and improve cardiac function after myocardial infarction. Cardiovasc. Res. *103*, 530–541.
25. Beltrami, A.P., Barlucchi, L., Torella, D., Baker, M., Limana, F., Chimenti, S., Kasahara, H., Rota, M., Musso, E., Urbanek, K., et al. (2003). Adult cardiac stem cells are multipotent and support myocardial regeneration. Cell *114*, 763–776.
26. van Berlo, J.H., Kanisicak, O., Maillet, M., Vagnozzi, R.J., Karch, J., Lin, S.-C.J., Middleton, R.C., Marbán, E., and Molkentin, J.D. (2014). c-kit+ cells minimally contribute cardiomyocytes to the heart. Nature *509*, 337–341.
27. Keith, M.C.L., and Bolli, R. (2015). "String theory" of c-kit(pos) cardiac cells: a new paradigm regarding the nature of these cells that may reconcile apparently discrepant results. Circ. Res. *116*, 1216–1230.
28. Sahoo, S., Klychko, E., Thorne, T., Misener, S., Schultz, K.M., Millay, M., Ito, A., Liu, T., Kamide, C., Agrawal, H., et al. (2011). Exosomes from human CD34(+) stem cells mediate their proangiogenic paracrine activity. Circ. Res. *109*, 724–728.
29. Wang, X., Huang, W., Liu, G., Cai, W., Millard, R.W., Wang, Y., Chang, J., Peng, T., and Fan, G.C. (2014). Cardiomyocytes mediate anti-angiogenesis in type 2 diabetic rats through the exosomal transfer of miR-320 into endothelial cells. J. Mol. Cell. Cardiol. *74*, 139–150.
30. Kamo, T., Akazawa, H., and Komuro, I. (2015). Cardiac nonmyocytes in the hub of cardiac hypertrophy. Circ. Res. *117*, 89–98.

31. Heineke, J., Auger-Messier, M., Xu, J., Oka, T., Sargent, M.A., York, A., Klevitsky, R., Vaikunth, S., Duncan, S.A., Aronow, B.J., et al. (2007). Cardiomyocyte GATA4 functions as a stress-responsive regulator of angiogenesis in the murine heart. J. Clin. Invest. *117*, 3198–3210.

32. Shiojima, I., Sato, K., Izumiya, Y., Schiekofer, S., Ito, M., Liao, R., Colucci, W.S., and Walsh, K. (2005). Disruption of coordinated cardiac hypertrophy and angiogenesis contributes to the transition to heart failure. J. Clin. Invest. *115*, 2108–2118.

33. Krell, J., Stebbing, J., Carissimi, C., Dabrowska, A.F., de Giorgio, A., Frampton, A.E., Harding, V., Fulci, V., Macino, G., Colombo, T., and Castellano, L. (2016). TP53 regulates miRNA association with AGO2 to remodel the miRNA-mRNA interaction network. Genome Res. *26*, 331–341.

34. Cai, X., Janku, F., Zhan, Q., and Fan, J.-B. (2015). Accessing Genetic Information with Liquid Biopsies. Trends Genet. *31*, 564–575.

35. Khan, M., Nickoloff, E., Abramova, T., Johnson, J., Verma, S.K., Krishnamurthy, P., Mackie, A.R., Vaughan, E., Garikipati, V.N., Benedict, C., et al. (2015). Embryonic stem cell-derived exosomes promote endogenous repair mechanisms and enhance cardiac function following myocardial infarction. Circ. Res. *117*, 52–64.

36. Gallet, R., Dawkins, J., Valle, J., Simsolo, E., de Couto, G., Middleton, R., Tseliou, E., Luthringer, D., Kreke, M., Smith, R.R., et al. (2016). Exosomes secreted by cardiosphere-derived cells reduce scarring, attenuate adverse remodelling, and improve function in acute and chronic porcine myocardial infarction. Eur. Heart J., Published online September 27, 2016. http://dx.doi.org/10.1093/eurheartj/ehw240.

37. Benito-Martin, A., Di Giannatale, A., Ceder, S., and Peinado, H. (2015). The new deal: a potential role for secreted vesicles in innate immunity and tumor progression. Front. Immunol. *6*, 66.

38. Lee, C., Mitsialis, S.A., Aslam, M., Vitali, S.H., Vergadi, E., Konstantinou, G., Sdrimas, K., Fernandez-Gonzalez, A., and Kourembanas, S. (2012). Exosomes mediate the cytoprotective action of mesenchymal stromal cells on hypoxia-induced pulmonary hypertension. Circulation *126*, 2601–2611.

39. Om, S.Y., Kim, S.-H., Choi, S.-W., Choi, H.O., Kim, Y.-G., Song, J.-M., Jung, S.H., Kim, D.H., Kang, D.H., Song, J.K., and Shim, T.S. (2014). T cell-based assay of pericardial fluid mononuclear cells for the diagnosis of tuberculous pericardial effusion. J. Am. Coll. Cardiol. *64*, 1966–1968.

40. Fancello, L., Monteil, S., Popgeorgiev, N., Rivet, R., Gouriet, F., Fournier, P.-E., Raoult, D., and Desnues, C. (2014). Viral communities associated with human pericardial fluids in idiopathic pericarditis. PLoS ONE *9*, e93367.

41. Livak, K.J., and Schmittgen, T.D. (2001). Analysis of relative gene expression data using real-time quantitative PCR and the 2(-Delta Delta C(T)) method. Methods *25*, 402–408.

42. Andersen, C.L., Jensen, J.L., and Ørntoft, T.F. (2004). Normalization of real-time quantitative reverse transcription-PCR data: a model-based variance estimation approach to identify genes suited for normalization, applied to bladder and colon cancer data sets. Cancer Res. *64*, 5245–5250.

43. Shelke, G.V., Lässer, C., Gho, Y.S., and Lötvall, J. (2014). Importance of exosome depletion protocols to eliminate functional and RNA-containing extracellular vesicles from fetal bovine serum. J. Extracell. Vesicles *3*, 3.

44. Chendrimada, T.P., Gregory, R.I., Kumaraswamy, E., Norman, J., Cooch, N., Nishikura, K., and Shiekhattar, R. (2005). TRBP recruits the Dicer complex to Ago2 for microRNA processing and gene silencing. Nature *436*, 740–744.

45. Emanueli, C., Salis, M.B., Stacca, T., Gaspa, L., Chao, J., Chao, L., Piana, A., and Madeddu, P. (2001). Rescue of impaired angiogenesis in spontaneously hypertensive rats by intramuscular human tissue kallikrein gene transfer. Hypertension *38*, 136–141.

46. Caporali, A., Meloni, M., Völlenkle, C., Bonci, D., Sala-Newby, G.B., Addis, R., Spinetti, G., Losa, S., Masson, R., Baker, A.H., et al. (2011). Deregulation of microRNA-503 contributes to diabetes mellitus-induced impairment of endothelial function and reparative angiogenesis after limb ischemia. Circulation *123*, 282–291.

YMTHE, Volume *25*

Supplemental Information

Human Pericardial Fluid Contains Exosomes Enriched with Cardiovascular-Expressed MicroRNAs and Promotes Therapeutic Angiogenesis

Cristina Beltrami, Marie Besnier, Saran Shantikumar, Andrew I.U. Shearn, Cha Rajakaruna, Abas Laftah, Fausto Sessa, Gaia Spinetti, Enrico Petretto, Gianni D. Angelini, and Costanza Emanueli

Supplemental Figures

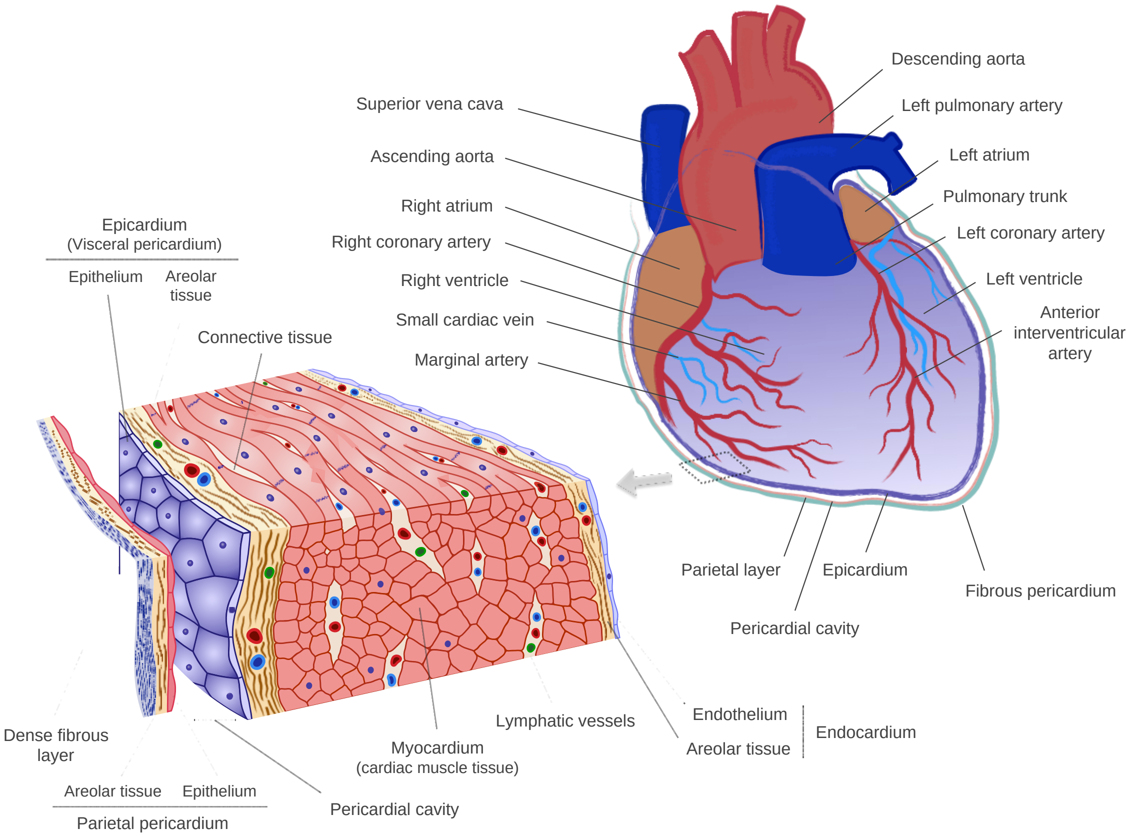

Figure S1. Internal anatomy of the heart. The heart contains three layers: the superficial epicardium; the middle myocardium; and the inner endocardium. The pericardial fluid is contained within the double-walled pericardial sac (also known as pericardium) that surrounds the heart and the roots of the great vessels bringing blood to and from the heart cells, namely the superior and inferior vena cava; pulmonary arteries and pulmonary veins. The pericardium is composed of two layers: 1) the superficial fibrous pericardium, comprised of connective tissue, which is continuous with the tunica adventitia of the great blood vessels and anchors the heart to the surrounding walls; and 2) the serous pericardium composed of mesothelial cells. The serous pericardium is in turn formed by a parietal layer that fuses with the fibrous pericardium and a visceral layer (epicardium) and the epicardium, which sits on and signals to the myocardium. The pericardial fluid (PF) is secreted by the serous membranes and obtained by both capillary permeability and hydrostatic/osmotic pressure from the epicardium and from the interstitial fluid underlying the myocardium.

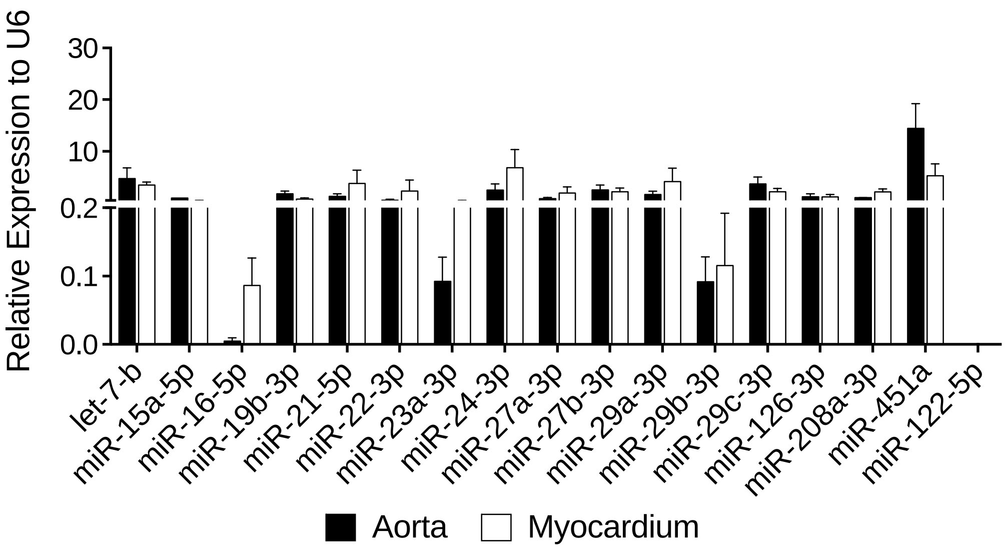

Figure S2. MicroRNA (miRNA) expression in ascending aorta (aorta) and right atrial appendage (myocardium) samples collected from the surgical patients. The cardiovascular expression of selected miRNAs was confirmed by RT-qPCR analyses in the available ascending aorta (n=5-7) and right atrium (n=3-4) clinical samples collected as leftover material from the patients undergoing aortic valve replacement (AVR). U6 was used as the endogenous control. All values are mean + s.e.m.

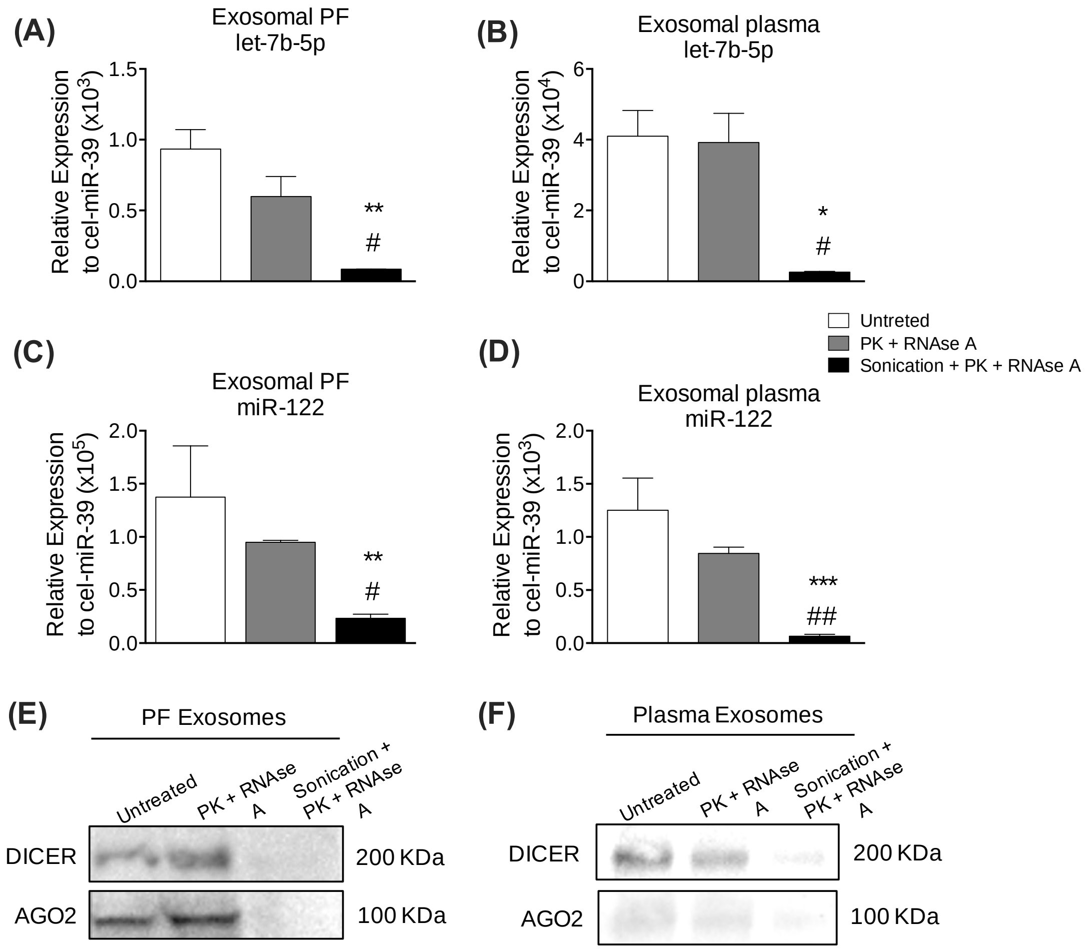

Figure S3. Effect of exosomes treatment with Proteinase K and RNAse A on DICER, AGO-2 and microRNAs. Exosomes enriched from either the pericardial fluid (PF) or plasma were submitted, or not, to sonication to break the exosome membrane. Next, sonicated or intact exosomes were incubated with proteinase K (PK; 50 µg/ml) and RNAse A (100 µg/ml). A control group (Untreated) consisted of non-sonicated exosomes not receiving PK/RNAse A. Exosomal let-7b and miR-122 expression in **(A,C)** PF or **(B,D)** plasma were measured by RT-qPCR using spike-in cel-miR-39 as a normalizer. Representative Western blot images of DICER and AGO-2 protein incorporated in the **(E)** PF and **(F)** plasma exosomes. $^{*}P\leq0.05$, $^{**}P<0.01$ and $^{***}P \leq 0.001$ vs. untreated, $^{\#}P\leq0.05$ and $^{\#\#}P\leq0.01$ vs. treatment with PK and RNAse A. Statistical significance was tested using one-way ANOVA with Tukey's *post hoc* test. All values are mean + s.e.m; n=3.

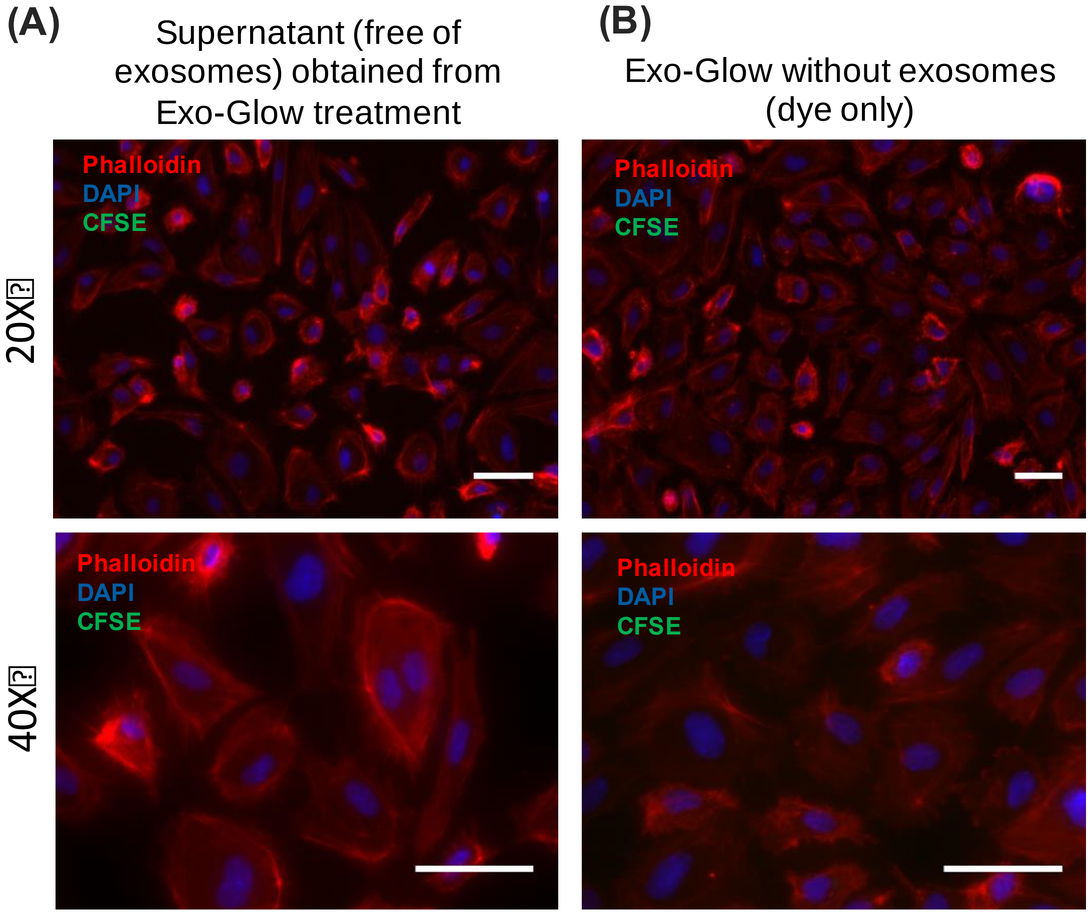

Figure S4. Control for exosome incorporation staining. Endothelial cells (ECs) were cultured for 24hrs with **(A)** supernatant obtained from PF-derived exosomes stained with CFSE (green fluorescence) or **(B)** the CGSE dye used for the exosome staining. Cells were stained with phalloidin (in red) and DAPI (blue). (Scale bar, 25 µm).

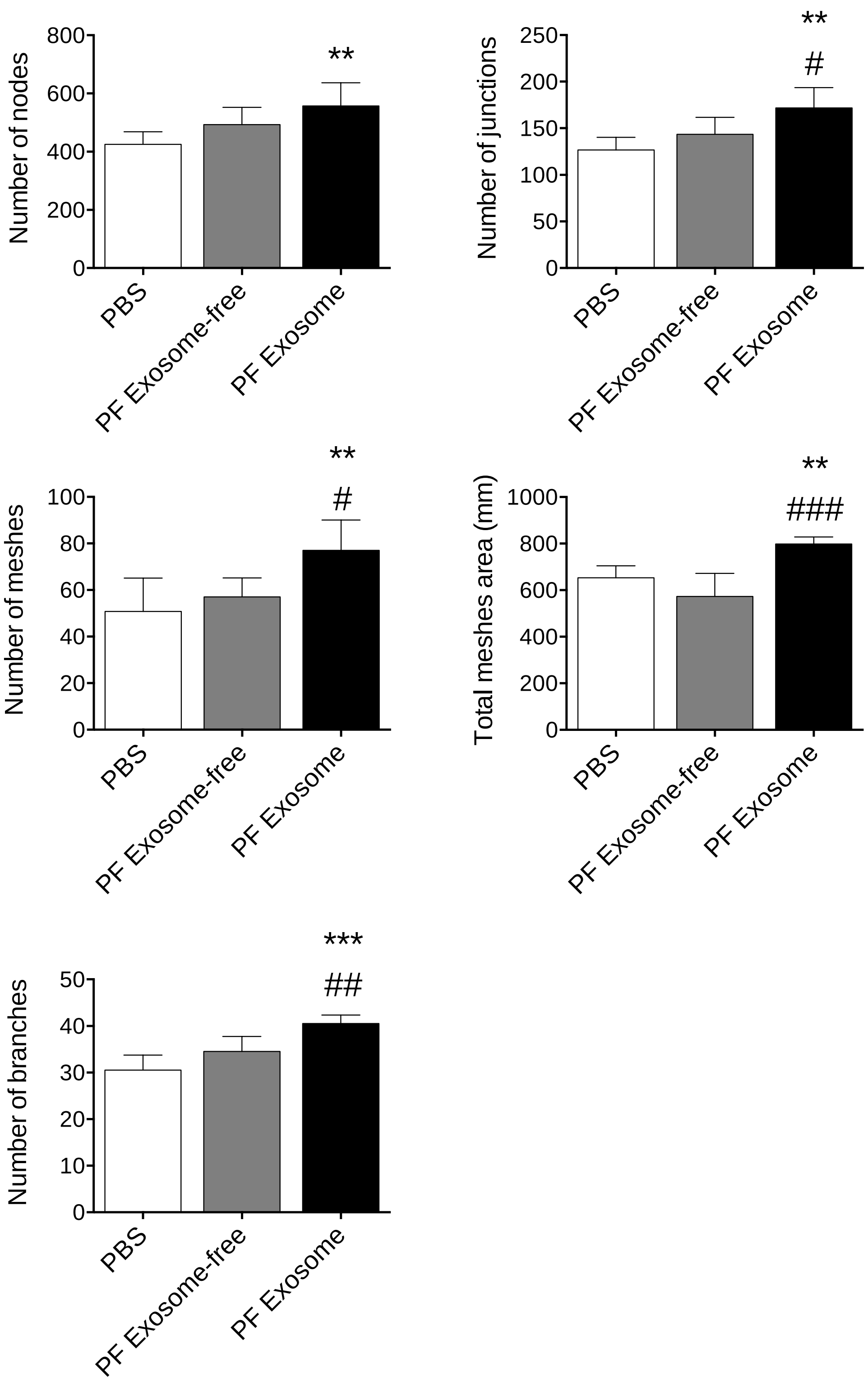

Figure S5. Matrigel assay quantification showing additional parameters to those presented in Fig.5E. In Figure 5E, we present the total length of tube-like structure of the Matrigel assays. Here, we show additional parameters derived from the quantification of the assays. All values are mean + s.e.m. n=5. ** $P \leq 0.01$ and *** $P \leq 0.001$; vs. PBS; $^{\#}P \leq 0.05$; $^{\#\#}P \leq 0.01$ and $^{\#\#\#}P \leq 0.001$; vs. Exosome-free PF.

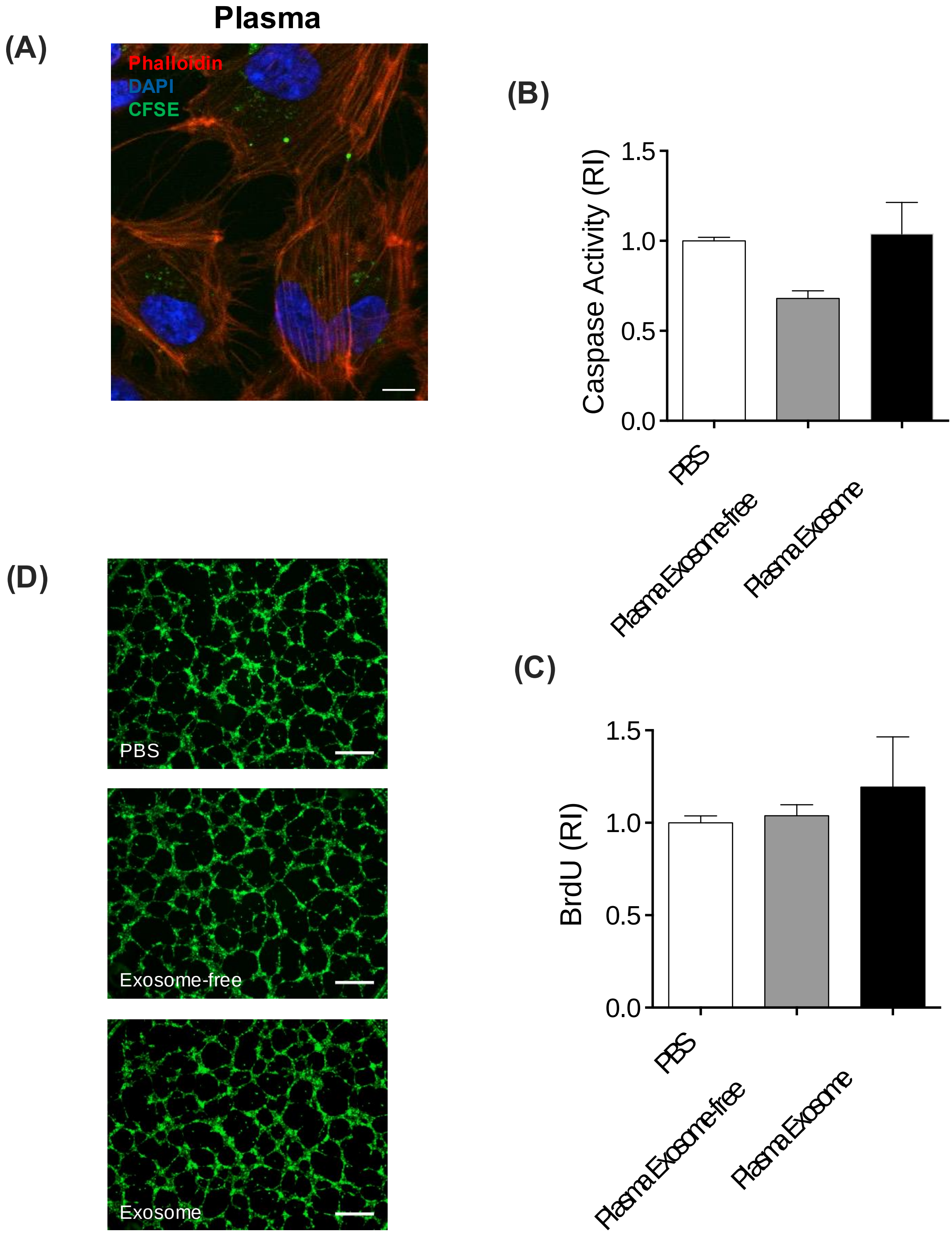
(A)
Plasma
Phalloidin
DAPI
CFSE
(B)
Caspase Activity (RI)
1.5
1.0
0.5
0.0
PBS
Plasma Exosome-free
Plasma Exosome
(C)
BrdU (RI)
1.5
1.0
0.5
0.0
PBS
Plasma Exosome-free
Plasma Exosome
(D)
PBS
Exosome-free
Exosome

(E)

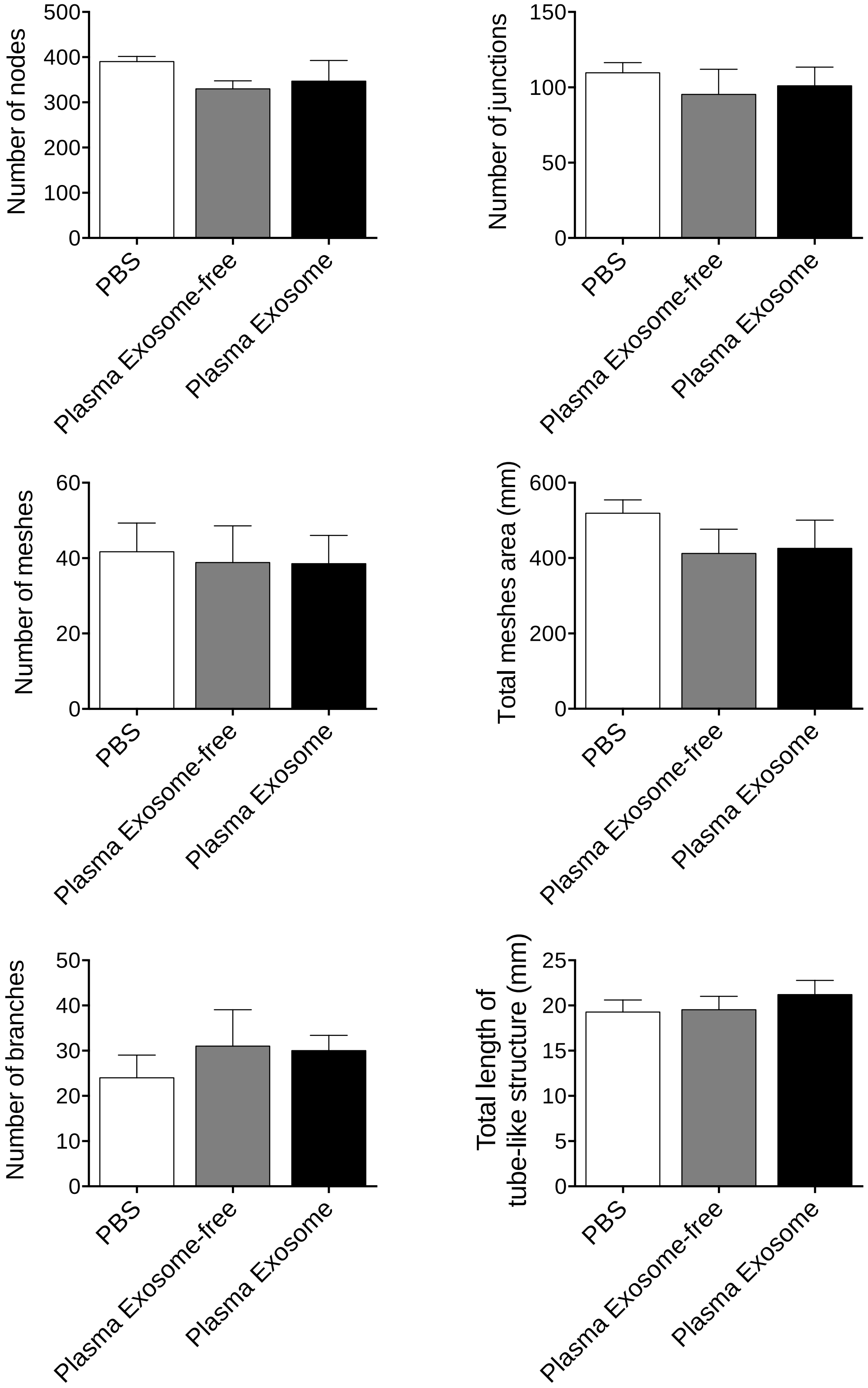

Number of nodes
0
100
200
300
400
500
Number of junctions
0
50
100
150
Number of meshes
0
20
40
60
Total meshes area (mm)
0
200
400
600
Number of branches
0
10
20
30
40
50
Total length of tube-like structure (mm)
0
5
10
15
20
25
PBS
Plasma Exosome-free
Plasma Exosome

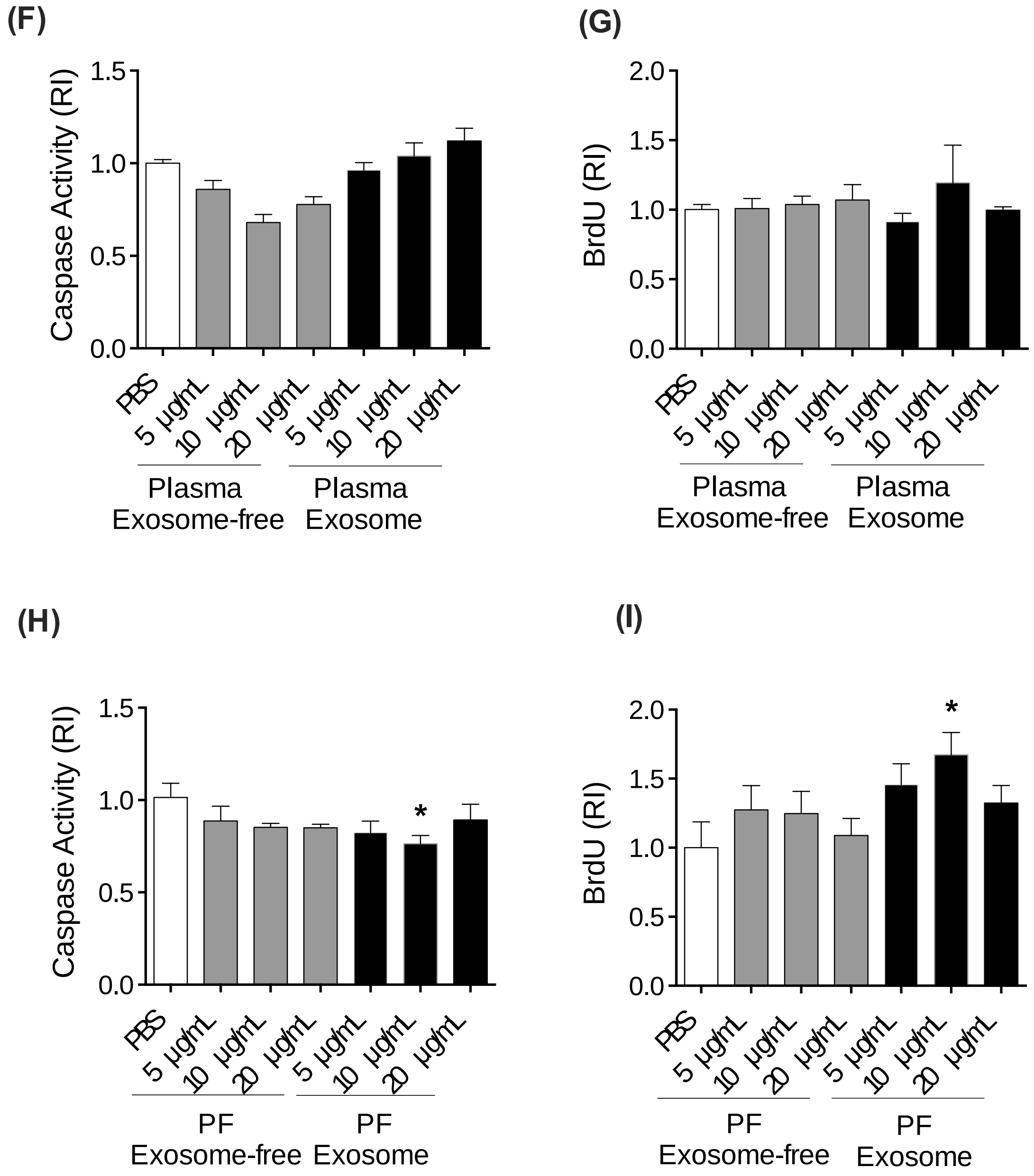

Figure S6. Plasma exosomes are incorporated by cultured ECs, but they do not enhance their angiogenic capacity. (A) Plasma-derived exosomes were stained using CFSE (in green) and incubated (10 µg/ml) with cultured ECs for 24 hrs. Cells were stained with phalloidin (in red) and DAPI (blue) (scale bar, 25 µm). Column graphs show **(B,F)** caspase activity and **(C,G)** BrdU incorporation in ECs treated (24h) with 5, 10 and 20 µg/ml of either plasma-derived exosomes (black columns) or the same concentrations of exosome-depleted plasma. **(D)** Photomicrograph shows the EC network formation on Matrigel (scale bar, 200 µm) while in **(E)** the bar graphs show total length of tube-like structures of ECs treated as previously described; n=5. 2.5X Magnification. In **(H)** and **(I)**, we show the results of apoptosis and proliferation (concentrations-responses) assays in ECs stimulated with PF exosomes. Statistical significance was tested using one-way ANOVA with Dunnett's *post hoc* test. All values are mean+ s.e.m; n=7. *P ≤ 0.05 vs. PBS.

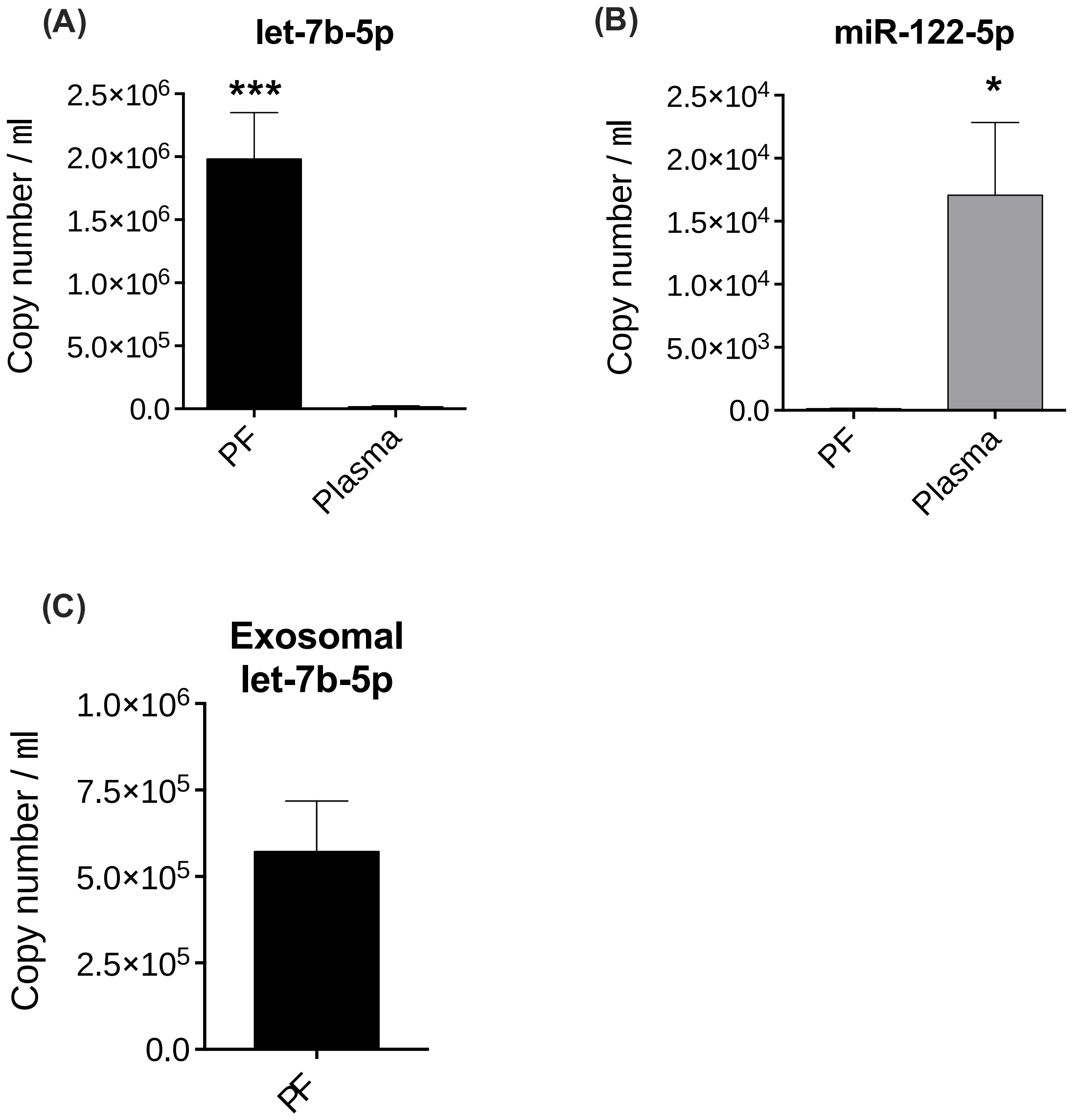

Figure S7. Let-7b-5p expression in total and exosomal PF and plasma samples. Absolute expression of **(A)** let-7b-5p (***P ≤ 0.001 vs plasma) and **(B)** miR-122-5p (*P ≤ 0.05 vs PF) expressed as copy number/µl of fluid was detected in total PF and plasma samples; n=5. **(C)** Copy number/µl of let-7b-5p in the PF exosomes. Unpaired two-tailed Student's *t-test* was applied in (A) and (B). All values are mean + s.e.m.

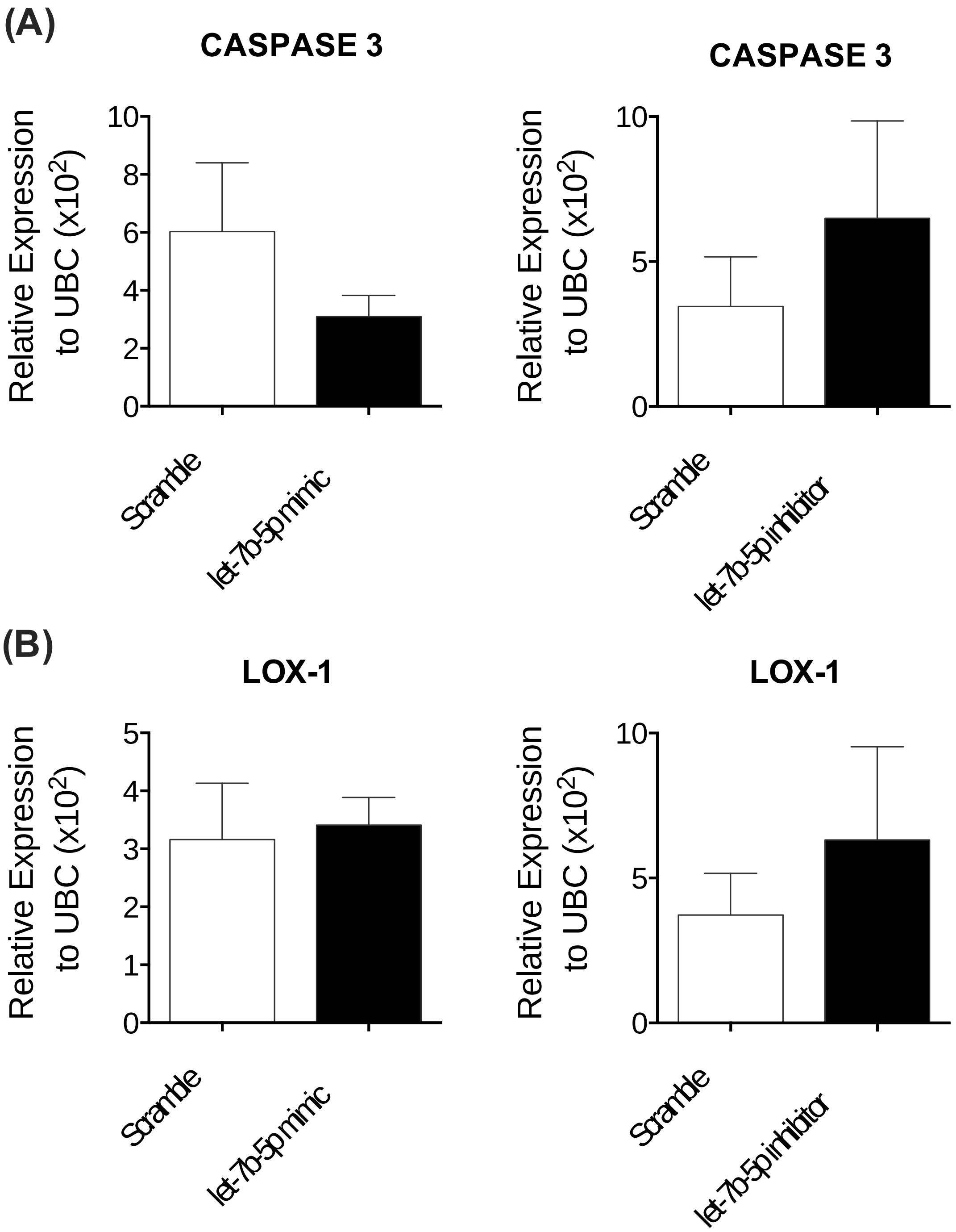

Figure S8. Expression of the previously validated direct target genes of let-7b-5p, *CASPASE-3* and *LOX1*, in ECs after let-7b-5b changes. ECs were transfected with either a mimic of let-7b-5p (right panel) or a let-7b-5p inhibitor (left panel) or the respective scramble sequences. The relative expression of the qPCR is shown using *UBC* as the normalizer. All values are mean + s.e.m; n=4.

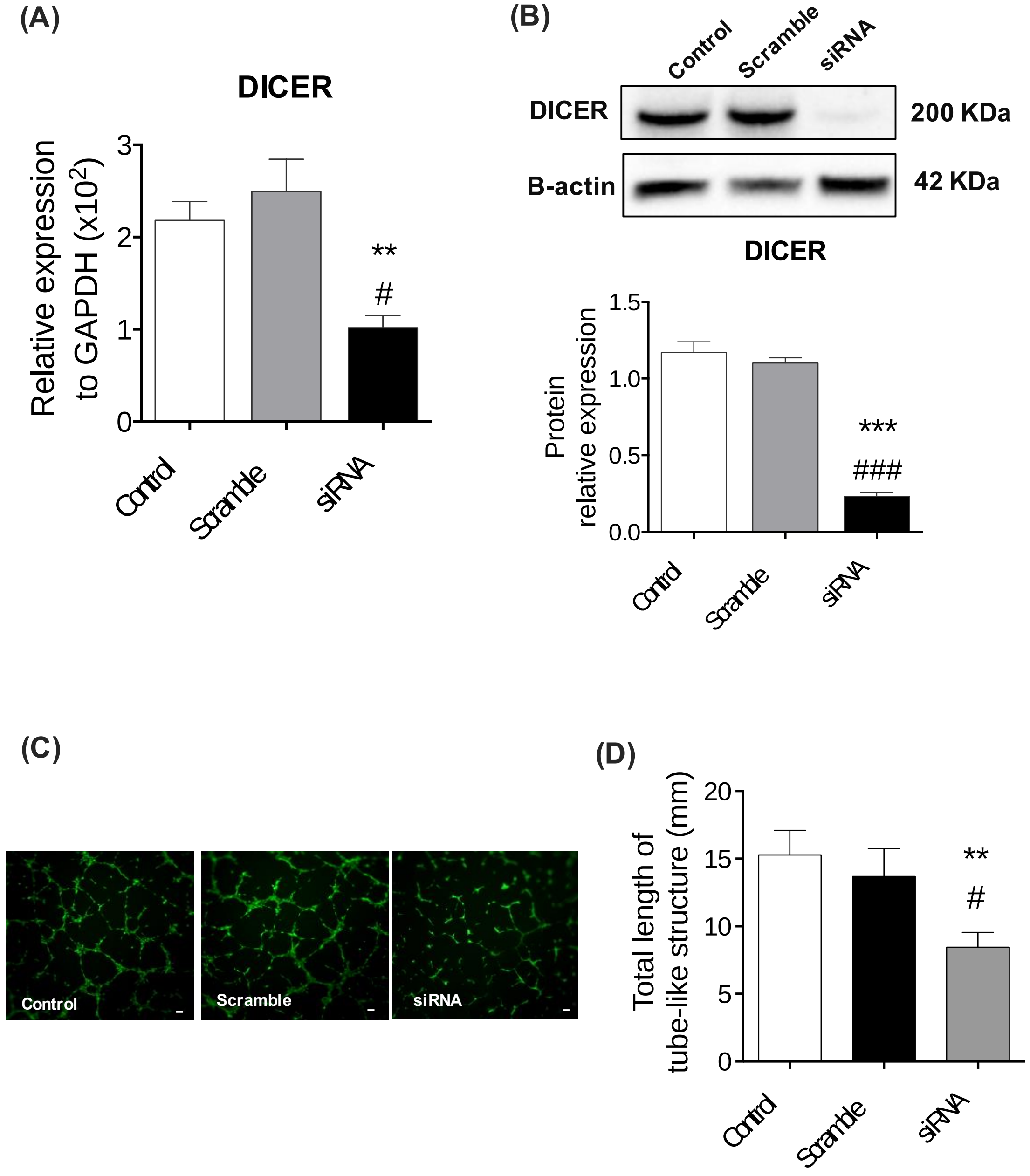

Figure S9. Consequences of Dicer RNA silencing in ECs. ECs were transfected with either Dicer siRNA or a scramble sequence for 24 hrs. Control cells were left untouched. Transfection efficiency was assessed by measuring Dicer mRNA and protein expression by using **(A)** RT–qPCR and **(B)** Western blotting (WB), respectively. An antibody against actin was used as the loading control in the WB. **(C)** Photomicrographs show the endothelial network formation on Matrigel (scale bar 100 µm) and **(D)** bar graphs show the total length of tube-like structures of ECs transfected as previously indicated. Data are presented as mean + s.e.m. from 4 independent experiments. Statistical significance was tested using one-way ANOVA with Dunnett's *post hoc* test. $^{\#}P \leq 0.05$ and $^{\#\#\#}P \leq 0.001$ vs non-transfected control; $^{**}P \leq 0.01$ and $^{***}P \leq 0.001$ vs. scrambles;

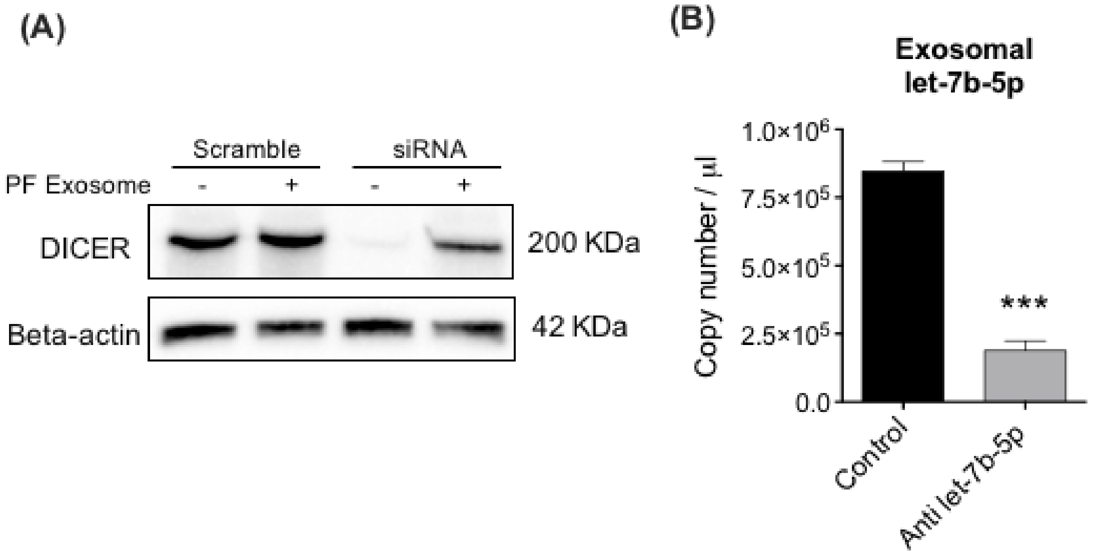

Figure S10. PF exosomes restore Dicer protein levels in ECs after DICER KD, and exosomal let-7b-5p can be inhibited by transfection with anti-let-7b-5p. (A) Western blotting showing the restoration of DICER protein after treatment with PF-derived exosomes (10 µg/ml) of ECs that were transfected with *DICER* siRNA (siRNA) or scramble siRNA (scramble). Antibody against actin was used as loading control; n=3. **(B)** Efficiency of let-7b-5p inhibitor transfection in PF exosomes; n=6. Unpaired two-tailed Student's *t-test* was applied. All values are mean + s.e.m. *** $P \leq 0.001$ vs. control.

Tables

Table S1: Characteristics of the aortic valve replacement (AVR) patients used in the study

(NYHA = New York Heart Association; LVEF = left ventricular ejection fraction)

Characteristic	Total n=16	
Age (Years; mean, SD)		71.7 (7.6)
Sex (males;n,%)		11/16 (68.7%)
Total volume of PF collected (ml; mean, SD)		6.4 (3.8)
Diabetes (n,%)		2/14 (14.2%)
Hypertension (n,%)		14/14 (100%)
Valve stenosis (n,%)		16/16 (100%)
NYHA Class (n,%)	Class 2	14/15 (93.3%)
	Class 3	1/15 (6.7%)
LVEF	Good	14/15 (93.3%)
	Moderate	1/15 (6.6%)

Table S2. List of the microRNAs (miRNAs) expressed in the pericardial fluid (PF) samples.

A PCR-based miRNA array (ID: 203616,Exiqon) was developed using non-pooled pericardial fluid (PF) samples, each taken from a different patient undergoing aortic valve replacement (AVR) surgery. Of the 752 miRNAs present in the array, 359 miRNAs (shown below with their PCR Ct) were expressed by each tested sample. Of these 358 miRNAs, the ones that were already known to be expressed in cardiovascular tissues and cells are in red and additionally underlined when selected for further validation within this study. The particulars of the 16 putative cardiovascular miRNAs selected for further investigations are shown in the Table S3.

PF- sample 1		PF-sample 2		PF-sample 3	
miRNAs	Ct	miRNAs	Ct	miRNAs	Ct
hsa-miR-16-5p	23.12	hsa-miR-451a	16.49	hsa-miR-451a	16.606
hsa-miR-21-5p	23.19	hsa-miR-16-5p	17	hsa-miR-21-5p	20.256
hsa-miR-451a	23.4	hsa-miR-21-5p	17.08	hsa-miR-125b-5p	20.316
hsa-miR-125b-5p	23.58	hsa-miR-125b-5p	17.93	hsa-miR-24-3p	20.386
hsa-miR-23a-3p	24.82	hsa-miR-24-3p	19.12	hsa-miR-19b-3p	20.466
hsa-miR-24-3p	25.08	hsa-miR-29a-3p	19.28	hsa-miR-29a-3p	20.656
hsa-miR-27b-3p	25.41	hsa-miR-29c-3p	19.52	hsa-miR-223-3p	20.846
hsa-let-7b-5p	25.54	hsa-miR-27b-3p	19.55	hsa-miR-22-3p	20.926
hsa-miR-29a-3p	25.86	hsa-miR-22-3p	19.62	hsa-miR-15a-5p	21.036
hsa-miR-19b-3p	26.04	hsa-miR-23a-3p	19.66	hsa-miR-23a-3p	21.176
hsa-miR-29c-3p	26.06	hsa-miR-19b-3p	20.04	hsa-miR-29c-3p	21.186
hsa-miR-23b-3p	26.08	hsa-let-7b-5p	20.06	hsa-miR-99a-5p	21.296
hsa-miR-100-5p	26.28	hsa-miR-15a-5p	20.31	hsa-miR-23b-3p	21.336
hsa-miR-320a	26.3	hsa-miR-23b-3p	20.58	hsa-miR-320b	21.386
hsa-miR-22-3p	26.39	hsa-miR-148a-3p	20.58	hsa-let-7b-5p	21.476
hsa-miR-15a-5p	26.44	hsa-miR-29b-3p	20.72	hsa-miR-27b-3p	21.606
hsa-let-7a-5p	26.49	hsa-miR-34a-5p	20.84	hsa-miR-365a-3p	21.806
hsa-miR-148a-3p	26.56	hsa-miR-99a-5p	20.87	hsa-miR-92a-3p	22.036
hsa-miR-99a-5p	26.78	hsa-miR-320a	21.15	hsa-miR-29b-3p	22.286
hsa-miR-221-3p	26.84	hsa-let-7a-5p	21.22	hsa-miR-140-3p	22.286
hsa-miR-34a-5p	27.11	hsa-miR-221-3p	21.44	hsa-miR-27a-3p	22.396
hsa-miR-92a-3p	27.15	hsa-miR-20a-5p	21.46	hsa-miR-106a-5p	22.396
hsa-miR-378a-3p	27.24	hsa-miR-378a-3p	21.47	hsa-miR-20a-5p	22.526
hsa-miR-150-5p	27.31	hsa-miR-152	21.51	hsa-let-7g-5p	22.556
hsa-miR-125a-5p	27.32	hsa-miR-101-3p	21.6	hsa-miR-130a-3p	22.826
hsa-miR-365a-3p	27.39	hsa-miR-30e-5p	21.66	hsa-miR-151a-5p	22.926
hsa-miR-20a-5p	27.44	hsa-miR-100-5p	21.74	hsa-miR-320a	22.936
hsa-miR-30e-5p	27.59	hsa-miR-27a-3p	21.79	hsa-miR-93-5p	23.276
hsa-miR-423-5p	27.6	hsa-miR-140-3p	21.79	hsa-miR-125a-5p	23.416
hsa-miR-361-5p	27.6	hsa-let-7c	21.88	hsa-miR-342-3p	23.436
hsa-let-7d-3p	27.8	hsa-let-7i-5p	21.89	hsa-miR-103a-3p	23.476
hsa-miR-152	27.81	hsa-miR-93-5p	21.97	hsa-miR-574-3p	23.786
hsa-miR-204-5p	27.84	hsa-miR-92a-3p	21.99	hsa-miR-181a-5p	23.846
hsa-miR-423-3p	27.84	hsa-miR-186-5p	22.01	hsa-miR-423-5p	23.876
hsa-let-7g-5p	27.85	hsa-miR-365a-3p	22.06	hsa-miR-423-3p	23.926
hsa-miR-26a-5p	27.9	hsa-miR-199a-3p	22.13	hsa-miR-532-3p	23.946
hsa-miR-101-3p	27.92	hsa-miR-130a-3p	22.23	hsa-miR-25-3p	24.146
hsa-miR-186-5p	27.95	hsa-miR-223-3p	22.27	hsa-let-7c	24.176
hsa-let-7i-5p	28.01	hsa-miR-106a-5p	22.47	hsa-miR-28-3p	24.336
hsa-miR-27a-3p	28.03	hsa-miR-423-5p	22.48	hsa-miR-99b-5p	24.346
hsa-miR-130a-3p	28.04	hsa-miR-195-5p	22.48	hsa-miR-424-5p	24.406

hsa-miR-99b-5p	28.06	hsa-miR-99b-5p	22.49	hsa-miR-378a-3p	24.426
hsa-let-7c	28.07	hsa-miR-148b-3p	22.49	hsa-miR-191-5p	24.526
hsa-miR-93-5p	28.12	hsa-miR-25-3p	22.58	hsa-miR-26a-5p	24.546
hsa-miR-223-3p	28.16	hsa-let-7g-5p	22.68	hsa-miR-324-3p	24.556
hsa-miR-140-3p	28.18	hsa-miR-125a-5p	22.72	hsa-miR-532-5p	24.606
hsa-miR-30b-5p	28.24	hsa-miR-361-5p	22.76	hsa-miR-15b-5p	24.636
hsa-miR-199a-3p	28.24	hsa-miR-497-5p	22.8	hsa-let-7d-3p	24.796
hsa-miR-195-5p	28.35	hsa-miR-95	22.8	hsa-miR-34a-5p	24.856
hsa-miR-25-3p	28.41	hsa-miR-151a-5p	22.81	hsa-miR-199a-3p	24.876
hsa-miR-29b-3p	28.45	hsa-miR-660-5p	22.83	hsa-miR-30b-5p	24.886
hsa-miR-106a-5p	28.5	hsa-miR-26a-5p	22.93	hsa-miR-101-3p	24.906
hsa-miR-148b-3p	28.53	hsa-miR-335-5p	22.96	hsa-miR-140-5p	25.026
hsa-miR-497-5p	28.6	hsa-miR-335-3p	22.97	hsa-miR-551a	25.076
hsa-miR-342-3p	28.67	hsa-miR-15b-5p	23.06	hsa-miR-151a-3p	25.096
hsa-miR-151a-5p	28.67	hsa-miR-320b	23.06	hsa-miR-497-5p	25.286
hsa-miR-146a-5p	28.74	hsa-miR-140-5p	23.08	hsa-let-7d-5p	25.286
hsa-miR-320b	28.76	hsa-miR-218-5p	23.12	hsa-miR-126-5p	25.376
hsa-miR-126-3p	28.83	hsa-miR-22-5p	23.15	hsa-miR-126-3p	25.416
hsa-miR-30c-5p	28.89	hsa-miR-30a-5p	23.19	hsa-miR-30a-3p	25.426
hsa-miR-103a-3p	28.9	hsa-miR-181a-5p	23.27	hsa-miR-193a-5p	25.466
hsa-miR-30a-5p	29.02	hsa-miR-103a-3p	23.34	hsa-miR-150-5p	25.516
hsa-miR-22-5p	29.06	hsa-miR-30b-5p	23.45	hsa-miR-148b-3p	25.556
hsa-miR-140-5p	29.1	hsa-miR-486-5p	23.49	hsa-miR-221-3p	25.636
hsa-miR-95	29.13	hsa-miR-143-3p	23.6	hsa-miR-22-5p	25.636
hsa-miR-486-5p	29.23	hsa-miR-185-5p	23.67	hsa-miR-146a-5p	25.636
hsa-miR-335-5p	29.25	hsa-miR-106b-5p	23.71	hsa-miR-193b-3p	25.646
hsa-miR-660-5p	29.33	hsa-miR-423-3p	23.75	hsa-miR-30c-5p	25.646
hsa-miR-193a-5p	29.41	hsa-miR-342-3p	23.77	hsa-miR-502-3p	25.706
hsa-miR-574-3p	29.46	hsa-miR-199a-5p	23.82	hsa-let-7i-5p	25.726
hsa-let-7f-5p	29.67	hsa-miR-126-3p	23.86	hsa-miR-152	25.746
hsa-miR-126-5p	29.77	hsa-let-7d-3p	23.86	hsa-miR-200c-3p	25.786
hsa-let-7d-5p	29.8	hsa-miR-146a-5p	23.87	hsa-miR-31-5p	25.806
hsa-miR-532-3p	29.83	hsa-miR-150-5p	23.94	hsa-miR-486-5p	25.826
hsa-miR-200b-3p	29.88	hsa-miR-532-3p	23.94	hsa-miR-95	25.876
hsa-miR-218-5p	30.06	hsa-miR-193a-5p	23.98	hsa-miR-210	25.906
hsa-miR-28-3p	30.06	hsa-miR-574-3p	23.99	hsa-miR-224-3p	25.906
hsa-miR-31-5p	30.11	hsa-miR-324-3p	24.05	hsa-miR-200b-3p	25.926
hsa-miR-181a-5p	30.13	hsa-miR-210	24.18	hsa-miR-374a-5p	25.986
hsa-miR-324-3p	30.14	hsa-miR-424-5p	24.21	hsa-miR-145-5p	26.036
hsa-miR-106b-5p	30.18	hsa-miR-362-3p	24.21	hsa-miR-484	26.066
hsa-miR-185-5p	30.22	hsa-miR-30c-5p	24.22	hsa-miR-31-3p	26.146
hsa-miR-107	30.26	hsa-miR-200b-3p	24.24	hsa-miR-335-5p	26.196
hsa-miR-132-3p	30.31	hsa-miR-199b-5p	24.26	hsa-miR-218-5p	26.206
hsa-miR-200a-3p	30.32	hsa-miR-28-3p	24.28	hsa-miR-132-3p	26.226
hsa-miR-210	30.33	hsa-miR-532-5p	24.44	hsa-miR-16-2-3p	26.246
hsa-miR-151a-3p	30.34	hsa-miR-200a-3p	24.5	hsa-miR-378a-5p	26.256
hsa-miR-10b-5p	30.36	hsa-miR-193b-3p	24.52	hsa-miR-455-3p	26.366
hsa-miR-425-5p	30.39	hsa-miR-324-5p	24.56	hsa-miR-181c-5p	26.446
hsa-miR-424-5p	30.4	hsa-miR-652-3p	24.57	hsa-miR-29c-5p	26.536
hsa-miR-335-3p	30.45	hsa-miR-151a-3p	24.65	hsa-miR-29a-5p	26.536
hsa-miR-532-5p	30.46	hsa-miR-144-3p	24.76	hsa-miR-10a-5p	26.546
hsa-miR-652-3p	30.46	hsa-let-7d-5p	24.76	hsa-miR-186-5p	26.636

hsa-miR-143-3p	30.47	hsa-miR-107	24.76	hsa-miR-34a-3p	26.666
hsa-miR-15b-5p	30.54	hsa-let-7f-5p	24.77	hsa-miR-374b-5p	26.676
hsa-miR-142-3p	30.55	hsa-miR-31-3p	24.8	hsa-miR-100-5p	26.686
hsa-miR-10a-5p	30.55	hsa-miR-191-5p	24.91	hsa-miR-93-3p	26.716
hsa-miR-484	30.66	hsa-miR-10a-5p	24.92	hsa-let-7b-3p	26.716
hsa-miR-146b-5p	30.73	hsa-miR-425-5p	24.93	hsa-miR-652-3p	26.736
hsa-miR-128	30.9	hsa-miR-551a	24.99	hsa-miR-328	26.766
hsa-miR-30d-5p	30.91	hsa-miR-31-5p	25.07	hsa-miR-660-5p	26.816
hsa-miR-139-5p	31.01	hsa-miR-452-5p	25.13	hsa-miR-107	26.816
hsa-miR-191-5p	31.1	hsa-miR-126-5p	25.17	hsa-miR-197-3p	26.876
hsa-miR-31-3p	31.15	hsa-miR-502-3p	25.19	hsa-miR-204-5p	26.886
hsa-miR-199a-5p	31.17	hsa-miR-135b-5p	25.23	hsa-miR-192-5p	26.936
hsa-miR-502-3p	31.17	hsa-miR-181b-5p	25.34	hsa-miR-505-3p	26.946
hsa-miR-145-5p	31.21	hsa-miR-132-3p	25.44	hsa-miR-143-3p	26.956
hsa-let-7b-3p	31.21	hsa-miR-30e-3p	25.49	hsa-miR-214-3p	27.016
hsa-miR-26b-5p	31.22	hsa-miR-30a-3p	25.62	hsa-miR-185-5p	27.036
hsa-miR-328	31.24	hsa-miR-146b-5p	25.64	hsa-miR-199b-5p	27.056
hsa-miR-632	31.3	hsa-miR-26b-5p	25.65	hsa-miR-200a-3p	27.156
hsa-miR-32-5p	31.38	hsa-miR-204-5p	25.66	hsa-miR-324-5p	27.186
hsa-miR-362-3p	31.38	hsa-miR-378a-5p	25.68	hsa-miR-125b-2-3p	27.256
hsa-miR-199b-5p	31.55	hsa-miR-708-5p	25.8	hsa-miR-99b-3p	27.286
hsa-miR-197-3p	31.55	hsa-miR-128	25.81	hsa-miR-421	27.306
hsa-miR-30a-3p	31.68	hsa-miR-200c-3p	25.81	hsa-miR-199a-5p	27.336
hsa-miR-452-5p	31.72	hsa-miR-214-3p	25.88	hsa-miR-214-5p	27.336
hsa-miR-144-3p	31.79	hsa-miR-10b-5p	25.89	hsa-miR-296-5p	27.446
hsa-miR-361-3p	31.81	hsa-miR-374b-5p	25.93	hsa-miR-425-5p	27.496
hsa-miR-551a	31.85	hsa-miR-876-5p	25.98	hsa-let-7f-1-3p	27.516
hsa-miR-142-5p	31.87	hsa-miR-16-2-3p	26.02	hsa-miR-106b-3p	27.596
hsa-miR-193b-3p	31.88	hsa-miR-192-5p	26.04	hsa-miR-2110	27.686
hsa-miR-93-3p	31.98	hsa-miR-224-3p	26.13	hsa-miR-501-3p	27.696
hsa-miR-455-3p	32.02	hsa-miR-15b-3p	26.14	hsa-miR-194-5p	27.716
hsa-miR-214-3p	32.03	hsa-miR-328	26.15	hsa-miR-598	27.776
hsa-miR-324-5p	32.04	hsa-miR-30d-5p	26.17	hsa-miR-769-5p	27.796
hsa-miR-505-3p	32.1	hsa-miR-142-3p	26.17	hsa-miR-18a-5p	27.836
hsa-miR-181b-5p	32.16	hsa-miR-34a-3p	26.19	hsa-miR-339-3p	27.856
hsa-miR-378a-5p	32.18	hsa-miR-598	26.2	hsa-miR-181c-3p	27.966
hsa-miR-30e-3p	32.19	hsa-miR-214-5p	26.23	hsa-miR-18b-5p	27.976
hsa-miR-590-5p	32.25	hsa-miR-181c-5p	26.24	hsa-miR-509-3p	27.976
hsa-miR-135b-5p	32.25	hsa-miR-145-5p	26.27	hsa-miR-215	28.026
hsa-miR-200c-3p	32.3	hsa-miR-514a-3p	26.28	hsa-miR-26b-5p	28.066
hsa-miR-876-5p	32.32	hsa-miR-193a-3p	26.28	hsa-let-7a-3p	28.176
hsa-miR-296-5p	32.43	hsa-let-7b-3p	26.28	hsa-miR-941	28.186
hsa-miR-224-3p	32.44	hsa-miR-363-3p	26.31	hsa-miR-15b-3p	28.196
hsa-miR-29c-5p	32.45	hsa-miR-141-3p	26.33	hsa-miR-181b-5p	28.216
hsa-miR-421	32.5	hsa-miR-32-5p	26.33	hsa-miR-338-3p	28.256
hsa-miR-363-3p	32.5	hsa-miR-455-3p	26.35	hsa-miR-28-5p	28.276
hsa-miR-598	32.52	hsa-miR-484	26.46	hsa-miR-16-5p	28.286
hsa-miR-15b-3p	32.54	hsa-miR-590-5p	26.52	hsa-miR-675-3p	28.286
hsa-miR-374b-5p	32.59	hsa-miR-29c-5p	26.52	hsa-miR-1271-5p	28.286
hsa-miR-214-5p	32.64	hsa-miR-203a	26.56	hsa-miR-148a-3p	28.296
hsa-miR-29a-5p	32.68	hsa-miR-376c-3p	26.61	hsa-miR-326	28.316
hsa-miR-485-3p	32.8	hsa-miR-29a-5p	26.64	hsa-miR-500a-5p	28.336

hsa-miR-16-2-3p	32.81	hsa-miR-505-3p	26.66	hsa-miR-363-3p	28.356
hsa-miR-181c-5p	32.86	hsa-miR-99a-3p	26.67	hsa-miR-139-5p	28.356
hsa-miR-501-3p	32.86	hsa-miR-127-3p	26.71	hsa-miR-141-3p	28.376
hsa-miR-2110	32.87	hsa-miR-509-3p	26.71	hsa-miR-30d-3p	28.426
hsa-miR-141-3p	32.99	hsa-miR-500a-5p	26.81	hsa-miR-221-5p	28.436
hsa-miR-708-5p	33	hsa-miR-513c-5p	26.82	hsa-miR-361-3p	28.446
hsa-miR-34a-3p	33.01	hsa-miR-142-5p	26.84	hsa-miR-376c-3p	28.546
hsa-miR-205-5p	33.04	hsa-miR-194-5p	26.9	hsa-miR-582-5p	28.546
hsa-miR-425-3p	33.04	hsa-miR-29b-2-5p	26.9	hsa-miR-142-5p	28.556
hsa-miR-582-5p	33.05	hsa-miR-28-5p	26.91	hsa-miR-146b-3p	28.556
hsa-miR-374a-5p	33.05	hsa-miR-205-5p	26.94	hsa-miR-26b-3p	28.586
hsa-miR-194-5p	33.06	hsa-miR-98-5p	26.96	hsa-miR-193a-3p	28.596
hsa-miR-509-3p	33.09	hsa-miR-224-5p	26.96	hsa-miR-205-5p	28.626
hsa-miR-192-5p	33.14	hsa-miR-421	26.98	hsa-miR-99a-3p	28.636
hsa-miR-33a-5p	33.21	hsa-miR-361-3p	27.02	hsa-miR-632	28.706
hsa-let-7f-1-3p	33.22	hsa-miR-96-5p	27.03	hsa-miR-30a-5p	28.736
hsa-miR-338-3p	33.23	hsa-miR-125b-2-3p	27.04	hsa-miR-485-3p	28.746
hsa-miR-376c-3p	33.26	hsa-miR-139-5p	27.24	hsa-miR-30e-3p	28.756
hsa-let-7e-3p	33.27	hsa-miR-215	27.26	hsa-miR-125a-3p	28.846
hsa-miR-28-5p	33.29	hsa-miR-18b-5p	27.27	hsa-miR-101-5p	28.846
hsa-miR-513c-5p	33.33	hsa-miR-296-5p	27.29	hsa-miR-342-5p	28.856
hsa-miR-92b-3p	33.41	hsa-miR-374a-5p	27.29	hsa-miR-224-5p	28.866
hsa-miR-224-5p	33.44	hsa-miR-339-5p	27.37	hsa-miR-887	28.876
hsa-miR-424-3p	33.44	hsa-miR-93-3p	27.37	hsa-miR-29b-2-5p	28.916
hsa-miR-98-5p	33.45	hsa-miR-2110	27.5	hsa-let-7f-2-3p	28.916
hsa-miR-106b-3p	33.47	hsa-miR-18a-5p	27.51	hsa-miR-590-5p	29.026
hsa-miR-29b-2-5p	33.48	hsa-miR-425-3p	27.54	hsa-miR-200b-5p	29.026
hsa-miR-203a	33.49	hsa-miR-326	27.57	hsa-miR-664a-3p	29.066
hsa-miR-744-5p	33.52	hsa-miR-106b-3p	27.57	hsa-miR-195-5p	29.106
hsa-miR-99a-3p	33.62	hsa-miR-154-5p	27.61	hsa-miR-133a	29.176
hsa-miR-339-5p	33.73	hsa-miR-501-3p	27.64	hsa-miR-452-5p	29.186
hsa-miR-339-3p	33.82	hsa-let-7f-1-3p	27.65	hsa-miR-144-3p	29.186
hsa-miR-215	33.83	hsa-miR-99b-3p	27.67	hsa-miR-744-5p	29.216
hsa-miR-769-5p	33.87	hsa-miR-339-3p	27.69	hsa-let-7i-3p	29.346
hsa-miR-664a-3p	33.88	hsa-miR-663a	27.9	hsa-miR-505-5p	29.356
hsa-miR-99b-3p	34.03	hsa-miR-376a-3p	27.93	hsa-miR-629-5p	29.406
hsa-miR-887	34.07	hsa-miR-744-5p	27.94	hsa-miR-345-5p	29.426
hsa-miR-454-3p	34.08	hsa-miR-582-5p	27.94	hsa-miR-501-5p	29.456
hsa-miR-500a-5p	34.12	hsa-miR-769-5p	27.96	hsa-miR-361-5p	29.486
hsa-miR-675-3p	34.12	hsa-miR-509-3-5p	28	hsa-miR-146b-5p	29.496
hsa-miR-34c-5p	34.16	hsa-miR-338-3p	28.02	hsa-miR-98-5p	29.536
hsa-miR-642a-5p	34.16	hsa-miR-101-5p	28.06	hsa-miR-425-3p	29.606
hsa-miR-125b-2-3p	34.2	hsa-miR-488-3p	28.18	hsa-miR-409-3p	29.696
hsa-miR-342-5p	34.24	hsa-miR-181c-3p	28.2	hsa-miR-181a-2-3p	29.756
hsa-let-7g-3p	34.25	hsa-let-7i-3p	28.2	hsa-miR-223-5p	29.766
hsa-miR-127-3p	34.31	hsa-miR-887	28.21	hsa-miR-24-1-5p	29.856
hsa-miR-181c-3p	34.34	hsa-let-7g-3p	28.22	hsa-miR-96-5p	29.866
hsa-miR-96-5p	34.36	hsa-miR-345-5p	28.25	hsa-miR-27b-5p	29.876
hsa-miR-34b-5p	34.36	hsa-miR-508-3p	28.28	hsa-miR-128	29.926
hsa-miR-488-3p	34.37	hsa-miR-21-3p	28.33	hsa-miR-605	29.966
hsa-miR-130b-3p	34.39	hsa-miR-382-5p	28.34	hsa-miR-144-5p	30.046

hsa-miR-18a-5p	34.4	hsa-miR-34c-5p	28.36	hsa-miR-663a	30.056
hsa-miR-149-5p	34.41	hsa-let-7a-3p	28.45	hsa-miR-489	30.076
hsa-miR-1271-5p	34.45	hsa-miR-429	28.51	hsa-miR-106b-5p	30.096
hsa-let-7a-3p	34.46	hsa-miR-197-3p	28.53	hsa-miR-125b-1-3p	30.156
hsa-miR-331-3p	34.49	hsa-miR-30d-3p	28.55	hsa-miR-188-3p	30.156
hsa-miR-181d	34.49	hsa-miR-17-5p	28.56	hsa-miR-32-5p	30.186
hsa-miR-17-5p	34.49	hsa-miR-495-3p	28.58	hsa-miR-17-5p	30.186
hsa-miR-125a-3p	34.53	hsa-miR-455-5p	28.59	hsa-miR-502-5p	30.196
hsa-miR-27b-5p	34.6	hsa-miR-144-5p	28.63	hsa-miR-145-3p	30.236
hsa-miR-7-1-3p	34.63	hsa-miR-135a-5p	28.64	hsa-miR-382-5p	30.256
hsa-miR-605	34.69	hsa-miR-629-5p	28.68	hsa-miR-130b-3p	30.316
hsa-miR-221-5p	34.69	hsa-miR-409-3p	28.72	hsa-miR-491-5p	30.326
hsa-miR-629-5p	34.76	hsa-miR-18a-3p	28.73	hsa-miR-135b-5p	30.376
hsa-miR-654-5p	34.78	hsa-miR-671-5p	28.77	hsa-miR-708-5p	30.376
hsa-miR-495-3p	34.8	hsa-miR-181d	28.79	hsa-miR-142-3p	30.396
hsa-miR-346	34.82	hsa-miR-130b-3p	28.83	hsa-miR-20a-3p	30.406
hsa-miR-514a-3p	34.83	hsa-miR-502-5p	28.83	hsa-miR-339-5p	30.526
hsa-miR-101-5p	34.83	hsa-miR-33a-5p	28.84	hsa-miR-127-3p	30.596
hsa-miR-200b-5p	34.85	hsa-miR-513a-3p	28.84	hsa-miR-10b-5p	30.606
hsa-miR-20a-3p	34.87	hsa-miR-377-3p	28.85	hsa-miR-511	30.616
hsa-miR-345-5p	34.89	hsa-miR-1271-5p	28.9	hsa-miR-642a-5p	30.636
hsa-miR-663a	34.9	hsa-miR-454-3p	28.93	hsa-miR-181d	30.656
hsa-miR-30d-3p	34.97	hsa-miR-146b-3p	28.94	hsa-miR-181a-3p	30.686
hsa-miR-651	34.98	hsa-miR-342-5p	28.95	hsa-miR-30d-5p	30.716
hsa-miR-409-3p	34.98	hsa-let-7f-2-3p	28.95	hsa-miR-203a	30.736
hsa-miR-154-5p	35.03	hsa-miR-92b-3p	28.96	hsa-miR-154-5p	30.736
hsa-miR-508-3p	35.04	hsa-miR-26b-3p	28.98	hsa-miR-17-3p	30.736
hsa-miR-429	35.07	hsa-miR-221-5p	29	hsa-miR-132-5p	30.746
hsa-miR-26b-3p	35.07	hsa-miR-642a-5p	29.01	hsa-miR-562	30.776
hsa-miR-24-1-5p	35.07	hsa-miR-424-3p	29.04	hsa-miR-149-5p	30.816
hsa-miR-628-3p	35.08	hsa-miR-34b-5p	29.05	hsa-miR-624-5p	30.816
hsa-miR-144-5p	35.09	hsa-miR-181a-2-3p	29.12	hsa-miR-193b-5p	30.846
hsa-miR-223-5p	35.14	hsa-miR-125a-3p	29.14	hsa-miR-296-3p	30.846
hsa-miR-491-5p	35.18	hsa-miR-7-1-3p	29.18	hsa-miR-24-2-5p	30.916
hsa-miR-18b-5p	35.21	hsa-miR-941	29.19	hsa-miR-23b-5p	30.956
hsa-miR-326	35.27	hsa-miR-200b-5p	29.22	hsa-miR-486-3p	30.966
hsa-miR-21-3p	35.3	hsa-miR-651	29.24	hsa-miR-30e-5p	30.986
hsa-miR-382-5p	35.31	hsa-miR-23b-5p	29.24	hsa-miR-643	31.036
hsa-miR-329	35.32	hsa-miR-188-3p	29.25	hsa-miR-133b	31.066
hsa-miR-501-5p	35.32	hsa-miR-331-3p	29.31	hsa-miR-584-5p	31.086
hsa-miR-511	35.37	hsa-miR-183-5p	29.38	hsa-miR-1908	31.096
hsa-miR-377-3p	35.39	hsa-miR-145-3p	29.5	hsa-miR-130b-5p	31.156
hsa-miR-376a-3p	35.4	hsa-miR-505-5p	29.51	hsa-miR-34b-5p	31.156
hsa-miR-133b	35.42	hsa-miR-511	29.54	hsa-miR-134	31.166
hsa-miR-510	35.47	hsa-miR-503-5p	29.55	hsa-miR-219-5p	31.236
hsa-miR-146b-3p	35.49	hsa-miR-491-5p	29.55	hsa-miR-638	31.256
hsa-miR-18a-3p	35.52	hsa-miR-411-5p	29.66	hsa-miR-504	31.266
hsa-miR-502-5p	35.54	hsa-miR-133a	29.74	hsa-miR-346	31.296
hsa-miR-671-5p	35.6	hsa-miR-133b	29.76	hsa-miR-629-3p	31.386
hsa-miR-582-3p	35.61	hsa-miR-501-5p	29.76	hsa-miR-654-3p	31.396
hsa-miR-206	35.62	hsa-miR-1	29.76	hsa-miR-1247-5p	31.426
hsa-miR-362-5p	35.68	hsa-miR-181a-3p	29.83	hsa-miR-190a	31.486

hsa-miR-190b	35.69	hsa-miR-24-1-5p	29.84	hsa-miR-509-3-5p	31.506
hsa-miR-489	35.73	hsa-miR-584-5p	29.85	hsa-miR-1537	31.516
hsa-let-7a-2-3p	35.73	hsa-miR-506-3p	29.86	hsa-miR-942	31.526
hsa-miR-196b-5p	35.74	hsa-miR-149-5p	29.91	hsa-miR-135b-3p	31.526
hsa-miR-584-5p	35.75	hsa-miR-34c-3p	29.95	hsa-miR-183-5p	31.556
hsa-miR-125b-1-3p	35.75	hsa-miR-138-5p	29.96	hsa-miR-1468	31.576
hsa-miR-217	35.85	hsa-miR-664a-3p	29.98	hsa-miR-429	31.626
hsa-miR-570-3p	35.88	hsa-miR-27b-5p	30.01	hsa-miR-196b-5p	31.636
hsa-miR-17-3p	35.88	hsa-let-7e-3p	30.08	hsa-miR-7-1-3p	31.666
hsa-miR-212-3p	35.91	hsa-miR-628-3p	30.11	hsa-miR-92b-5p	31.676
hsa-let-7f-2-3p	36.05	hsa-miR-155-5p	30.14	hsa-miR-1270	31.736
hsa-miR-135a-5p	36.06	hsa-miR-125b-1-3p	30.14	hsa-miR-582-3p	31.786
hsa-miR-942	36.08	hsa-miR-193b-5p	30.14	hsa-miR-940	31.806
hsa-let-7i-3p	36.08	hsa-miR-675-3p	30.15	hsa-miR-513c-5p	31.816
hsa-miR-155-5p	36.11	hsa-miR-632	30.21	hsa-miR-155-5p	31.826
hsa-miR-23b-5p	36.17	hsa-miR-187-3p	30.22	hsa-miR-513a-3p	31.836
hsa-miR-145-3p	36.17	hsa-miR-188-5p	30.28	hsa-miR-365b-5p	31.846
hsa-miR-454-5p	36.23	hsa-miR-337-5p	30.29	hsa-miR-330-3p	31.956
hsa-miR-503-5p	36.25	hsa-miR-624-5p	30.33	hsa-miR-671-3p	31.956
hsa-miR-187-3p	36.25	hsa-miR-219-5p	30.44	hsa-miR-548c-5p	32.056
hsa-miR-188-5p	36.26	hsa-miR-24-2-5p	30.45	hsa-miR-514a-3p	32.216
hsa-miR-130b-5p	36.26	hsa-miR-346	30.51	hsa-miR-212-3p	32.226
hsa-miR-1468	36.27	hsa-miR-510	30.54	hsa-let-7a-2-3p	32.276
hsa-miR-520h	36.3	hsa-miR-376b-3p	30.55	hsa-miR-10a-3p	32.306
hsa-miR-548c-5p	36.34	hsa-miR-20a-3p	30.56	hsa-miR-503-5p	32.396
hsa-miR-934	36.39	hsa-miR-410	30.63	hsa-miR-33a-5p	32.456
hsa-miR-638	36.41	hsa-miR-362-5p	30.64	hsa-miR-331-5p	32.466
hsa-miR-183-5p	36.44	hsa-miR-330-3p	30.68	hsa-let-7e-3p	32.496
hsa-miR-193a-3p	36.44	hsa-miR-489	30.68	hsa-miR-579	32.506
hsa-miR-188-3p	36.44	hsa-miR-10a-3p	30.68	hsa-miR-25-5p	32.526
hsa-miR-34c-3p	36.49	hsa-miR-940	30.71	hsa-miR-21-3p	32.536
hsa-miR-629-3p	36.49	hsa-miR-485-3p	30.71	hsa-miR-340-5p	32.536
hsa-miR-323a-3p	36.51	hsa-miR-17-3p	30.75	hsa-let-7f-5p	32.556
hsa-miR-493-3p	36.53	hsa-miR-132-5p	30.83	hsa-miR-92b-3p	32.586
hsa-miR-941	36.53	hsa-miR-654-3p	30.99	hsa-miR-628-3p	32.616
hsa-miR-493-3p	36.53	hsa-miR-329	31.03	hsa-miR-337-5p	32.646
hsa-miR-365b-5p	36.54	hsa-miR-134	31.03	hsa-miR-410	32.816
hsa-miR-625-3p	36.56	hsa-miR-323a-3p	31.19	hsa-miR-510	32.816
hsa-miR-671-3p	36.59	hsa-miR-223-5p	31.21	hsa-miR-495-3p	32.826
hsa-miR-548b-3p	36.59	hsa-miR-487b	31.22	hsa-miR-362-5p	32.856
hsa-miR-486-3p	36.61	hsa-miR-942	31.26	hsa-miR-33b-5p	32.856
hsa-miR-643	36.63	hsa-miR-1537	31.27	hsa-miR-34c-5p	32.866
hsa-miR-455-5p	36.65	hsa-miR-212-3p	31.28	hsa-miR-187-3p	32.886
hsa-miR-509-3-5p	36.66	hsa-miR-296-3p	31.33	hsa-let-7g-3p	32.886
hsa-miR-296-3p	36.74	hsa-miR-30c-2-3p	31.36	hsa-miR-190b	32.916
hsa-miR-576-5p	36.76	hsa-miR-627	31.37	hsa-miR-376a-3p	32.976
hsa-miR-615-3p	36.79	hsa-miR-629-3p	31.38	hsa-miR-454-5p	32.996
hsa-miR-331-5p	36.94	hsa-miR-340-5p	31.41	hsa-miR-455-5p	33.016
hsa-miR-627	36.95	hsa-miR-1468	31.7	hsa-miR-708-3p	33.076
hsa-miR-506-3p	36.97	hsa-miR-1247-5p	31.73	hsa-miR-377-3p	33.116
hsa-miR-1247-5p	37.03	hsa-miR-190a	31.77	hsa-miR-34c-3p	33.176

hsa-miR-940	37.05	hsa-miR-20b-5p	31.81	hsa-miR-616-5p	33.176
hsa-miR-20b-5p	37.06	hsa-miR-33b-5p	31.82	hsa-miR-411-5p	33.216
hsa-miR-1537	37.06	hsa-miR-548b-3p	31.88	hsa-miR-508-5p	33.326
hsa-miR-1908	37.08	hsa-miR-365b-5p	31.89	hsa-miR-20b-5p	33.336
hsa-miR-134	37.11	hsa-miR-26a-2-3p	31.9	hsa-miR-589-3p	33.496
hsa-miR-190a	37.15	hsa-miR-643	31.9	hsa-miR-493-3p	33.556
hsa-miR-616-5p	37.15	hsa-miR-1908	31.9	hsa-miR-493-3p	33.556
hsa-miR-579	37.15	hsa-miR-25-5p	31.93	hsa-miR-1207-5p	33.566
hsa-miR-330-3p	37.3	hsa-miR-671-3p	31.94	hsa-miR-323a-3p	33.666
hsa-miR-133a	37.3	hsa-miR-135b-3p	31.98	hsa-miR-625-3p	33.746
hsa-miR-1914-5p	37.35	hsa-miR-579	32.02	hsa-miR-135a-5p	33.746
hsa-miR-182-3p	37.39	hsa-miR-570-3p	32.07	hsa-miR-627	33.786
hsa-miR-92b-5p	37.39	hsa-miR-454-5p	32.08	hsa-miR-18a-3p	33.786
hsa-miR-337-5p	37.42	hsa-miR-1270	32.08	hsa-miR-338-5p	33.806
hsa-miR-411-5p	37.48	hsa-miR-582-3p	32.1	hsa-miR-188-5p	33.986
hsa-miR-450a-5p	37.51	hsa-miR-130b-5p	32.14	hsa-miR-576-5p	33.986
hsa-miR-545-3p	37.58	hsa-miR-655	32.15	hsa-miR-1538	34.026
hsa-miR-550a-5p	37.69	hsa-miR-616-5p	32.15	hsa-miR-628-5p	34.076
hsa-miR-181a-3p	37.7	hsa-miR-548c-5p	32.16	hsa-miR-506-3p	34.216
hsa-miR-340-5p	37.74	hsa-miR-23a-5p	32.2	hsa-miR-124-3p	34.236
hsa-miR-25-5p	37.75	hsa-miR-708-3p	32.22	hsa-miR-138-5p	34.256
hsa-miR-10a-3p	37.76	hsa-miR-216a-5p	32.28	hsa-miR-487b	34.276
hsa-miR-1255b-5p	37.86	hsa-miR-576-5p	32.3	hsa-miR-654-5p	34.396
hsa-miR-193b-5p	37.86	hsa-miR-562	32.31	hsa-miR-548b-3p	34.456
hsa-miR-1207-5p	37.92	hsa-miR-504	32.32	hsa-miR-508-3p	34.466
hsa-miR-135b-3p	37.93	hsa-let-7a-2-3p	32.54	hsa-miR-23a-5p	34.476
hsa-miR-562	37.95	hsa-miR-628-5p	32.59	hsa-miR-1	34.556
hsa-miR-138-5p	37.98	hsa-miR-545-3p	32.62	hsa-miR-216a-5p	34.566
hsa-miR-219-5p	37.99	hsa-miR-486-3p	32.65	hsa-miR-1914-5p	34.626
hsa-miR-26a-2-3p	37.99	hsa-miR-654-5p	32.68	hsa-miR-550a-5p	34.636
hsa-miR-181a-2-3p	38.15	hsa-miR-217	32.73	hsa-miR-217	34.676
hsa-miR-504	38.18	hsa-miR-182-3p	32.8	hsa-miR-182-3p	34.696
hsa-miR-1538	38.18	hsa-miR-331-5p	32.82	hsa-miR-362-3p	34.796
hsa-miR-665	38.22	hsa-miR-493-3p	32.83	hsa-miR-615-3p	34.916
hsa-miR-624-5p	38.23	hsa-miR-493-3p	32.83	hsa-miR-376b-3p	34.936
hsa-miR-487b	38.25	hsa-miR-625-3p	32.87	hsa-let-7a-5p	34.946
hsa-miR-628-5p	38.25	hsa-miR-92b-5p	32.94	hsa-miR-876-5p	35.016
hsa-miR-654-3p	38.26	hsa-miR-638	33.01	hsa-miR-520h	35.156
hsa-miR-708-3p	38.28	hsa-miR-508-5p	33.05	hsa-miR-26a-2-3p	35.386
hsa-miR-376b-3p	38.31	hsa-miR-206	33.3	hsa-miR-545-3p	35.416
hsa-miR-23a-5p	38.42	hsa-miR-605	33.32	hsa-miR-330-5p	35.456
hsa-miR-24-2-5p	38.44	hsa-miR-1207-5p	33.34	hsa-miR-155-3p	35.486
hsa-miR-1249	38.51	hsa-miR-190b	33.71	hsa-miR-655	35.556
hsa-miR-30c-2-3p	38.58	hsa-miR-589-3p	33.89	hsa-miR-934	35.596
hsa-miR-1	38.6	hsa-miR-550a-5p	33.95	hsa-miR-331-3p	35.686
hsa-miR-513a-3p	38.64	hsa-miR-124-3p	34.05	hsa-miR-1249	35.896
hsa-miR-589-3p	38.65	hsa-miR-330-5p	34.05	hsa-miR-424-3p	35.966
hsa-miR-410	38.75	hsa-miR-615-3p	34.17	hsa-miR-206	36.026
hsa-miR-508-5p	38.77	hsa-miR-196b-5p	34.2	hsa-miR-651	36.126
hsa-miR-330-5p	38.84	hsa-miR-450a-5p	34.59	hsa-miR-1471	36.186

hsa-miR-655	38.94	hsa-miR-1255b-5p	34.64	hsa-miR-1255b-5p	36.426
hsa-miR-1471	39.03	hsa-miR-665	34.67	hsa-miR-570-3p	36.446
hsa-miR-216a-5p	39.33	hsa-miR-1538	35.74	hsa-miR-671-5p	36.586
hsa-miR-124-3p	39.47	hsa-miR-338-5p	35.97	hsa-miR-335-3p	37.236
hsa-miR-155-3p	39.54	hsa-miR-520h	36.52	hsa-miR-665	37.416
hsa-miR-338-5p	39.57	hsa-miR-1914-5p	36.67	hsa-miR-329	37.686
hsa-miR-33b-5p	39.59	hsa-miR-1249	36.92	hsa-miR-30c-2-3p	37.696
hsa-miR-1270	39.85	hsa-miR-934	36.98	hsa-miR-450a-5p	37.806
hsa-miR-505-5p	39.89	hsa-miR-155-3p	37.13	hsa-miR-488-3p	37.866
hsa-miR-132-5p	39.97	hsa-miR-1471	39.24	hsa-miR-454-3p	37.956

Table S3. Particulars of the 16 cardiovascular microRNAs that were selected for further investigation

miRNAs	Previous IDs	Assay ID code	Position in PF sample			Previously reported expressed in these cardiovascular tissues/cells or progenitor cells	Reported cardiovascular functions	Reference
			1	2	3			
let-7b-5p	let-7b	000378	8	12	15	Human mesenchymal stem cells (MSCs)	Let-7b-overexpressing MSCs promote heart regeneration in rats	1
miR-15a-5p	miR-15a	000389	16	13	9	Rat myocardium and cardiomyocytes<br>Human vascular smooth muscle, endothelial cells and bone marrow–derived proangiogenic cells	Induces cardiomyocyte apoptosis<br>Anti-angiogenic<br>Anti-proliferative for vascular cells	2-4
miR-16-5p	miR-16	000391	1	2	140	Rat myocardium and cardiomyocytes<br>Human bone marrow–derived proangiogenic cells	Reduces cardiomyocyte hypertrophy<br>Anti-angiogenic	3,5
miR-19b-3p	miR-19b	000396	10	11	5	Rat cardiomyocytes	Induces cardiomyocyte hypertrophy<br>Anti-apoptotic<br>Anti-angiogenic	6
miR-21-5p	miR-21	000397	2	3	2	Rodent cardiomyocytes and cardiac fibroblast,<br>Human Endothelial cells	Anti-apoptotic<br>Anti-angiogenic<br>Proangiogenic in the chicken chorioallantoic membrane assay and in cancer	7-10
miR-22-3p	miR-22	000398	15	9	8	Rat myocardium and cardiomyocytes	Induces cardiomyocyte hypertrophy	11
miR-23a-3p	miR-23a	000399	5	10	10	Rodent heart and cardiomyocytes	Induces cardiomyocyte hypertrophy	12,13

miR-24-3p	miR-24	000402	6	5	4	Mouse heart, cardiomyocytes, cardiac fibroblasts and myocardial endothelial cells<br>Human endothelial cells<br>Mouse and human aorta	Inhibits cardiomyocytes apoptosis<br>Anti-fibrotic<br>Anti-angiogenic<br>Limits aneurysm evolution	12,14-17
miR-27a-3p	miR-27a	000408	40	28	21	Rat cardiomyocytes Human endothelial cell	Reduces cardiomyocyte hypertrophy<br>Pro-angiogenic	6,11,18
miR-27b-3p	miR-27b	000409	7	8	16	Rodent heart and cardiomyocytes<br>Human endothelial cells	Induces cardiomyocyte hypertrophy<br>Pro-angiogenic	19
miR-29a-3p	miR-29a	002112	9	6	6	Rat cardiac myocytes (immortalized H9c2 cell line)<br>Human endothelial cells<br>Murine endothelial cells	Inhibits cardiomyocyte proliferation<br>Pro-angiogenic	20,21
miR-29b-3p	miR-29b	000413	51	16	19	Rodent heart and cardiac fibroblasts	Antifibrotic	22,23
miR-29c-3p	miR-29c	000587	11	7	11	Human endothelial cells	Anti-angiogenic	24
miR-126-3p	miR-126	002228	59	71	59	Mouse and human endothelial cells	Pro-angiogenic	25,26
miR-208a	miR-208	000511	N.D	N.D.	N.D.	Rat, murine and human myocardium	Induces cardiomyocyte hypertrophy<br>Pro-fibrotic	27-29
miR-451a	miR-451	001141	3	1	1	Rat cardiac myocytes (immortalized H9c2 cell line)<br>Mouse heart	Anti-apoptotic	30,31

N.D.: Not detected

Additional References for Table S3

1. *Ham, O, Lee, S-Y, Lee, CY, Park, J-H, Lee, J, Seo, H-H, et al.* (2015). let-7b suppresses apoptosis and autophagy of human mesenchymal stem cells transplanted into ischemia/reperfusion injured heart 7by targeting caspase-3. *Stem Cell Res Ther* 6: 147.
2. Brittan, M, Hunter, A, Boulberdaa, M, Fujisawa, T, Skinner, EM, Shah, AS, *et al.* (2015). Impaired vascular function and repair in patients with premature coronary artery disease. *Eur J Prev Cardiol* 22: 1557–1566.
3. Liu, L, Zhang, G, Liang, Z, Liu, X, Li, T, Fan, J, *et al.* (2014). MicroRNA-15b enhances hypoxia/reoxygenation-induced apoptosis of cardiomyocytes via a mitochondrial apoptotic pathway. *Apoptosis* 19: 19–29.
4. Spinetti, G, Fortunato, O, Caporali, A, Shantikumar, S, Marchetti, M, Meloni, M, *et al.* (2013). MicroRNA-15a and microRNA-16 impair human circulating proangiogenic cell functions and are increased in the proangiogenic cells and serum of patients with critical limb ischemia. *Circ. Res.* 112: 335–346.
5. Zheng, X, Li, A, Zhao, L, Zhou, T, Shen, Q, Cui, Q, *et al.* (2013). Key role of microRNA-15a in the KLF4 suppressions of proliferation and angiogenesis in endothelial and vascular smooth muscle cells. *Biochem. Biophys. Res. Commun.* 437: 625–631.
6. Huang, S, Zou, X, Zhu, J-N, Fu, Y-H, Lin, Q-X, Liang, Y-Y, *et al.* (2015). Attenuation of microRNA-16 derepresses the cyclins D1, D2 and E1 to provoke cardiomyocyte hypertrophy. *J. Cell. Mol. Med.* 19: 608–619.
7. Song, DW, Ryu, JY, Kim, JO, Kwon, EJ and Kim, DH (2014). The miR-19a/b family positively regulates cardiomyocyte hypertrophy by targeting atrogin-1 and MuRF-1. *Biochem. J.* 457: 151–162.
8. Thum, T, Gross, C, Fiedler, J, Fischer, T, Kissler, S, Bussen, M, *et al.* (2008). MicroRNA-21 contributes to myocardial disease by stimulating MAP kinase signalling in fibroblasts. *Nature* 456: 980–984.
9. Cheng, Y, Zhu, P, Yang, J, Liu, X, Dong, S, Wang, X, *et al.* (2010). Ischaemic preconditioning-regulated miR-21 protects heart against ischaemia/reperfusion injury via anti-apoptosis through its target PDCD4. *Cardiovasc. Res.* 87: 431–439.
10. Liu, L-Z, Li, C, Chen, Q, Jing, Y, Carpenter, R, Jiang, Y, *et al.* (2011). MiR-21 induced angiogenesis through AKT and ERK activation and HIF-1α expression. *PLoS ONE* 6: e19139.
11. Sabatel, C, Malvaux, L, Bovy, N, Deroanne, C, Lambert, V, Gonzalez, M-LA, *et al.* (2011). MicroRNA-21 exhibits antiangiogenic function by targeting RhoB expression in endothelial cells. *PLoS ONE* 6: e16979.
12. Jentzsch, C, Leierseder, S, Loyer, X, Flohrschütz, I, Sassi, Y, Hartmann, D, *et al.* (2012). A phenotypic screen to identify hypertrophy-modulating microRNAs in primary cardiomyocytes. *J. Mol. Cell. Cardiol.* 52: 13–20.
13. Xu, X-D, Song, X-W, Li, Q, Wang, G-K, Jing, Q and Qin, Y-W (2012). Attenuation of microRNA-22 derepressed PTEN to effectively protect rat cardiomyocytes from hypertrophy. *J. Cell. Physiol.* 227: 1391–1398.
14. Qian, L, Van Laake, LW, Huang, Y, Liu, S, Wendland, MF and Srivastava, D (2011). miR-24 inhibits apoptosis and represses Bim in mouse cardiomyocytes. *J. Exp. Med.* 208: 549–560.
15. Lin, Z, Murtaza, I, Wang, K, Jiao, J, Gao, J and Li, P-F (2009). miR-23a functions downstream of NFATc3 to regulate cardiac hypertrophy. *Proc. Natl. Acad. Sci. U.S.A.* 106: 12103–12108.
16. Wang, K, Lin, Z-Q, Long, B, Li, J-H, Zhou, J and Li, P-F (2012). Cardiac hypertrophy is positively regulated by MicroRNA miR-23a. *J. Biol. Chem.* 287: 589–599.
17. Wang, J, Huang, W, Xu, R, Nie, Y, Cao, X, Meng, J, *et al.* (2012). MicroRNA-24 regulates cardiac fibrosis after myocardial infarction. *J. Cell. Mol. Med.* 16: 2150–2160.
18. Fiedler, J, Jazbutyte, V, Kirchmaier, BC, Gupta, SK, Lorenzen, J, Hartmann, D, *et al.* (2011). MicroRNA-24 regulates vascularity after myocardial infarction. *Circulation* 124: 720–730.
19. Meloni, M, Marchetti, M, Garner, K, Littlejohns, B, Sala-Newby, G, Xenophontos, N, *et al.* (2013). Local inhibition of microRNA-24 improves reparative angiogenesis and left ventricle remodeling and function in mice with myocardial infarction. *Mol. Ther.* 21: 1390–1402.
20. Maegdefessel, L, Spin, JM, Raaz, U, Eken, SM, Toh, R, Azuma, J, *et al.* (2014). miR-24 limits aortic vascular inflammation and murine abdominal aneurysm development. *Nat Commun* 5: 5214.
21. Urbich, C, Kaluza, D, Frömel, T, Knau, A, Bennewitz, K, Boon, RA, *et al.* (2012). MicroRNA-27a/b controls endothelial cell repulsion and angiogenesis by targeting semaphorin 6A. *Blood* 119: 1607–1616.
22. Cao, X, Wang, J, Wang, Z, Du, J, Yuan, X, Huang, W, *et al.* (2013). MicroRNA profiling

during rat ventricular maturation: A role for miR-29a in regulating cardiomyocyte cell cycle re-entry. *FEBS Lett.* 587: 1548–1555.
23. Yang, Z, Wu, L, Zhu, X, Xu, J, Jin, R, Li, G, *et al.* (2013). MiR-29a modulates the angiogenic properties of human endothelial cells. *Biochem. Biophys. Res. Commun.* 434: 143–149.
24. Abonnenc, M, Nabeebaccus, AA, Mayr, U, Barallobre-Barreiro, J, Dong, X, Cuello, F, *et al.* (2013). Extracellular matrix secretion by cardiac fibroblasts: role of microRNA-29b and microRNA-30c. *Circ. Res.* 113: 1138–1147.
25. Zhu, J-N, Chen, R, Fu, Y-H, Lin, Q-X, Huang, S, Guo, L-L, *et al.* (2013). Smad3 inactivation and MiR-29b upregulation mediate the effect of carvedilol on attenuating the acute myocardium infarction-induced myocardial fibrosis in rat. *PLoS ONE* 8: e75557.
26. Hu, Y, Deng, F, Song, J, Lin, J, Li, X, Tang, Y, *et al.* (2015). Evaluation of miR-29c inhibits endotheliocyte migration and angiogenesis of human endothelial cells by suppressing the insulin like growth factor 1. *Am J Transl Res* 7: 489–501.
27. Wang, S, Aurora, AB, Johnson, BA, Qi, X, McAnally, J, Hill, JA, *et al.* (2008). The endothelial-specific microRNA miR-126 governs vascular integrity and angiogenesis. *Dev. Cell* 15: 261–271.
28. Fish, JE, Santoro, MM, Morton, SU, Yu, S, Yeh, R-F, Wythe, JD, *et al.* (2008). miR-126 regulates angiogenic signaling and vascular integrity. *Dev. Cell* 15: 272–284.
29. van Solingen, C, Seghers, L, Bijkerk, R, Duijs, JMGJ, Roeten, MK, van Oeveren-Rietdijk, AM, *et al.* (2009). Antagomir-mediated silencing of endothelial cell specific microRNA-126 impairs ischemia-induced angiogenesis. *J. Cell. Mol. Med.* 13: 1577–1585.
30. Montgomery, RL, Hullinger, TG, Semus, HM, Dickinson, BA, Seto, AG, Lynch, JM, *et al.* (2011). Therapeutic inhibition of miR-208a improves cardiac function and survival during heart failure. *Circulation* 124: 1537–1547.
31. van Rooij, E, Sutherland, LB, Qi, X, Richardson, JA, Hill, J and Olson, EN (2007). Control of stress-dependent cardiac growth and gene expression by a microRNA. *Science* 316: 575–579.
32. Callis, TE, Pandya, K, Seok, HY, Tang, R-H, Tatsuguchi, M, Huang, Z-P, *et al.* (2009). MicroRNA-208a is a regulator of cardiac hypertrophy and conduction in mice. *J. Clin. Invest.* 119: 2772–2786.
33. Zhang, Y, Wang, Y, Wang, XK and Zeng, C (2011). Insulin promotes vascular smooth muscle cell proliferation via microrna-208 mediated down-regulation of p21. *Journal of Hypertension* 29: e427.
34. Zhang, X, Wang, X, Zhu, H, Zhu, C, Wang, Y, Pu, WT, *et al.* (2010). Synergistic effects of the GATA-4-mediated miR-144/451 cluster in protection against simulated ischemia/reperfusion-induced cardiomyocyte death. *J. Mol. Cell. Cardiol.* 49: 841–850.
35. Wang, X, Zhu, H, Zhang, X, Liu, Y, Chen, J, Medvedovic, M, *et al.* (2012). Loss of the miR-144/451 cluster impairs ischaemic preconditioning-mediated cardioprotection by targeting Rac-1. *Cardiovasc. Res.* 94: 379–390.

Supplemental raw data

CT from the whole PF qPCR analyses on the selective 16 putative cardiovascular miRNAs used to validate the Exiqon array
(The CT value in the Exiqon array are shown for comparison)

	miR-19b	
	**qPCR CT**	**Array Ct**
	28.825	26.04
	25.675	20.04
	26.46	20.466
	27.25	/
	31.255	/
**Average**	27.893	22.182
**St Dev**	2.211228731	3.347908601
**CV**	0.0792754	0.150929069

	miR-27b	
	**qPCR CT**	**Array Ct**
	28.885	25.41
	29.41	19.55
	30.19	21.606
	35	/
	31.505	/
**Average**	30.998	22.18866667
**St Dev**	2.445016871	2.973133925
**CV**	0.078876601	0.133993357

	miR-29c	
	**qPCR CT**	**Array Ct**
	27.405	26.06
	25.46	19.52
	27.015	21.186
	25.525	/
	30.75	/
**Average**	27.231	22.25533333
**St Dev**	2.150998024	3.398603439
**CV**	0.078990783	0.152709617

	Let-7b	
	qPCR CT	Array Ct
	25.635	25.54
	23.255	20.06
	23.1	21.476
	25.2	/
	25.01	/
**Average**	24.44	22.35866667
**St Dev**	1.17583481	2.844630966
**CV**	0.048111081	0.127227218

	miR-22	
	qPCR CT	Array Ct
	31.42	26.39
	26.18	19.62
	27.24	20.926
	30.135	/
	31.99	/
**Average**	29.393	22.312
**St Dev**	2.567220287	3.591513887
**CV**	0.087341213	0.160967815

	miR-15a	
	qPCR CT	Array Ct
	35	26.44
	34.275	20.31
	34.345	21.036
	35	/
	35	/
**Average**	34.724	22.59533333
**St Dev**	0.378738036	3.349308187
**CV**	0.010907097	0.148230085

	miR-208	
	qPCR CT	Array Ct
	n.d.	*n.d.*
	22.49	*n.d.*
	23.395	*n.d.*
	23.415	/
	27.35	/
**Average**	24.1625	/
**St Dev**	2.168349987	/
**CV**	0.0897403	/

	miR-16	
	qPCR CT	Array Ct
	26.73	23.12
	22.49	17
	23.395	28.286
	23.415	/
	27.35	/
**Average**	24.676	22.802
**St Dev**	2.201071898	5.649716099
**CV**	0.089198894	0.247772831

	miR-21-5p	
	qPCR CT	Array Ct
	23.835	23.19
	21.823	17.08
	29.715	20.256
	27.288	
	28.245	
	/	
	19.545	
	21.168	
	23.416	
**Average**	24.379	20.17533333
**St Dev**	3.651116522	3.055798641
**CV**	0.149762928	0.151462114

	miR-23a-3p	
	qPCR CT	Array Ct
	28.186	24.82
	28.309	19.66
	30.873	21.176
	30.729	
	30.845	
	26.733	
	27.178	
	28.403	
	26.803	
**Average**	28.673	21.88533333
**St Dev**	1.722113082	2.652124683
**CV**	0.060059838	0.121182741

	miR-24-3p	
	qPCR CT	Array Ct
	24.431<br>22.045<br>27.951<br>27.256<br>27.984<br>21.858<br>21.899<br>19.854<br>22.560	25.08<br>19.12<br>20.386
**Average**	23.982	21.52866667
**St Dev**	3.048448074	3.140010403
**CV**	0.12711311	0.145852525

	miR-27a-3p	
	qPCR CT	Array Ct
	28.632<br>27.173<br>32.802<br>30.25076365<br>26.28535959<br>33.70680682<br>22.030<br>24.566	28.03<br>21.79<br>22.396
**Average**	28.181	24.072
**St Dev**	3.99786915	3.441094593
**CV**	0.141865501	0.142950091

	miR-29a-3p	
	qPCR CT	Array Ct
	22.74752856<br>18.75318504<br>18.64544536<br>18.44289977<br>24.56168632<br>25.1331412<br>20.28724242<br>18.634<br>21.643	25.86<br>19.28<br>20.656
**Average**	20.983	21.932
**St Dev**	2.656806664	3.470624151
**CV**	0.126616253	0.158244763

	miR-29b-3p	
	qPCR CT	Array Ct
	32.32929513	28.45
	30.68970959	20.72
	33.63064721	22.286
	35.4524306	
	34.59613255	
	30.55840402	
	36.1804272	
	27.59420613	
	29.40250852	
**Average**	32.270	23.81866667
**St Dev**	2.91970691	4.086566448
**CV**	0.090476266	0.171569908

	miR-126-3p	
	qPCR CT	Array Ct
	28.41164267	28.83
	26.28378965	23.86
	33.209562	25.416
	36.01387719	
	27.83337422	
	32.64411207	
	29.27828862	
	33.40568267	
	/	
**Average**	30.885	26.03533333
**St Dev**	3.387790275	2.542224485
**CV**	0.109690327	0.097645168

	miR-451	
	qPCR CT	Array Ct
	23.105	23.4
	21.33	16.49
	21.795	16.606
	21.8	/
	25.595	/
**Average**	22.725	18.832
**St Dev**	1.735500648	3.956429198
**CV**	0.076369665	0.21009076

Raw data of the paragraph: *Human PF is enriched with miRNAs of potential cardiovascular origin.*

Raw data are provided for the 3 miRs that are not shown in Figure 1.

	miR-19b-3p	
**Samples**	**Relative Expression PF**	**Relative Expression PLASMA**
1	1.97E-03	2.60E-02
2	5.08E-03	1.55E-02
3	8.64E-03	6.62E-03
4	1.81E-03	1.05E-02
5	6.47E-04	9.38E-05

	miR-15a-3p	
**Samples**	**Relative Expression PF**	**Relative Expression PLASMA**
1	7.540E-05	4.7139E-01
2	6.915E-05	6.1854E-02
3	1.844E-04	4.2484E-01
4	7.309E-05	2.2144E-01
5	2.709E-04	8.8388E-02

	miR-126-3p	
**Samples**	**Relative Expression PF**	**Relative Expression PLASMA**
1	5.0000E-01	7.0711E-01
2	2.5000E-01	8.4090E-01
3	1.2500E-01	9.1700E-01
4	6.2500E-02	9.5760E-01
5	3.1250E-02	9.7857E-01
6	1.5625E-02	9.8923E-01
7	7.8125E-03	9.9460E-01
8	3.9063E-03	9.9730E-01

Raw data of the paragraph: Human PF contains exosomes carrying cardiovascular miRNAs

Raw data are provided for the 3 miRs that are not shown in Figure 3.

	miR-27a-3p	
**Samples**	**Relative Expression PF**	**Relative Expression PLASMA**
1	2.07E-05	6.01E-05
2	9.65E-06	3.15E-05
3	4.54E-05	2.25E-05
4	3.70E-05	6.43E-06
5	1.35E-05	4.66E-05

	miR-15a-5p	
**Samples**	**Relative Expression PF**	**Relative Expression PLASMA**
1	1.08271E-05	0.025207555
2	6.96267E-05	4.30666E-06
3	5.59695E-05	0.036397925
4	1.22658E-05	0.009888723
5	4.18889E-06	0.002733566

	miR-126-3p	
**Samples**	**Relative Expression PF**	**Relative Expression PLASMA**
1	2.3651E-05	1.9199E-04
2	3.0819E-06	9.1028E-05
3	5.2289E-06	3.9714E-05
4	3.5000E-06	3.6629E-05
5	1.5089E-06	2.6752E-05

Relative abundance of the 16 cardiovascular miRNAs and of miR-122 in the aorta and myocardium tissues in and in PF exosomes

Let-7b		
**Relative Expression (versus U6) Aorta**	**Relative Expression (versus U6) Myocardium**	**Relative Expression (versus cel-miR-39) PF exosome**
2.558	1.729	1.17×10^{-3}
2.742	3.797	5.64×10^{-3}
12.906	4.272	3.0×10^{-3}
2.092	4.141	1.54×10^{-3}
3.352		6.69×10^{-4}

miR-15a-5p		
**Relative Expression Aorta**	**Relative Expression Myocardium**	**Relative Expression PF exosome**
1.000	0.293	1.08×10^{-5}
0.981	0.574	6.96×10^{-5}
0.946	0.642	5.60×10^{-5}
0.985	0.020	1.23×10^{-5}
0.961		4.19×10^{-6}

miR-16-5p		
**Relative Expression Aorta**	**Relative Expression Myocardium**	**Relative Expression PF exosome**
0.000	0.171	1.47×10^{-2}
0.023	0.015	7.97×10^{-2}
0.001	0.139	4.70×10^{-2}
0.000	0.020	1.59×10^{-2}
0.001	0.171	4.63×10^{-3}

miR-19b-3p		
**Relative Expression (versus U6) Aorta**	**Relative Expression (versus U6) Myocardium**	**Relative Expression (versus cel-miR-39) PF exosome**
0.956	1.102	4.18×10^{-4}
1.145	1.177	2.77×10^{-4}
3.694	0.272	9.14×10^{-4}
0.914	0.540	4.30×10^{-4}
2.354		1.52×10^{-4}

miR-21-5p		
**Relative Expression Aorta**	**Relative Expression Myocardium**	**Relative Expression PF exosome**
0.381	1.438	7.619×10^{-4}
0.431	1.025	5.265×10^{-4}
0.360	8.889	4.583×10^{-4}
0.952		4.970×10^{-4}
3.967		9.665×10^{-4}
1.030		
1.954		

miR-22-3p		
**Relative Expression Aorta**	**Relative Expression Myocardium**	**Relative Expression PF exosome**
0.480	0.369	2.75×10^{-4}
0.278	0.118	2.33×10^{-3}
0.865	8.664	1.85×10^{-4}
0.176	0.139	1.50×10^{-4}
1.113		2.64×10^{-4}

miR-23a-3p		
**Relative Expression (versus U6) Aorta**	**Relative Expression (versus U6) Myocardium**	**Relative Expression (versus cel-miR-39) PF exosome**
0.010	0.241	5.118×10^{-5}
0.031	0.147	1.960×10^{-5}
0.031	0.665	1.359×10^{-5}
0.018		9.630×10^{-6}
0.249		4.455×10^{-5}
0.125		
0.181		

miR-24-3p		
**Relative Expression (versus U6) Aorta**	**Relative Expression (versus U6) Myocardium**	**Relative Expression (versus cel-miR-39) PF exosome**
0.563	3.242	4.952×10^{-4}
1.056	3.358	1.085×10^{-3}
0.664	13.878	1.367×10^{-4}
0.066		7.375×10^{-4}
3.698		6.241×10^{-4}
2.438		
9.161		

miR-27a-3p		
**Relative Expression (versus U6) Aorta**	**Relative Expression (versus U6) Myocardium**	**Relative Expression (versus cel-miR-39) PF exosome**
0.314	0.738	2.07×10^{-5}
0.747	0.775	9.65×10^{-6}
0.445	4.286	4.54×10^{-5}
0.414		3.70×10^{-5}
1.580		1.35×10^{-5}
0.913		
1.676		

miR-27b-3p		
**Relative Expression (versus U6) Aorta**	**Relative Expression (versus U6) Myocardium**	**Relative Expression (versus cel-miR-39) PF exosome**
0.318	2.274	4.83×10^{-4}
1.173	2.346	1.55×10^{-4}
4.908	3.811	1.03×10^{-5}
1.699	0.425	2.94×10^{-5}
4.579		1.20×10^{-5}
0.318		
1.173		

miR-29a-3p		
**Relative Expression (versus U6) Aorta**	**Relative Expression (versus U6) Myocardium**	**Relative Expression (versus cel-miR-39) PF exosome**
0.281	1.152	2.24×10^{-4}
0.484	1.919	9.40×10^{-4}
0.375	9.338	3.32×10^{-4}
0.221		1.05×10^{-4}
3.208		9.39×10^{-4}
3.531		
3.557		

miR-29b-3p		
**Relative Expression (versus U6) Aorta**	**Relative Expression (versus U6) Myocardium**	**Relative Expression (versus cel-miR-39) PF exosome**
0.018	0.033	1.34×10^{-6}
0.018	0.044	6.92×10^{-6}
0.025	0.269	1.41×10^{-6}
0.019		2.09×10^{-6}
0.139		2.45×10^{-6}
0.163		
0.259		

miR-29c-3p		
**Relative Expression (versus U6) Aorta**	**Relative Expression (versus U6) Myocardium**	**Relative Expression (versus cel-miR-39) PF exosome**
1.693	3.837	7.53×10^{-4}
2.990	2.540	1.05×10^{-3}
8.545	1.193	6.69×10^{-4}
1.053	1.244	7.96×10^{-4}
4.243		5.11×10^{-4}

miR-126-3p		
**Relative Expression (versus U6) Aorta**	**Relative Expression (versus U6) Myocardium**	**Relative Expression (versus cel-miR-39) PF exosome**
0.061	1.770	2.37×10^{-5}
0.059	0.243	3.08×10^{-6}
0.036	1.586	5.23×10^{-6}
0.104		3.50×10^{-6}
2.911		1.51×10^{-6}
2.136		
3.383		

miR-208a-3p		
**Relative Expression (versus U6) Aorta**	**Relative Expression (versus U6) Myocardium**	**Relative Expression (versus cel-miR-39) PF exosome**
0.991	1.915	1.47×10^{-2}
1.055	2.959	7.97×10^{-2}
1.087	0.740	4.70×10^{-2}
1.003	3.090	1.59×10^{-2}
1.017		4.63×10^{-3}

miR-451a		
**Relative Expression (versus U6) Aorta**	**Relative Expression (versus U6) Myocardium**	**Relative Expression (versus cel-miR-39) PF exosome**
0.804	0.027	2.33×10^{-2}
6.453	3.986	2.22×10^{-2}
20.112	10.928	2.34×10^{-2}
17.569	6.277	1.79×10^{-2}
27.284		5.56×10^{-3}

miR-122-5p		
**Relative Expression (versus U6) Aorta**	**Relative Expression (versus U6) Myocardium**	**Relative Expression (versus cel-miR-39) PF exosome**
0.000	0.000	0.001
0.000	0.000	0.001
0.000	0.000	0.001
0.000	0.000	0.001
0.000		0.001
0.000		
0.000		

Raw data of the paragraph: PF exosomes contain the RISC components Dicer and Ago-2

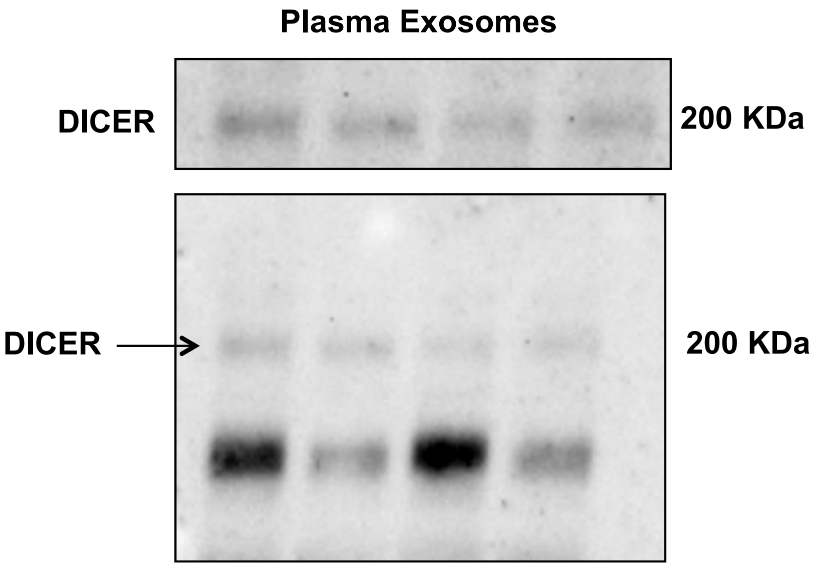

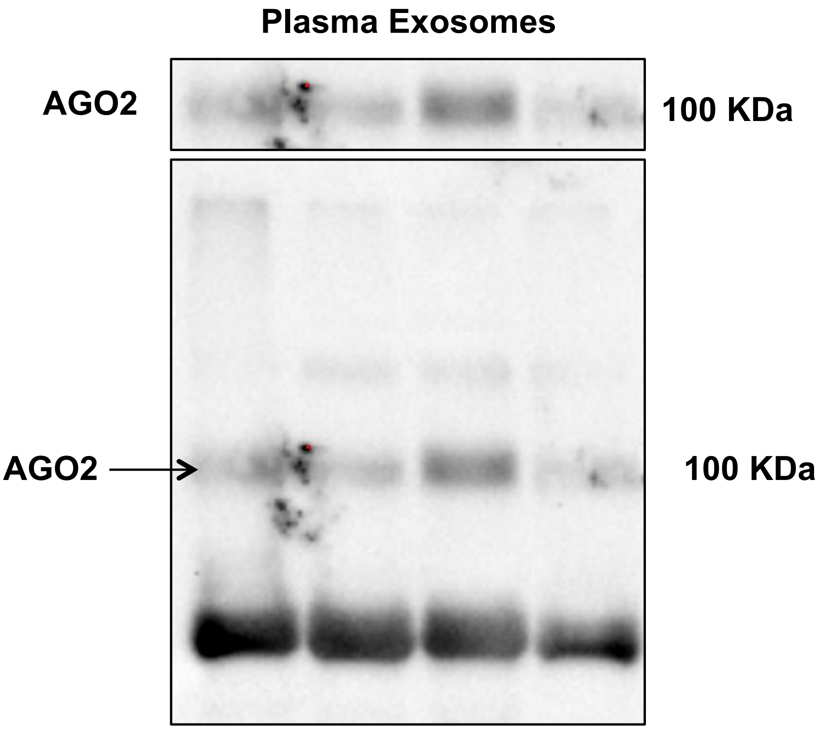

Full unedited gel for Supplementary Figure 2C
PF exosome

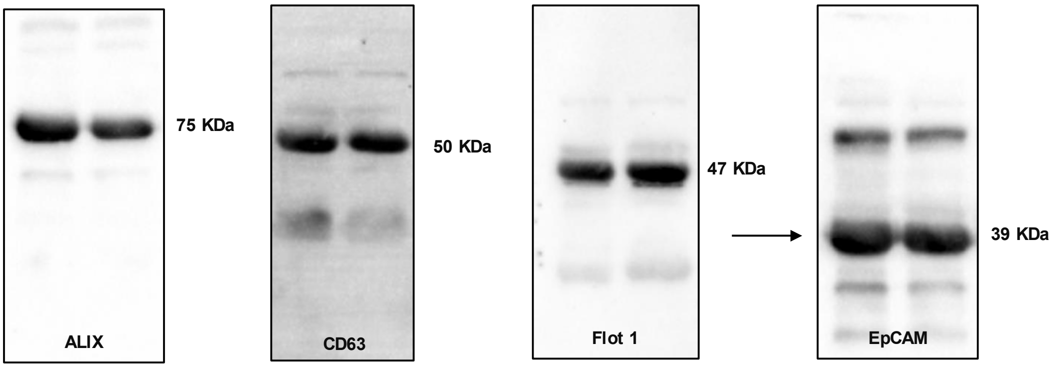

Full unedited gel for Supplementary Figure 2C
Plasma exosome

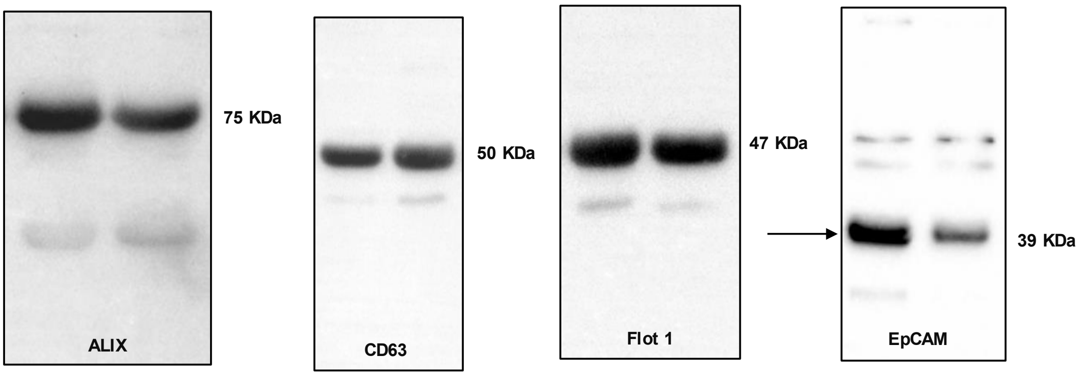

Full unedited gel for Figure 4A

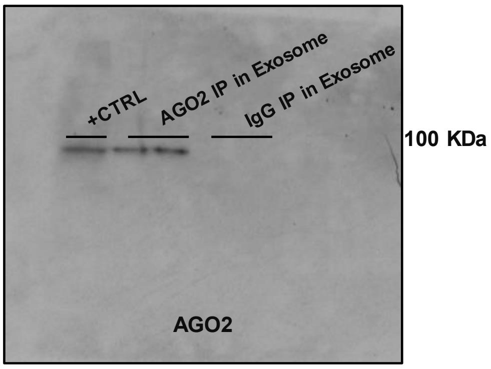

Full unedited gel for Figure 4C

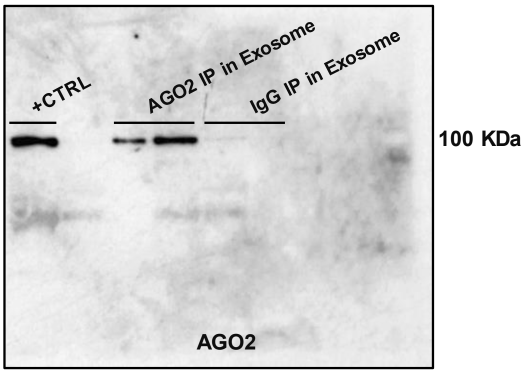

Full unedited gel for Figure 4E

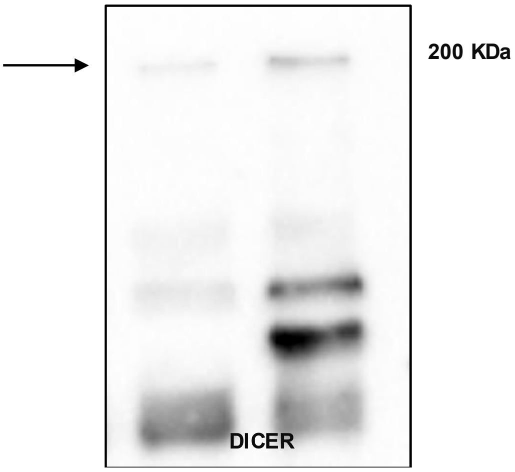

Full unedited gel for Supplementary Figure S3

PF Exosomes

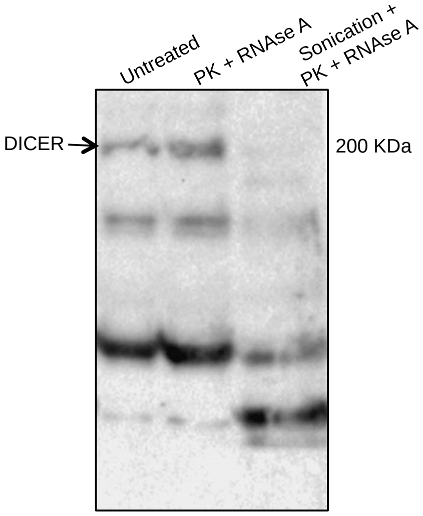

Plasma Exosomes

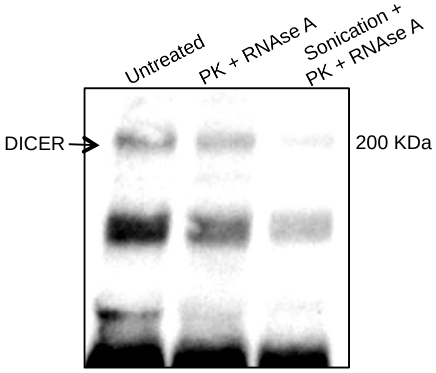

PF Exosomes

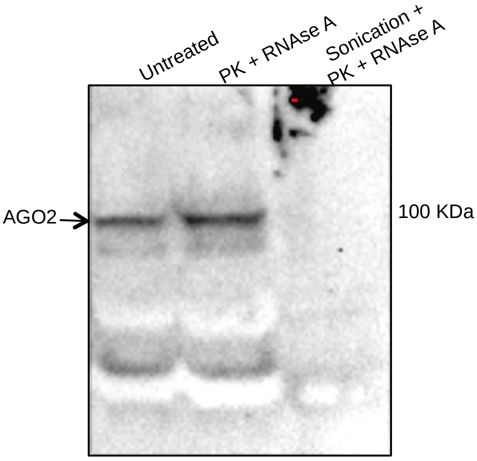

Plasma Exosomes

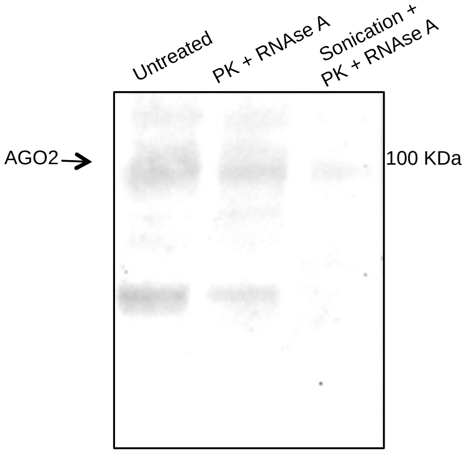

Full unedited gel for Supplementary Figure S9B

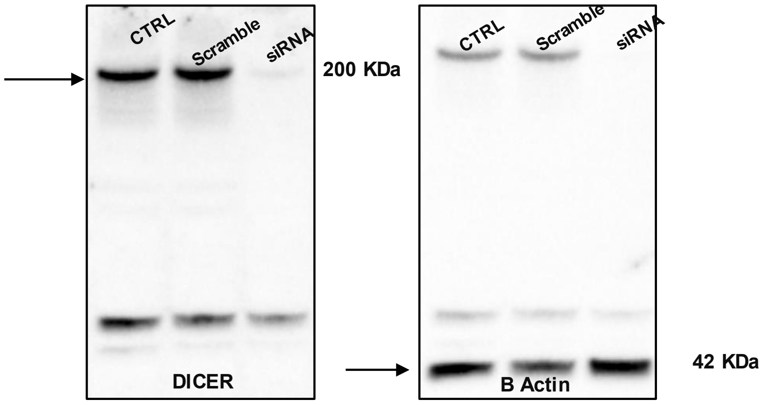

Full unedited gel for Supplementary Figure S10A

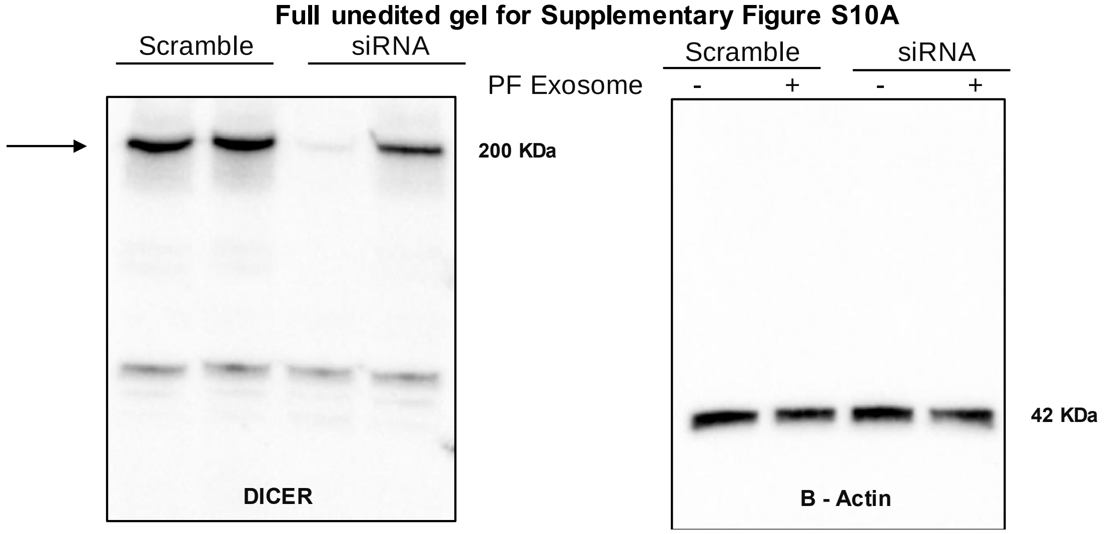

Supplement: Document S2. Article plus Supplemental Information [file mmc2.pdf]
